# Herbal remedies for oral and dental health: a comprehensive review of their multifaceted mechanisms including antimicrobial, anti-inflammatory, and antioxidant pathways

**DOI:** 10.1007/s10787-024-01631-8

**Published:** 2025-02-05

**Authors:** Mohamed A. Anwar, Ghadir A. Sayed, Dina M. Hal, Mohamed S. Abd El Hafeez, Abdel-Aziz S. Shatat, Aya Salman, Nehal M. Eisa, Asmaa Ramadan, Riham A. El-Shiekh, Shymaa Hatem, Shaza H. Aly

**Affiliations:** 1 Department of Pharmacognosy, Faculty of Pharmacy, Cairo University, Cairo, 11562 Egypt; 2 Department of Biochemistry, Faculty of Pharmacy, Egyptian Russian University, Badr, Cairo, 11829 Egypt; 3 Department of Pharmacognosy, Faculty of Pharmacy, Suez Canal University, Ismailia, 41522 Egypt; 4 Department of Pharmacy, Kut University College, Al Kut, Wasit, 52001 Iraq; 5 Department of Pharmacognosy, Faculty of Pharmacy, Egyptian Russian University, Cairo-Suez Road, Badr , 11829 Egypt; 6 Department of Pharmacology and Toxicology, Faculty of Pharmacy, Al-Azhar University, Cairo, Egypt; 7 Clinical Research Department at Giza Health Affairs Directorate, MOHP, Giza, Egypt; 8 Department of Biochemistry, Faculty of Pharmacy, Delta University for Science and Technology, Gamasa, Egypt; 9 Department of Pharmaceutics and Pharmaceutical Technology, Faculty of Pharmacy, Future University in Egypt, Cairo, Egypt; 10 Department of Pharmacognosy, Faculty of Pharmacy, Badr University in Cairo (BUC), Cairo, 11829 Egypt

**Keywords:** Nanotechnology, Dental care, Anti-inflammatory, Herbal remedies, Complementary medicine, Oral health

## Abstract

Across diverse cultures, herbal remedies have been used to alleviate oral discomfort and maintain dental hygiene. This review presents studies on herbal remedies with remarkable antimicrobial, anti-inflammatory, antioxidant, anticancer, anticaries, analgesic, and healing properties. The manuscripts demonstrate the depth of scientific inquiry into herbal remedies used for the management of various oral and dental health conditions. These include gingivitis, oral ulcers, mucositis, periodontitis, oral pathogens, carcinoma, xerostomia, and dental caries. Researchers have investigated the phytochemical and pharmacological properties of plant-derived compounds and their extracts evaluated their interactions with oral pathogens and inflammatory processes. The convergence of traditional knowledge and rigorous scientific investigation offers a compelling narrative, fostering a deeper understanding of herbal remedies as viable alternatives to conventional dental interventions. This work has the potential to provide patients with access to gentle, yet effective solutions, and simultaneously offer dental health professionals the opportunity to enrich their knowledge, and ability to provide personalized, holistic care. This review highlights the symbiotic relationship between herbal medicine and scientific understanding, emphasizing the importance of disseminating this knowledge to benefit both practitioners and patients, enabling evidence-based decision-making in dental care. The exploration of herbal remedies offers a promising alternative, potentially mitigating some of these side effects while promoting oral health in a more natural and holistic manner.

## Introduction

Oral diseases remain a significant global health issue, with dental caries and periodontal diseases being among the most critical challenges in oral health. Additionally, conditions like oral and pharyngeal cancers, as well as lesions of oral tissues, are also serious concerns. Oral health is essential for overall well-being and impacts quality of life in ways that go beyond the functions of the craniofacial structure (Anushri et al. 2015). Although some conditions, like cleft palate, cannot be prevented, there are effective strategies to reduce the risk of oral cancer and more prevalent Adental problems, such as gingivitis, pericoronitis (inflammation of the tissue around wisdom teeth), and severe periodontal disease, all of which can result in tooth loss. Moreover, inadequate oral hygiene has been linked to a higher risk of heart disease, underscoring the connection between oral health and overall well-being (Laudenbach and Kumar 2020; Odell 2024). Gingivitis and periodontal disease can be further aggravated by underlying medical conditions such as diabetes, making regular oral health monitoring crucial. Daily brushing and flossing provide an opportunity to inspect the mouth, tongue, and gums for any changes, and it is important to communicate any concerns to a healthcare professional. Early detection and intervention significantly enhance the likelihood of preventing complications and achieving favorable treatment outcomes (Dubey and Mittal 2020). Even seemingly minor issues, such as teeth grinding, can escalate into serious problems. Chronic grinding can lead to significant wear on tooth surfaces, jaw pain, and damage to teeth, including chips and fractures (PeaceHealth and a Bill, 2023). Dentists can offer guidance and solutions to help maintain dental health throughout one's life. Moreover, advancements in drug delivery systems, such as niosomes, phytosomes, cubosomes, and transdermal patches, present improved opportunities for enhancing the bioavailability, solubility, and permeability of natural plant constituents used in the treatment of dental diseases (Das 2022).

The anatomy of the mouth develops during the early stages of embryonic growth and serves multiple essential functions. The oral cavity is not only vital for communication and food intake, but also plays a critical role in the digestive process (Sterzenbach et al. 2020). It comprises various structures, including the hard and soft palates, mucosal tissues lining the upper and lower mouth, gingiva (gums), tongue, uvula, tonsils, and openings of the salivary glands, all of which contribute to its complex functionality (Shyam and Cohen 2021). Tooth brushing is a fundamental aspect of oral hygiene, and indigenous populations around the world utilize natural toothbrushes made from healing plants. These primitive twig "brushes" are surprisingly effective, providing disposable brushes with natural bristles that contain healing properties inherent to the plants themselves. Twigs possess volatile oils that stimulate blood circulation, tannins that tighten and cleanse gum tissue, and other beneficial substances like vitamin C that promote healthy gums. Plants such as bay, eucalyptus, oak, fir, and juniper are particularly effective for this purpose (Kumar et al. 2013).

Throughout history, humans have turned to nature for remedies to various ailments, and in recent years, herbal medicine has gained significant popularity in a variety of applications, including dietary supplements, energy drinks, multivitamins, massage therapies, and weight management products (Akter et al. 2021). This growing interest has not only expanded the field of herbal medicine, but has also enhanced its credibility and acceptance in modern healthcare.

In dentistry, herbal compounds are increasingly being utilized to address common issues such as tooth pain, gum inflammation, and oral lesions. Dental diseases rank among the most prevalent health concerns globally, with oral health being closely linked to overall quality of life, extending beyond mere functionality of the craniofacial complex (Kumar et al. 2022).

Herbal agents with antiseptic, antibacterial, antimicrobial, antifungal, antioxidant, antiviral, and analgesic properties are becoming essential tools in dental care (Dalir Abdolahinia et al. 2023). Conditions such as dental caries, periodontal disease, and endodontic infections are primarily caused by well-known bacterial and fungal pathogens, including *Streptococcus mutans*, *Porphyromonas gingivalis*, and *Candida albicans* (Singh et al. 2022). Preventive dental care predominantly focuses on maintaining oral hygiene to minimize bacterial biofilm formation. While chemical agents such as chlorhexidine, hyaluronic acid, and fluoride are commonly used in mouth rinses and toothpaste, they may come with clinical drawbacks, such as tooth discoloration, altered taste, dry mouth, and irritation of the oral mucosa (Hernández et al. 2022).

Research indicates that numerous herbs possess antibacterial, anti-inflammatory, and analgesic properties, making them valuable in managing conditions such as gingivitis, periodontitis, and toothaches (Rani et al. 2022). Commonly used herbs in dentistry include clove, known for its analgesic effects; aloe vera, which aids in healing and reducing inflammation; and peppermint, often used for its soothing properties and freshening breath. Other herbs such as myrrh, sage, and turmeric also contribute significantly to oral health by promoting gum health and reducing plaque formation (Dick et al. 2020).

Despite the historical use of these herbs, there remains a need for more rigorous scientific studies to fully understand their mechanisms of action and potential side effects. This exploration into herbal medicine not only highlights the importance of traditional practices, but also encourages a more comprehensive approach to oral healthcare, integrating both herbal and conventional methods for optimal patient outcomes.

## Search strategy

The search strategy involved a systematic approach utilizing multiple databases, including PubMed, Scopus, Web of Science, and Google Scholar. Key search terms such as "herbal remedies," "oral health," "dental health," "antimicrobial properties," "anti-inflammatory effects," and "antioxidant activity" were employed to maximize relevant literature retrieval. Inclusion criteria encompassed peer-reviewed articles published in English that focused on the effects of herbal remedies related to oral health and explored at least one of the main properties: antimicrobial, anti-inflammatory, or antioxidant. A total of 500 articles were retrieved and evaluated according to the inclusion criteria (in vitro, in vivo, ex vivo, and clinical studies). Following a thorough screening process, 358 research articles were selected for the review. Studies were excluded if they were a review article, letter, or conference abstract lacking sufficient details.

## Herbal remedies in dental care

Recent trends indicate that more people are opting to buy herbal products rather than consulting their doctors. Consequently, traditional healthcare providers may need to respond to the rising demand for herbal remedies driven by market changes. However, consumers who lack proper knowledge could encounter serious risks from the misuse of these products. Therefore, health educators must take on the responsibility of ensuring that individuals are well informed and engaged in their decisions about herbal medicine to protect public health (Sharif 2002). Oral diseases remain a significant global health concern, with dental caries and periodontal diseases being among the most pressing oral health challenges worldwide. Additionally, conditions like oral and pharyngeal cancers and lesions of the oral tissues are also serious issues. Oral health is essential to overall well-being and impacts quality of life in ways that go beyond the functions of the craniofacial structure (Anushri et al. 2015).

For centuries, herbs have been utilized to prevent and manage dental diseases. Herbal extracts are effective due to their interaction with particular chemical receptors in the body. Although herbal medicines typically have fewer side effects than conventional medications, they can still cause adverse reactions. It is crucial to recognize that the strength of herbal products can differ significantly (Buggapati 2016).

The worldwide demand for safe, effective, and affordable alternative prevention and treatment options for oral diseases has arisen due to several factors. These include the rising incidence of such diseases—especially in developing countries—the growing resistance of pathogenic bacteria to existing antibiotics and chemotherapeutics, the prevalence of opportunistic infections in immunocompromised individuals, and economic constraints faced by developing nations (Jena et al. 2021). Although various chemical agents are available on the market, they can disrupt oral microbiota and lead to unwanted side effects, including vomiting, diarrhea, and tooth discoloration. As a result, the quest for alternative products persists, with natural phytochemicals derived from plants used in traditional medicine viewed as promising substitutes for synthetic chemicals (Ege and Ege 2021).

Herbal products are increasingly utilized as sedatives, agents for reducing plaque, and for promoting healthy gums. These natural remedies present a promising way to support oral health while reducing the disadvantages linked to conventional treatments (Pandey et al. 2011).

Herbal compounds are being increasingly suggested for the treatment of serious conditions, including purulent gingivitis, mucositis, superficial periodontitis, catarrhal inflammation of the tongue, toxic oral cavity inflammation, mucosal infections, and difficult postoperative wound healing. Furthermore, herbal medications are also used to relieve oral symptoms related to systemic diseases (Szyszkowska et al. 2010). Plant-based compounds can be highly effective, especially in addressing inflammation caused by local irritants. Herbal medications are characterized by their anti-inflammatory, antiseptic, analgesic, astringent, edema-reducing, soothing, and wound-healing properties. The therapeutic benefits of these herbs stem from the biologically active compounds they contain. Many of these compounds have been isolated or extracted through bio-guided methods that identify the specific activities of different plant parts. Notable biologically active compounds in herbs include flavonoids, coumarins, iridoid glycosides, phenolic acids, resins, triterpenes, phytoesters, choline, carotenoids, tannins, vitamins, and mineral salts such as magnesium, iron, and lithium, as well as essential oils. Flavonoids and essential oils are particularly recognized for their healing properties and are commonly used in herbal treatments for oral health (Sinha and Sinha 2014).

The purpose of this review is to highlight recent examples of traditional medicinal plant extracts or phytochemicals that have demonstrated the ability to inhibit the growth of oral pathogens, reduce the development of dental plaque, and alleviate the symptoms of oral diseases. These natural products offer a promising alternative to conventional treatments, particularly in light of the increasing resistance to synthetic antimicrobials.

## Natural products in the management of gingivitis, ulcers, mucositis, and periodontitis

Gingivitis is an inflammatory disorder that impacts the gingival tissue, characterized by irritation and erythema of the gingiva. It primarily results from bacterial infection owing to deposits of plaque (Kurgan and Kantarci 2018). Certain plant-based components, such as tea tree oil, aloe vera, lemongrass and cloves, posses antibacterial and anti-inflammatory characteristics that may assist in alleviating gingivitis symptoms (Mosaddad et al. 2023). Mouth ulcers, referred to as canker sores or aphthous ulcers, are tiny, painful lesions that arise on the mucous membranes of the oral cavity. These ulcers may manifest on the inner cheeks, lips, tongue, gums, and the palate (Philipone and Yoon 2016). Another condition is known as mucositis, which is a prevalent adverse effect of cancer treatment, especially chemotherapy and radiation therapy, marked by inflammation and ulceration of the mucous membranes in the digestive tract, oral cavity, and pharynx. It may induce pain and discomfort, and impede eating and speaking (Naidu et al. 2004). Periodontitis represents a more advanced phase of periodontal disease than gingivitis. It entails inflammation surrounding the tooth, impacting the supporting tissues, including the gums, periodontal ligament, and alveolar bone. If neglected, periodontitis may result in tooth loss and other severe health complications (Lang et al. 2009; Slots 2017).

**Fig. 1:**
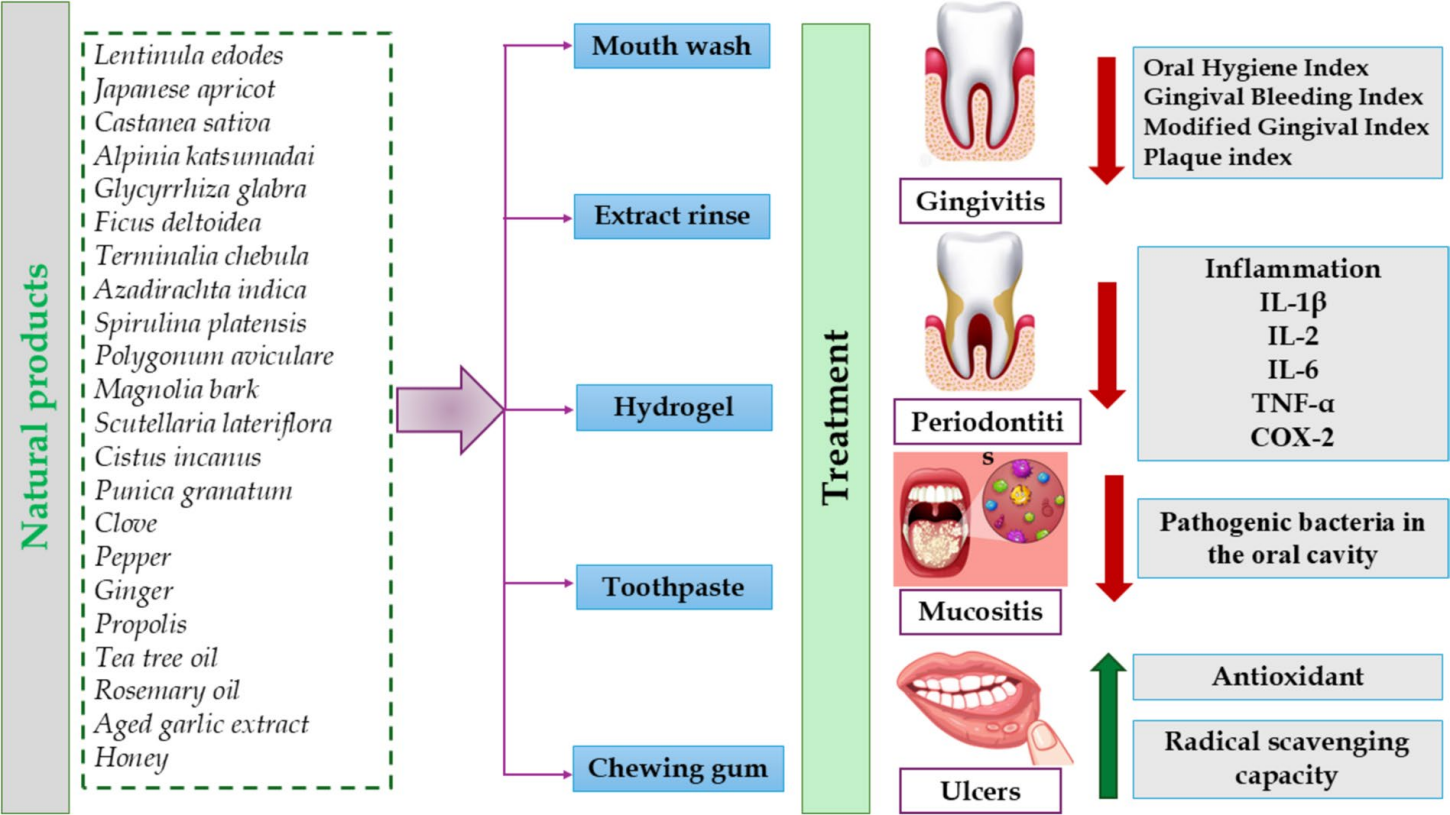
Schematic representation of the mechanism of action of natural products in the management of gingivitis, ulcers, mucositis, and periodontitis

Natural products provide a more comprehensive and potentially safer alternative to synthetic pharmaceuticals, as they may serve as supplements to conventional therapies or as preventive strategies for sustaining optimal oral health (Abdelazim et al. 2024; Aly et al. 2023). Specifically, they are particularly accessible and cost-effective, rendering them available to a broad demographic (Herman et al. 2005). Natural products provide an abundant supply of vital nutrients and bioactive metabolites such as flavonoids, alkaloids, phenolics, steroids, volatile oils and vitamins, minerals, and fiber, which are crucial for sustaining optimal health and maintaining antioxidant and anti-inflammatory properties (Aly et al. 2024; Cusumano et al. 2024; Goher et al. 2024; Zengin et al. 2024). Natural products they have received considerable interest in the treatment of numerous oral health conditions, including gingivitis, ulcers, mucositis, and periodontitis, owing to their potential therapeutic advantages (Ferreira et al. 2022a; Kumar et al. 2022; Salehi et al. 2019). For example, crocin is a carotenoid metabolite in the blooms of crocus and gardenia (Pfister et al. 1996). Crocin is recognized for its capacity to diminish oxidative stress and inflammation, which are major factors in periodontal disease. It may suppress the synthesis of pro-inflammatory cytokines, thereby mitigating tissue damage linked to periodontitis. Also, it regulates osteoclast–osteoblast equilibrium and promotes type 1 collagen accumulation in teeth and bone (Pfister et al. 1996). Figure 1 shows how natural products can play a role in the management of these conditions as indicated by in vitro, in vivo studies, and clinical trials (Fig. 1).

MK615 is an extract rich in triterpenoids derived from Ume, the Japanese apricot, which has served as a traditional Japanese medicine for centuries and is widely consumed as food (Nakagawa et al. 2007). In vitro investigations have examined MK615's effects on cell survival, migration, and inflammatory response in human gingival fibroblasts. The generation of pro-inflammatory indicators including interleukin (IL)-6 and IL-8 by gingival fibroblasts stimulated by LPS was reduced in a dose-dependent manner by MK615 by modulating cytokine production (Morimoto-Yamashita et al. 2015).

Another study aimed to assess the antigingivitis efficacy of *Lentinula edodes* extract, known as shiitake mushroom, by evaluating its efficiency against common bacterial infections linked to gingivitis. The shiitake mushroom extract exhibited considerable antibacterial efficacy against many bacterial strains, especially reducing the numbers of *Fusobacterium nucleatum* typically linked to gingivitis. The extract's efficacy was similar to conventional antiseptic chemicals employed in oral hygiene as chlorhexidine, suggesting its viability as a natural substitute (Ciric et al. 2011).

A recent investigation is focused on evaluating the potential of extracting beneficial chemicals from chestnut shells (*Castanea sativa*) for use in products for the treatment of oral mucositis. The extract showed superior antioxidant and antiradical properties, with scavenging efficiency against HOCl and ROO with IC_50_ values of 4.47 µg/mL and 0.73 µmol TE/mg DW, respectively. The antioxidant potential was associated with the phenolic profile of the chestnut extract, which was abundant in gallic acid, protocatechuic acid, epicatechin, catechin, and rutin. It demonstrated antibiotic activity against several oral bacteria present in the oral cavity during oral mucositis, particularly *Staphylococcus*, *Enterococcus*, *Streptococcus,* and *Escherichia*. Moreover, the chestnut shells revealed the lowest IC_50_ values of 1325.03 and 468.15 µg/mL against two human oral cancer cell lines, namely, HSC3 and TR146, respectively (Ferreira et al. 2022b).

Another study examines the therapeutic efficacy of ethanol extracts from *Alpinia katsumadai* seeds in the prevention and treatment of periodontitis, specifically targeting their impact on dental plaque bacteria including *Porphyromonas gingivalis* and inflammation. The extract suppressed RANKL-induced osteoclast development, resulting in reduced bone resorption, reduced COX-2 levels, and at concentrations ranging from 1 to 10 µg/mL the extract suppressed *P. gingivalis* growth on agar plates (Shin and Hwang 2021).

An Ayurvedic herbal extract consisting of a mixture of clove, ginger, Aleppo oak galls, black pepper,  heartwood of cutch tree, Spanish cherry bark, pongame oil tree root, and myrobalan fruit was investigated for its potential as anti-inflammatory responses associated with gingivitis and periodontitis. It markedly suppressed IL-8 expression in telomerase-immortalized gingival keratinocytes (TIGK) in response to all evaluated stimuli, either *Fusobacterium nucleatum* or pro-inflammatory cytokines (IL-1β and TNF-α), exhibiting a dose-dependent action that was not attributable to cytotoxicity (Chang et al. 2020).

An in vivo study examined the therapeutic efficacy of Kouyanqing Granule (KYQG) in the treatment of mouth ulcers induced by phenol and aggravated by sleep deprivation. KYQG markedly diminished inflammation and enhanced the healing of oral ulcers, as evidenced by reduced levels of inflammatory markers including COX-2, MMP-9, and TNF-α. The KYQG influences many pathways, notably through the suppression of inflammatory responses and the modulation of immunological function.

The TNF signaling pathway and HIF-1 signaling pathway are key involved signaling pathways (Chen et al. 2020). The UPLC–MS analysis of the KYQG indicated a high concentration of phenolics and flavonoids, with chlorogenic acid identified as the predominant component in the extract (Chen et al. 2020).

Another study developed a sustainable hydrogel composition with liquorice extract to enhance the healing of oral ulcers in rats, utilizing eco-friendly methods. The results revealed that liquorice extracts at a concentration of 30% in the hydrogel composition demonstrated enhanced wound healing results, comprising faster re-epithelialization, augmented collagen synthesis (27.8%), raised growth factor expression (23.24%), and diminished inflammation (88%). Liquorice possesses anti-inflammatory and antibacterial characteristics that enhance its efficacy in facilitating tissue repair and alleviating symptoms related to mouth ulcers. The efficacy is ascribed to glycyrrhizic acid, which constitutes the principal constituent of the extract (34.85 ± 2.77%) (Moussa et al. 2023).

The therapeutic efficacy of *Ficus deltoidea* leaf extract at a dose of 500 mg/kg in the treatment of oral ulcers was examined using an animal model. It diminished the dimensions of the oral ulcer and augmented the proportion of the inhibitory area (Ahmad and Amin 2017).

Individuals with cleft lip and palate frequently encounter distinct obstacles in sustaining oral hygiene due to anatomical variations and orthodontic interventions, rendering proficient oral care essential (Gaggl et al. 1999; Wong and King 1998). The study comprised 50 patients aged 9–16 years, categorized into two groups. Group A utilized toothpaste combined with propolis, tea tree oil, menthol, and rosemary oil vs. Group B as a placebo group. Gingival bleeding and oral hygiene indices were measured before and after 35 days. The findings demonstrated substantial enhancements in oral hygiene, evidenced by a decrease in the oral hygiene index (OHI-T) and a marked reduction in the gingival bleeding index (GBI) for both overall scores and particular tooth types (incisors and molars) in Group A, indicating improved gingival health. This outcome is ascribed to the antibacterial properties of propolis against oral infections, aiding in the reduction of plaque buildup and inflammation. Furthermore, combining it with essential oils aids in diminishing gum inflammation (Machorowska-Pieniążek et al.).

A study conducted by Zhang et al. evaluated the effectiveness of various mouthwashes in preventing oral mucositis (OM), a common and painful side effect of cancer treatments such as chemotherapy and radiotherapy. The study is based on utilizing a Bayesian network meta-analysis approach. The analysis indicated that honey mouthwash exhibited the greatest efficacy in preventing oral mucositis, followed by chamomile, curcumin, and benzydamine.

These mouthwashes demonstrated markedly greater efficacy than the placebo and exhibited superior outcomes relative to chlorhexidine, sucralfate, and povidone-iodine (Zhang et al. 2020).

The efficacy of honey on OM may be attributed to its hygroscopic properties, viscosity, or acidic pH, which inhibits bacterial proliferation on the mucosa, alongside tissue-nourishing minerals and vitamins that facilitate direct tissue repair. Moreover, honey is readily accessible, simple to gargle, mildly sweet, non-irritating, and is generally more palatable for patients, particularly children (Kocot et al. 2018; Ramsay et al. 2019). Besides, the effectiveness of chamomile may be attributed to its antimicrobial, anti-inflammatory, antioxidant, and anticancer properties (Sebai et al. 2014).

A comparative controlled randomized trial studied the use of a mouthwash containing *Terminalia chebula* fruit extract compared to chlorhexidine in reducing plaque accumulation and gingival inflammation. The extract of *T. chebula* fruit is recognized for its antibacterial and astringent characteristics, containing elevated levels of tannins (Bag et al. 2009). The findings indicated that both mouthwashes substantially diminished plaque index (PI) and gingival index (GI) scores following 4 weeks of use. The *T. chebula* extract mouthwash showed similar efficacy to chlorhexidine in managing plaque and alleviating gingival inflammation with fewer side effects such as staining of teeth and altered taste sensation (Gupta et al. 2015).

A clinical trial assessed the efficacy of aged garlic extract (AGE) in diminishing probing pocket depth (PPD) and enhancing gingival health over an 18-month duration. The average PPD for the AGE group dramatically declined from 1.89 ± 0.74 mm at baseline to 1.06 ± 0.49 mm at 18 months (*p* < 0.001) (Zini et al. 2020). AGE comprises sulfur compounds, including *S*-allylcysteine, *S*-1-propenylcysteine, and *S*-allylmercaptocysteine, which exhibit antioxidant properties that enhance the efficacy of AGE in addressing periodontitis. It also improves peripheral circulation, diminishes inflammation, and augments immunological abilities (Ushijima et al. 2018; Xiao et al. 2012).

A further study administered AGE to dogs with mild gingivitis at a dosage of 18 mg/kg/day for 8 weeks. The results indicated that the dogs with improved gingival index scores did not demonstrate an increase in volatile sulfur compounds (VSCs), typically linked to bad breath and periodontal disease, whereas the placebo group exhibited elevated levels at 8 weeks. The outcomes are ascribed to the anti-inflammatory and antibacterial characteristics of AGE, possibly aiding in the reduction of inflammation and bacterial presence in the oral cavity (Takahashi et al. 2023).

Another investigation attempted to evaluate the effectiveness of a mouth rinse derived from *Azadirachta indica,* known as neem leaves in diminishing clinical manifestations of gingivitis, particularly probing depth and gingival inflammation. Results showed that individuals utilizing the neem mouthrinse exhibited notable decreases in gingival index scores and probing depth relative to those utilizing the placebo owing to the antimicrobial characteristics of the neem mouthwash that enhance oral hygiene and diminish irritation. It also has the advantage of being well accepted by participants, with negligible side effects observed during the trial (Botelho et al. 2008).

Spirulina (*Spirulina platensis*) is a photosynthetic cyanobacterium possessing various biological activities. It comprises different chemical compounds such as phenolic compounds, tocopherols, beta-carotenes, and phycocyanins that exhibit antioxidant effects.

Phycocyanin, a principal component of spirulina, exists as a complex mixture of trimers and hexamers. It also has antioxidant properties, elucidating its potent anti-inflammatory impact (Mahendra et al. 2013; Nuhu 2013). Participants receiving spirulina supplementation demonstrated notable decreases in inflammatory markers linked to chronic periodontitis. Clinical indicators, including probing depth and gingival index, demonstrated improvements, signifying increased periodontal health. Spirulina augments the function of antioxidant enzymes, including superoxide dismutase (SOD) and glutathione peroxidase (GSH-Px), which are essential for mitigating oxidative damage in periodontal tissues (Kaipa et al. 2022).

A study by Howshigan et al. examined the effectiveness of an Ayurvedic toothpaste containing nine medicinal plants in treating persistent gingivitis in comparison to a placebo. The nine herbal components in the Ayurvedic toothpaste known as Sudantha® are *Zingiber officinale* Rosce, *Syzygium aromaticum* L., *Piper nigrum* L., *Terminalia chebula* Retz., *Mimusops elengi* L., *Pongamia pinnata* (L.) Pirerre, *Acacia chundra* Willd., *Adhatoda vasica* Nees., and *Quercus infectoria* Olivier. Participants utilizing the Ayurvedic toothpaste exhibited substantial decreases in plaque index, bleeding on probing, and probing pocket depth relative to the placebo group (*p* < 0.0001). Microbiological evaluations revealed a notable reduction in total salivary anaerobic bacterial counts in the Ayurvedic toothpaste group vs the placebo (*p* < 0.05). The enhancements in clinical indicators progressed throughout time, signifying enduring advantages from the use of the Ayurvedic formulation (Howshigan et al. 2016).

A controlled, randomized study examined the therapeutic effects of a mouthwash containing a Mexican Sanguinaria extract (*Polygonum aviculare*) on individuals with gingivitis. It demonstrated substantial enhancements in clinical indicators, including decreased gingival bleeding and inflammation relative to the placebo group. Additionally, antibacterial and anti-inflammatory properties enhanced its efficacy in the management of gingivitis (Begné et al. 2001). Research involving sanguinarine, an alkaloid derived from *Sanguinaria canadensis*, has indicated that this extract may be very beneficial for the prevention of plaque and gingivitis (Croaker et al. 2016).

A randomized controlled study involving individuals who chewed sugar-free gum with magnolia bark extract showed a reduction in dental cavities and gingival irritation compared to regular gum (Campus et al. 2011). It also showed antibacterial capabilities that reduced the levels of oral microorganisms linked to caries and gingivitis. The primary components of the bark are magnolol and honokiol, which exhibit antibacterial and anti-inflammatory properties that may aid in diminishing bacterial load and inflammation in periodontal tissues (Teng et al. 1990).

A recent research study examined the impact of a chewing gum formulation including extracts from *Scutellaria lateriflora* and *Cistus* × *incanus* on symptoms of gingivitis in a double-blind, placebo-controlled clinical trial for 3 months. Results revealed that the modified gingival index (MGI), quantitative gingival bleeding index (QGBI), and Oral Health 15 items (OH-15) were reduced by the 3rd month of treatment as compared to placebo (Di Minno et al. 2024).

DiSilvestro et al. conducted a study aimed to assess the impact of pomegranate (*Punica granatum* L.) extract rinse on salivary indicators related to dental health and the risk of gingivitis. The research included 32 young adult participants who gargled with a pomegranate extract diluted in water three times daily for 4 weeks. The results revealed that the participants exhibited a major decrease in total protein levels in saliva, which coincides with a reduction in plaque-forming bacteria.

A significant reduction in AST and α-glucosidase activity was observed, suggesting less cellular damage and a decline in sucrose breakdown, respectively. Also, elevations in ceruloplasmin activity and radical scavenging capacity were noted, indicating improved antioxidant defense against oxidative stress. These findings indicate the incorporation of pomegranate extracts as a potent and effective candidate for oral hygiene products (DiSilvestro et al., 2009).

## Natural products in the management of xerostomia, caries, and oral carcinoma

In recent years, there has been a notable increase in the exploration of alternative and natural therapies for managing xerostomia. These natural remedies not only address the symptoms of dry mouth, but also mitigate complications associated with the condition, such as dental caries xerostomia and oral tumors (Motallaei et al. 2021; Nieuw Amerongen and Veerman 2003).

Xerostomia, commonly referred to as dry mouth, is a clinical condition characterized by a significant reduction in saliva production, leading to discomfort and increased susceptibility to oral infections, including dental caries. This condition can result in dysphagia (difficulty swallowing) and is associated with various etiological factors, such as medications that induce xerostomia, radiation therapy targeting the head and neck, and autoimmune disorders like Sjögren’s syndrome (Kontogiannopoulos et al. 2023). Among the most extensively studied natural products are thyme honey, apigenin, ethanolic extracts of *Ixeris dentata* (IXD), *Lycium barbarum* polysaccharides (LBP), green tea, fermented lingonberry juice, coconut oil, ginger, aloe vera, peppermint, glucosylceramide from pineapple, linseed, *Malva sylvestris*, *Alcea digitata*, pilocarpine, and lycopene-enriched virgin olive oil (Ibrahim et al. 2023). In this context, thyme honey has emerged as a promising remedy for severe dry mouth, particularly in older adults with end-stage kidney disease. Clinical studies indicate that thyme honey oral rinses can stimulate salivary flow without adverse effects. Furthermore, it has shown efficacy in alleviating dry mouth symptoms associated with radiation therapy in patients with head and neck cancer (Kontogiannopoulos et al. 2023) (Ibrahim et al. 2023). Virgin olive oil, recognized for its health benefits, has been utilized as a spray enriched with lycopene—a carotenoid beneficial for xerostomia patients undergoing treatment. Although this intervention did not significantly increase saliva output, it provided relief from dry mouth discomfort (Navarro Morante et al. 2017). In addition, the ethanolic extract of *Ixeris dentata* has been investigated in diabetic xerostomic rat models. Results indicated improved salivary production and enzyme levels alongside reduced blood glucose concentrations. This suggests a potential therapeutic role for IXD extract in managing xerostomia (Kontogiannopoulos et al. 2023). Research on *Lycium barbarum* (Goji Berry) highlights its ability to promote salivation in Sjögren’s syndrome models. Low doses of LBP have been shown to stimulate salivary flow and reduce inflammatory responses, positioning it as a viable treatment option for xerostomia. (Kontogiannopoulos et al. 2023). Green tea (*Camellia sinensis*) is rich in polyphenols such as epigallocatechin-3-gallate (EGCG), which may aid in managing dry mouth symptoms. A specialized formulation known as MighTeaFlow has demonstrated effectiveness in alleviating xerostomia symptoms through the supplementation of green tea catechins (Kontogiannopoulos et al. 2023). Fermented lingonberry juice (FLJ) has attracted attention for its ability to enhance salivary pH and stimulate salivation. Its antioxidant and anti-inflammatory properties contribute to its potential utility in treating dry mouth symptoms; however, further research is warranted to confirm these findings (Kontogiannopoulos et al. 2023). Coconut oil (*Cocos nucifera*) has proven effective for patients experiencing dry mouth due to radiation therapy. Its unique surface-active properties help retain moisture within the oral cavity (Kontogiannopoulos et al. 2023). Ginger (*Zingiber officinale*) stimulates salivation by activating the parasympathetic nervous system through its action on M3 muscarinic receptors. This herbal remedy has long been employed for managing dry mouth symptoms (Kontogiannopoulos et al. 2023). Preparations combining *Aloe vera* and peppermint have been developed into gels that alleviate dry mouth symptoms while reducing plaque accumulation and improving overall oral health (Kontogiannopoulos et al. 2023). Glucosylceramide, derived from pineapple (*Ananas comosus*), enhances mucosal cell proliferation and ceramide production within the oral cavity, presenting itself as a natural alternative to conventional moisturizers (Kontogiannopoulos et al. 2023). Linseed (*Linum usitatissimum* L.) extract and specifically Salinum®—a viscous polysaccharide containing liquid—was used for the prevention of dry mouth caused by radiation therapy. The use of this extract by patients has led to improvements in oral hygiene and the health of the mucosa (Kontogiannopoulos et al. 2023). Traditional Persian medicine recognizes the anti-inflammatory and moisturizing properties of *Malva sylvestris* and *Alcea digitata*, which have been effective in treating radiation-induced xerostomia by promoting hydration and stimulating salivary gland function (Kontogiannopoulos et al. 2023). Pilocarpine, a plant-derived sialagogue commonly prescribed for xerostomia—especially in Sjögren’s syndrome—acts via M3 muscarinic receptors to enhance salivation (Kontogiannopoulos et al. 2023). Lastly, apigenin, a flavonoid found in various plants, has garnered interest due to its potential role in stimulating salivary gland function through estrogen receptor modulation (Kontogiannopoulos et al. 2023). In summary, an increasing body of research underscores the efficacy of natural products in managing xerostomia. These alternative therapies present attractive options for patients seeking less invasive treatments with favorable effects on saliva secretion and oral health while minimizing side effects associated with conventional pharmacological interventions.

Tooth decay, also reckoned as caries, is an infectious disease of the tooth that destroys the enamel and dentin layers of the tooth (Wilson et al. 2021). The process of decay begins with the activity of bacteria found in the mouth that use the sugars present and produce acids which then attack the teeth. Treatment strategies mainly involve improvement of oral care habits, spitting out, and reducing the use of sugar-containing beverages (Yadav and Prakash 2017). Several studies have reported effective remedy for dental caries using various medicinal herbs such as *Lespedeza cuneata*, *Sambucus williamsii* var. coreana, *Glycyrrhiza uralensis*, *Vaccinium macrocarpon*, Uva-fugi apple, *Vitis vinifera* (red grape seeds), *Myristica fragrans* nutmeg, ajwain *Trachyspermum ammi*, *Coffea arabica* and *Coffea robusta*, *Hordeum vulgare* (barley coffee), *Myrtus communis*, *Allium sativum* (garlic), *Theobroma cacao* (cocoa), propolis, and *Camellia sinensis* (tea). *Lespedeza cuneata* is included in mouthwash products and has been reported to lower the acid-producing activity of oral bacteria responsible for dental caries. This helps to lower the microbial load in the oral cavity, hence preventing any chances of dental caries. This mouthwash extract serves as an excellent protective measure against the development and progression of dental cariesdue to the absence of harmful chemical ingredients (Kim and Nam 2022c). A clinical trial demonstrated that mouth rinsing with a preparation containing *Sambucus williamsii* var. coreana extract is effective in fighting dental caries as it reduces the proportion of *Streptococcus mutans* in the mouth. The results showed that after 5 days of continuous treatment with mouthwash, the number of bacteria rapidly decreased. Moreover, there were no side effects after the treatment including any increase in corrosive agents, suggesting its potential in the prevention of tooth cavities (Kim and Nam 2022c). Research on *Glycyrrhiza uralensis* confirms its capacity to inhibit cariogenic bacteria, particularly *Streptococcus mutans*, making it a valuable component in mouthwashes and sugar-free lollipops designed to mitigate bacterial activity (He et al. 2006; Kim and Nam 2021, 2022a; Sidhu et al. 2020). The polyphenols found in cranberries, especially A-type proanthocyanidins, have been shown to influence key virulence factors of caries-related microbes by inhibiting acid production and bacterial adhesion to tooth surfaces (Philip and Walsh 2019). Additionally, compounds from apples have demonstrated the ability to suppress water-insoluble glucan production essential for bacterial retention on teeth, thus highlighting their potential in caries prevention (Gazzani et al. 2012). Extracts from red grape seeds exhibit significant antibacterial activity, with minimum inhibitory concentrations as low as 0.5 mg/ml, effectively inhibiting oral bacteria (Gazzani et al. 2012). Additionally, nutmeg (*Myristica fragrans*) extracts, and particularly the compound macelignan can be considered a potent anticaries ingredient. Macelignan has been reported to kill *Streptococcus mutans* at low concentrations and to inhibit its biofilm formation, thus being an effective substance against tooth decay (Gazzani et al. 2012). Similarly, the extract of ajowan caraway (*Trachyspermum ammi*) contains naphthalene derivative which is a high anticaries agent. This agent inhibits the adherence of bacteria as well as the formation of biofilm and acid production, thereby becoming a useful natural product in caries management (Gazzani et al. 2012). Roasted coffee, especially because of its α-dicarbonyl compounds, exhibited inhibitory activity toward *Streptococcus mutans*. Moreover, both extracts of roasted and unroasted coffee were found to possess antiadhesive properties, preventing the attachment of bacteria onto the tooth surface and thus minimizing caries development (Gazzani et al. 2012). Barley coffee has been found to possess the ability to prevent the adherence of bacteria and the formation of their biofilms. Research suggests that at concentrations below those that inhibit bacterial growth, barley coffee is capable of decreasing formation of biofilms induced by *Streptococcus mutans*, making it a candidate for a natural treatment agent for dental caries (Gazzani et al. 2012). Myrtle (*Myrtus communis*) leaf extract is found to be used as a folk remedy in Mediterranean regions. Ethanol extracts of myrtle leaves have shown a significant antibacterial effect, reducing the microbial load in the oral cavity after a single application which can probably be attributed to the flavonoids present in the extracts (Gazzani et al. 2012). Seven days of garlic-containing mouthwash was sufficient to lower the levels of *Streptococcus mutans* in salivary samples. This shows that oral healthcare products containing garlic raw material are feasible and efficacious (Gazzani et al. 2012). Cocoa, in particular husk’s extract, has exhibited potent antibacterial activity. It has been revealed that cocoa polyphenols help inhibit the formation of plaque as well as acid production, which are contributory factors to the occurrence of dental caries. In clinical studies, mouthwash using cocoa bean husk extract showed a marked decrease in plaque accumulation and bacteria count (Gazzani et al. 2012). Propolis has been fully characterized as an antimicrobial agent against *Streptococcus mutans* in vitro and in vivo. Its flavonoids and sesquiterpenes help in the reduction of biofilm and bacteria attachment, and when propolis is administered together with fluoride it had been effective in preventing dental caries (Gazzani et al. 2012). The association of tea, black and green, with oral in healthy possesses beneficial influence has been a widespread practice for quite a long time. EGCG, one of the green tea catechins, was specifically found to inhibit glucosyltransferase enzyme activity. Studies show that caries rate is decreased by green tea. In addition, black tea's higher polymerized polyphenol content and fluoride levels further enhance its anticariogenic properties (Gazzani et al. 2012). In summary, these natural substances, through their antibacterial, antiadhesive, and biofilm-reducing properties, offer promising alternatives to traditional chemical treatments for the prevention and management of dental caries (Bagan et al. 2010).

Oral carcinoma, commonly referred to as oral cancer, is a malignant condition characterized by the destruction of tissues in the mouth and throat. Key risk factors for this disease include tobacco use, excessive alcohol consumption, and infections with human papillomavirus (HPV). Early diagnosis is crucial for effective treatment and significantly influences the prognosis and survival rates of affected individuals (Zygogianni et al. 2011). Recent studies have highlighted the potential of various natural compounds in the management of oral carcinoma. These compounds not only exhibit anticancer properties, but also possess additional benefits that may enhance oral health. Research indicates that zinc oxide is encapsulated using cinnamic acid, as a stabilizing agent, and exhibits significant antioxidant and antimicrobial activities. Furthermore, these nanoparticles demonstrate dose-dependent cytotoxicity against human oral epidermal carcinoma KB cells by modulating apoptotic pathways involving critical proteins such as BCL-2, BAX, and P53 (Ravikumar et al., 2024). Another formulation combines curcumin with zinc oxide nanoparticles. Evaluated for its antioxidant, antimicrobial, and anticancer properties, the formula has shown promising results in apoptosis assays and gene expression studies against KB oral squamous carcinoma cells. This dual-action approach addresses both infectious agents and cancerous cells (Tayyeb et al. 2024). Essential oils derived from various plants within the Zingiberaceae family such as *Curcuma mangga*, *Curcuma xanthorrhiza*, *Kaempferia galanga*, and *Curcuma aeruginosa* have demonstrated antibacterial activity against specific oral pathogens. For instance, *Curcuma xanthorrhiza* oil exhibits activity against *Streptococcus mitis* and *Streptococcus sanguinis*, while *Curcuma mangga* oil inhibits *Streptococcus mutans*. Additionally, these oils show moderate cytotoxic effects against oral cancer cells, suggesting their potential utility in natural oral care products (Amil et al. 2024). Probiotic strains such as *Saccharomyces boulardii* and *Saccharomyces cerevisiae* have been identified as effective agents against dental caries caused by the interaction between *Streptococcus mutans* and *Candida albicans*. These probiotics not only inhibit fungal growth but also help maintain a balanced pH in the oral cavity, thereby protecting dental enamel. Notably, *S. boulardii* has been shown to downregulate key genes expressed in *S. mutans*, enhancing its potential for caries prevention (Yousif et al. 2024). Extracts from European spruce needles are rich in biologically active compounds (BACs), particularly phenolic and flavonoid components. Luteolin, a prominent flavonoid within these extracts, exhibits significant antitumor activity against squamous carcinoma cells in vitro (Tylkowski et al. 2022). Innovative formulations incorporating mucoadhesive patches that utilize hydroxypropyl methylcellulose (HPMC) combined with ethanol extract of *Usnea barbata* have been developed. These patches demonstrate anticancer properties by inducing oxidative stress and inhibiting DNA synthesis in cancer cells while also exhibiting antimicrobial activity against pathogens such as *Staphylococcus aureus*, *Pseudomonas aeruginosa*, *Candida albicans*, and *Candida parapsilosis*. The dual functionality of these patches positions them as viable candidates for both prophylactic and therapeutic applications in oral diseases (Popovici, Violeta et al., 2022).

## Natural products in miscellaneous dental disorders

Oral lichen planus (OLP) is a chronic autoimmune condition primarily affecting middle-aged women, characterized by painful lesions in the buccal mucosa. Conventional treatments, often involving corticosteroids, can lead to side effects, prompting interest in alternatives like curcumin from turmeric. Curcumin exhibits anti-inflammatory, antioxidant, and antimicrobial properties, with clinical trials showing its efficacy in alleviating OLP symptoms through high doses and topical formulations. Despite challenges with bioavailability, nano-curcumin formulations have improved absorption. While corticosteroids remain standard treatment, curcumin's potential as a safe alternative for managing OLP is promising and warrants further research (Khosrojerdi et al. 2023). This randomized double-blind study evaluated the effectiveness of topical aloe vera (AV) for treating oral lichen planus (OLP). Patients applying AV three times daily reported significant improvements in pain levels, lesion severity, and overall quality of life, particularly in psychological disability and total Oral Health Impact Profile (OHIP-49) scores. Although the AV group showed greater pain relief compared to the placebo group, these differences were not statistically significant. Notably, 61.29% of the AV group achieved complete recovery by the study's end, compared to 41% in the placebo group, with no adverse effects reported. The findings suggest that AV may serve as a safe alternative treatment for OLP, highlighting the need for further research to confirm its efficacy and explore its preventive applications (Salazar‐Sánchez et al., 2010). This study evaluated the clinical efficacy of topical chamomile gel compared to a placebo in treating OLP in 60 patients. Participants received either 2% chamomile gel or a placebo three times daily for 4 weeks. Results showed significant improvements in pain, burning sensation, itching, and overall oral health in the chamomile group, with 92% experiencing partial or total responses versus 17% in the placebo group. No adverse effects were reported. The findings suggest that chamomile gel may be an effective alternative treatment for OLP, offering a low-cost and accessible option with minimal side effects compared to traditional therapies. Further long-term studies are recommended to confirm these results (Lopez Jornet and Aznar‐Cayuela, 2016).

Oral submucous fibrosis (OSF) is a chronic disease primarily caused by areca nut chewing, leading to inflammation and fibrosis of the oral mucosa. This study investigates the combined use of curcumin, an anti-inflammatory polyphenol, with intralesional dexamethasone and hyaluronidase for treating OSF. A 12-week randomized clinical trial with 34 patients showed that curcumin significantly improved mouth opening, and reduced symptoms compared to standard treatment. The findings suggest curcumin's potential as an effective adjunct therapy for OSF, warranting further research (Adhikari et al. 2022).

Recurrent aphthous stomatitis (RAS) affects 5%–25% of the population, presenting as painful oral ulcers categorized into minor, major, and herpetiform forms. Minor ulcers are the most common, typically healing within 10–14 days, but causing significant discomfort. The exact cause of RAS is unclear, with factors like hypersensitivity, nutritional deficiencies, and stress implicated. Current treatments focus on pain relief, but no single therapy is fully effective. This study explored the efficacy of myrtle (*Myrtus communis*) extract in a randomized trial involving 45 participants. Results showed that myrtle significantly improved ulcer healing and pain relief compared to placebo, with no adverse effects reported. The findings suggest that myrtle extract could be a safe and effective treatment for RAS, warranting further research into its preventive applications during the prodromal phase of ulcer development (Babaee et al. 2010).

Oral submucous fibrosis (OSMF) is a chronic and debilitating condition characterized by fibrosis in the oral cavity, leading to stiffness and reduced mouth opening. Defined by Pindborg and Sirsat, OSMF can affect any part of the oral cavity and sometimes the pharynx, often associated with inflammation and epithelial atrophy (More et al. 2020). This condition is considered precancerous and can progress to oral cancer if untreated. Symptoms include difficulty eating, burning sensations, and potential malnutrition. Increased awareness and research are essential for effective management and treatment strategies to improve patient outcomes and quality of life (Xu et al. 2021). A study developed a herbal muco-adhesive gel containing 1% curcumin and licorice to treat OSMF in a rat model induced by bleomycin injections. Histological analysis confirmed the model's fibrotic features. Treatment with the gel significantly improved mouth opening and reduced fibrosis, especially in weeks 12 and 16, attributed to the anti-inflammatory and antifibrotic properties of curcumin and licorice. The findings suggest that this herbal gel could be a therapeutic alternative for managing OSMF, warranting further research for clinical applications (Ejaz et al. 2022).

Denture stomatitis (DS), also referred to as denture sore mouth or stomatitis prosthetica, is a prevalent condition among denture wearers, particularly those with inadequate oral hygiene. Characterized by localized or widespread erythema in the oral mucosa beneath dentures, it is more frequently observed in women and affects the maxillary arch. While discomfort is often minimal, DS has a prevalence ranging from 11 to 72%, making it a significant concern for denture users. Ill-fitting dentures can cause mucosal trauma, increasing the risk of colonization by opportunistic microorganisms, particularly *Candida* species (Gorji et al. 2024). A study explored the antifungal properties of betel quid and its components, particularly focusing on its effectiveness against DS. A clinical trial compared nystatin mouthwash and garlic extract, finding both effective in treating DS, but nystatin led to faster recovery with more side effects and lower patient satisfaction due to its bitter taste. The research highlighted garlic extract as a viable alternative, supported by its antimicrobial properties attributed to allicin. Overall, the findings suggest that garlic extract could serve as an effective adjunct or alternative therapy for oral fungal infections, warranting further clinical investigation (Bakhshi et al. 2012).

## The role of herbal extracts in dental treatment  

Natural products exhibit a wide range of bioactive compounds, including flavonoids, terpenoids, alkaloids, and phenolic compounds, which have demonstrated antimicrobial, wound healing, anti-inflammatory, and antioxidant activities. These properties are crucial in combating periodontal pathogens such as *Porphyromonas gingivalis*, *Aggregatibacter actinomycetemcomitans*, and others that contribute to periodontal disease progression (López-Valverde et al. 2023).

Several pathogens, such as *Staphylococcus mutans*, *Staphylococcus aureus*, *Enterococcus faecalis*, and *Candida albicans*, represent main causative agents of different oral diseases (Ravikumar et al., 2024). *S. mutans* is a Gram-positive, facultatively anaerobic bacterium, mostly reside in fissures and pits. It is an opportunistic commensal bacterium that causes dental caries (the most common chronic disease worldwide) especially the early childhood caries (Falsetta et al. 2014; Morrison et al. 2023). *C. albicans* is a commensal yeast fungus of the human skin and oral, gastrointestinal, as well as genital mucous membranes (Lopes and Lionakis 2022). It accounts for up to 95% of oral candidiasis infection (Vila et al. 2020). In vitro and  in vivo models revealed that the co-infection with *S. mutans* and *C. albicans* enhanced the pathogenesis of dental caries. This symbiotic relationship enhances the biofilm formation as *C. albicans* induce exopolysaccharides and support the viability of *S. mutans* than single-species biofilm (Falsetta et al. 2014). As *S. mutans* enhance its biofilm formation by the aid of *C. albicans*, the oral streptococci reinforce *C. albicans’s* invasive characters (Radaic and Kapila 2021). Periodontal diseases are complex, polymicrobial inflammatory infections characterized by tooth-supporting tissues destruction. The disease begins as acute inflammation of the gingiva (gingivitis) and, if untreated, proceeds to form teeth pockets, and eventually teeth loss. *Porphyromonas gingivalis*, a Gram-negative anaerobe, is the major contributor to chronic periodontitis (How et al. 2016). *Prevotella* are Gram-negative, anaerobic bacteria that normally colonize the oral cavity. The species of this genus, viz*.*, *P. dentalis*, *P. fusca*, *P. enoeca*, *P. denticola*, *P. melaninogenica*, *P. intermedia 17*, and *P. intermedia 17–2*, induce inflammatory cytokine production that contributes also to periodontal diseases (Morrison et al. 2023). Another dental issue is halitosis which is a condition characterized by oral malodor. Bacteria such as *Porphyromonas gingivalis*, *Treponema denticola*, *Prevotella intermedia*, *Porphyromonas endodontalis*, *Fusobacterium nucleatum*, and *Solobacterium moorei* contribute in this case (Prabhu et al. 2022). This bad breath is caused by volatile sulfur compounds (VSCs) that resulted from protein degradation by halitosis bacteria (Liu et al. 2022). All these mentioned microbes with other ones (up to a total of 1000 species) belong to bacteria, fungi, protozoa, archaea, and viruses comprising the oral microbiota which is a community of microorganisms residing in the oral cavity. The microbiota of healthy hosts in normal conditions maintains balanced symbiotic/commensal relationships which we refer to as eubiosis or microbial homeostasis (Radaic and Kapila 2021). Changes or insults such as tobacco smoking, immunosuppression, and diet alteration may shift this eubiosis to what is called dysbiosis, which is a state of parasitic/pathogenic relationship that promote diseases like dental caries and periodontitis, among others (Lamont et al. 2018; Radaic and Kapila 2021). New drug delivery technologies such as nano systems may aid in diagnosis, prevention, and care of dentistry diseases (Agnihotri et al. 2020). Nanomaterials can enhance the performance of various dental hygiene products such as toothpastes, mouthwashes, and polishing pastes. They range from 1 to 100 nm in size and their extraordinary surface-to-volume ratio increases their bioavailability toward cells and tissues (Carrouel et al. 2020). Nanoparticles of metals have been incorporated into the dental materials to enhance the regenerative, mechanical, and antimicrobial properties. For example, Zn, Ag, Au, Cu, and Ti have antibacterial activity (Agnihotri et al. 2020). Another technique is photodynamic therapy that uses a combination of light of specific wavelength and photosensitizer drug in the presence of oxygen to form toxic oxygen species that interfere with pathogens’ cellular components and damage them (Konopka and Goslinski 2007). Integration between these technologies and natural products has been showing promising results for oral health (Lima et al. 2024; Ravikumar et al., 2024). Many herbal extracts have shown efficacy against periodontal pathogens. The efficacy of these herbal extracts is summarized in Table 1.

**Table 1:** Efficacy of herbal remedies against periodontal pathogens

Plant name/plant compounds	Part used/type of the extract/dosage form	Method	Results	Ref
*Azadiractha indica* A.Juss *Psidium guajava* L. *Eucalyptus hybrid* (*E. canaldulensis* × *E. ovate*) *Acacia nilotica* L. *Murraya koenigii* L.S *Rosa rubiginosa* L. *Hibiscus sabdariffa* L. *Mangifera indica* L. *Ocimum sanctum* L. *Aloe vera barbadensis* L.	Branches/ethanol	Ethno-pharmacological study Agar well diffusion assay of three primary plaque colonizers, viz., *Streptococcus mutans*, *Streptococcus sanguis*, and *Streptococcus salivarius*	*A. nilotica* extracts produced the largest mean diameter of the inhibition zone (n (21.83 mm ± 0.41) against *S. mutans*, followed in descending order by *P. guajava* (21.17 ± 1.08), *Eucalyptus hybrid* (20.83 ± 1.08), and *M. koenigii* extracts (9.75 ± 1.17), *H. sabdariffa* (7.42 ± 0.20), and *A. indica* (7.25 ± 0.28) *vs* chlorhexidine (positive control) (14.25 ± 0.52) *A. nilotica* (21.33 ± 0.26), *Eucalyptus* hybrid (19.17 ± 0.82), *P. guajava* (18.58 ± 0.59), *M. koenigii* (10.42 ± 0.92), and *H. sabdariffa* (6.50 ± 0.63) inhibited the growth of *S. sanguis* vs. chlorhexidine (21.83 ± 0.61). The others failed to inhibit the growth of bacteria *P. Guajava* (23.00 ± 0.48) produced the highest zone of inhibition against *S. salivarius*, followed by *A. nilotica* (21.17 ± 1.1), Eucalyptus hybrid (20.00 ± 0.45), *M. koenigii* (13.25 ± 0.69), and *H. sabdariffa* (9.75 ± 0.27) vs. chlorhexidine (22.08 ± 0.38). The others failed to inhibit the growth of bacteria	(Chandrashekar et al. 2014)
*Acacia nilotica* L. *Murraya koenigii* Linn. Sprengel *Eucalyptus hybrid* L. *Psidium guajava* L.	Branches/ethanol	Ethno-pharmacological study Broth dilution method A short-term parallel double-blind randomized controlled trial and salivary parameters though salivary flow for pH estimate and microbial assay by spread plate technique	MICs of the polyherbal remedies on *Streptococcus mutans*, *Streptococcus sanguis*, *Streptococcus salivarius*, *Lactobacillus acidophilus*, *Fusobacterium nucleatum,* and *Porphyromonas gingivalis* were found to be 0.25%, 0.05%, 0.05%, 0.1%, 0.25%, and 0.25%, respectively. There was no statistically significant difference between the groups receiving chlorhexidine and polyherbal remedies In vivo, no mortality rates were observed, where mean plaque scores, *S. mutans* colony counts, and total viable counts were highest in placebo group-1 compared to polyhedral mouth rinse group-2 and 0.2% chlorhexidine (p < 0.001). No significant difference in the mean salivary flow rate and pH between different groups	(Chandrashekar et al. 2019)
*Acacia nilotica* L.	Whole plant/(methanol, acetone and aqueous)	Ethno-pharmacological study Well diffusion method Cytotoxicity assay (MTT)	The methanol extract of *A. nilotica* showed significantly higher antimicrobial activity in terms of zone of inhibition (18.00 ± 1.00mm, 20.00 ± 1.15mm and 16.67 ± 0.67mm) and MIC (0.3125, 0.3125 and 0.15625 mg/ml), followed by acetone and aqueous extracts against *Streptococcus mutans, Streptococcus mitis,* and *Prevotella intermedia*, respectively	(Arshad et al. 2017)
*Acer tegmentosum* Maxim. (ATM)	Bark/50% ethanol	Ethno-pharmacological study In vitro, serial dilutions In vivo*,* Ca9-22 and HGF-1 treated with 100 ng/mL *P. gingivalis*-derived lipopolysaccharide (LPS)	The ATM extract demonstrated antibacterial properties against strains of *Porphyromonas gingivalis,* with significant sensitivity at 512 μg/mL, a mean optical density decreasing from 0.80 to 0.033 (p < 0.005) at 500 μg/mL of the ATM solution. MIC was found to be 64 μg/mL The extract showed antioxidative, and anti-inflammatory properties in Ca9-22 and HGF-1 treated with 100 ng/mL *P. gingivalis-*derived lipopolysaccharide (LPS) in vitro and in vivo (ligature-induced periodontitis)	(Choi and Hyun 2020)
*Achillea ligustica* All.	Flowers, leaves, and arial parts/essential oils	Microdilution method	The extract demonstrated inhibitory effects on several oral microorganisms, specifically targeting *Bacillus cereus, Streptococcus pyogenes*, and *Candida albicans* Its efficacy was comparable to that of clove oil. The effectiveness increased when the extract was used in conjunction with Listerine®	(Cecchini et al. 2012)
*Achyranthes aspera* L.	Fresh stems and roots/aqueous extract	Cup plate method to measure the zones of inhibition	The study found that the lowest concentration of the extract inhibited *Streptococcus mutans* growth at 2.5% for both stem and root extracts. The minimum and maximum zones of inhibition were 14 mm, 12 mm, and 16 mm, respectively. The mean zone of inhibition was 13 mm at the lowest volume and 14.7 mm at the highest volume	(Yadav et al. 2016)
*Acmella paniculata* (Wall. ex DC.) R.K.Jansen	Leaves/hexane and methanol Flowers/hexane and dichloromethane	Time-kill assay	Hexane and dichloromethane flower extracts showed antibacterial activity and killed > 3 log_10_ CFU/mL of *Streptococcus mutans* after 24 h at MICs = 12.5 mg/mL and 50 mg/mL, respectively Hexadecenoic and oleic acids were the major compounds for the two extracts, respectively	(Abd Ghafar et al. 2022)
*Aloe vera* L.	Toothpaste contains *Aloe vera* and sodium chloride	Disk diffusion method Biofilm susceptibility test	The antimicrobial effect of the toothpaste on the planktonic form of *Porphylomonas gingivalis* was lower than 0.12% chlorhexidine, but its inhibition effect on the biofilm formation was greater than that of chlorhexidine group	(Vajrabhaya et al. 2022)
*Aloe vera* L.	Toothpaste contains *Aloe vera*	A double-blind prospective randomized trial Gingival and plaque scores were measured both at baseline and 30 days following the intervention	After 30 days, the aloe vera-containing toothpaste group significantly reduced the plaque index scores, and gingival inflammation as compared to the triclosan group At the conclusion of the 30-day follow-up, the aloe vera group had significantly lower total candidal counts and *C. albicans* counts than the triclosan group (p < 0.05)	(Khatri et al. 2017)
*Aloe vera* L.	Fresh leaves/solid mucilaginous gel (AVG)	Disc diffusion and broth microdilution methods	AVG showed antibacterial property against *Actinobacillus actinomycetemcomitans*, *Clostridium bacilli*, *Streptococcus mutans,* and *Staphylococcus aureus*	(Jain et al. 2016)
*Schinus terebinthifolius* Raddi *Psidium guajava* L. *Chenopodium ambrosioides* L. *Punica granatum* L.	Bark Leaves Leaves Leaves/ethanol 70%	Ethnopharmacological survey, agar diffusion method	Participants in the study included 271 individuals, of whom 55.7% reported using plants for medicinal purposes and 29.5% were aware of or used plants for oral diseases. *Aloe vera L., Anacardium occidentale L., Schinus terebinthifolius Raddi, Chenopodium ambrosioides L.,* and *Punica granatum L*. were the most frequently cited of the 34 species that were reported, representing 24 botanical families. Gum bleeding, inflammation, and toothache were the most frequently reported symptoms, followed by the healing process following tooth extraction. *P. guajava L., Schinus terebinthifolius Raddi,* and *Punica granatum L*. all exhibited comparable activity to 0.12% chlorhexidine, which was utilized as a positive control, according to MIC	(Vieira et al. 2014)
Alum Green tea Garlic	Essential oils	Saliva samples taken from children with severe early childhood caries were used to isolate the three microbes The agar diffusion method was used to determine the zone of minimum inhibition	The most effective mouthwash against *Streptococcus mutans* and lactobacilli, according to the study, was chlorhexidine, which was followed by sodium fluoride, fluoride with essential oils, alum, green tea, and garlic with lime. But the best mouthwash to combat *Candida albicans* was garlic and lime Green tea, alum, sodium fluoride, and fluoride with essential oils were all considerably less effective than garlic and lime mouthwash after chlorhexidine	(Thomas et al. 2015)
Anacardiaceae (*Rhus vulgaris* Meikle)	Stem bark/methanol	Microdilution assay	The extract demonstrated significant antimicrobial activity against MRSA, with no cytotoxicity at the highest concentration However, it caused mild irritation with erythema and flaking, which cleared within 8 days No adverse effects were observed from oral administration at concentrations of 50 mg/kg, 300 mg/kg, and 2000 mg/kg	(Mutuku et al. 2020)
*Anacardium occidentale* (cashew) L. *and Mangifera indica* L. (mango)	Leaves/ethanol extracts	The agar plate method; *Enterococcus faecalis*, *Staphylococcus aureus*, *Streptococcus mutans*, *Escherichia coli*, and *Candida albicans* Chinese hamster lung fibroblast (V79) and human gingival fibroblast cell lines	When compared to mouthwashes based on povidone-iodine, cashew and mango leaf extract significantly (p < 0.05) produced a larger zone of inhibition against test pathogens The biofilms of oral pathogens were significantly (p < 0.001) suppressed by plant extracts, even though the mouth rinses' MIC and MBC/MFC values were effective at lower concentrations The cytotoxicity of the leaf extracts was lower than that of mouthwashes (p < 0.001)	(Anand et al. 2015)
*Antrodia camphorata*	Water, 50% ethanol, 95% ethanol, ethyl acetate and chloroform	Adhesion inhibition assay Cytotoxicity on human gingival fibroblast (HGF) cells	The extracts with the lowest MICs against *P. gingivalis* and *S. mutans* were ethanol, ethyl acetate, and chloroform (MIC = 4–16 µg/mL) The aqueous extract's MIC against *S. mutans* and *Porphyromonas gingivalis* was over 2048µg/mL The addition of either the ethyl acetate extract or the chloroform extract (MIC = 16–24 µg/mL) significantly inhibited the in vitro adherence of *S. mutans*, whereas the ethanol extract (MIC = 32–64 µg/mL) showed moderate inhibitory activity. According to the study's findings, *A. camphorata* extracts in ethyl acetate and chloroform might make excellent oral hygiene products to prevent dental cavities and periodontal diseases	(Lien et al. 2014)
Apple	Apple bark (phloretin, dihydrochalcone flavonoid)	In vitro and in vivo of oral candidiasis	Antifungal activity: inhibited biofilm formation and suppressed the yeast-to-hyphae transition Downregulated hypha-associated genes, including enhanced adherence to polystyrene 1 Extent of cell elongation gene 1 Hyphal wall protein 1 gene Agglutinin-like sequence gene 3 Protease and phospholipase secretion: Reduced the secretion of proteases and phospholipases Downregulated expression of protease-encoding genes SAP1 and SAP2, and phospholipase B1 In vivo efficacy: phloretin treatment led to: Reversal of increased lesion severity and inflammatory infiltration in tongue tissues Reduced colony-forming unit (CFU) counts caused by *Candida albicans*	(Liu et al. 2021)
Apple	Antioxidant‐rich apple concentrate (ARAC)	A caries model was developed using *Strptococcus mutans* UA159 biofilms on enamel slabs. Slabs were exposed to sucrose and ARAC daily, and acidogenicity was assessed. Biofilms were extracted to measure polysaccharides, biomass, and bacteria, and demineralization was estimated using slabs	Following a cariogenic challenge with sucrose, *Streptococcus mutans* biofilms were exposed to ARAC, which resulted in less enamel demineralization than the positive control. The greatest demineralization reduction of roughly 57% was observed at the highest ARAC dilution of 1:100,000 (*v/v*). The highest dilution of the apple concentrate caused the biofilm to produce significantly less extracellular polysaccharide, even though there were no differences in the number of bacterial cells, intracellular polysaccharides, or biomass (p > 0·05)	(Giacaman et al. 2014)
Apple cultivar (Jinshiji)	Thinned-young apple polyphenols such as phlorizin, chlorogenic acid, and ( −)-epicatechin, among others	Microdilution method Scanning electron microscopy (SEM) Confocal laser scanning microscopy (CLSM)	The extract decreased the viability (< 50%) of halitosis-causing bacteria: *Fusobacterium nucleatum*, *Porphyromonas gingivalis*, and *Prevotella intermedia* at MICs = 10.00 mg/mL, 8.00 mg/mL, and 8.00 mg/mL, respectively Scanning electron microscopy and confocal laser scanning microscopy showed that the extract could cause morphological changes of bacterial cells and induce apoptosis	(Liu et al. 2022)
*Arctium lappa* L. (burdock)	Propylene glycol	Broth microdilution method Anti-biofilm activity MTT assay was used to assess the cytotoxicity on RAW 264.7, and ELISA to quantify IL-1β and TNF-α	The biofilms of *Staphylococcus aureus* (0.438 ± 0.269), *S. epidermidis* (0.377 ± 0.298), *S. mutans* (0.244 ± 0.161), and *Candida albicans* (0.746 ± 0.209) were significantly reduced (log10) at the most effective concentration, 250 mg/mL The viability of the cells was almost 100%. TNF-α was inhibited (p < 0.01) and IL-1β production was comparable to the control group (p > 0.05)	(de Oliveira et al. 2014)
*Artemisia herba alba* L. *Centaurium erythraea* L. *Juglans regia Laurus nobilis* L. *Matricaria recutita* L. *Mentha pulegium* L. *Mentha piperita* L. *Taraxacum officinale* L. *Origanum vulgare* L.	Leaves/ethanol and water	Agar disc diffusion and microdilution methods	Ethanolic extracts were potent than aqueous extracts against a combination of *Streptococcus, Enterococcus,* and *Lacticaseibacillus* isolates *Origanum vulgare leaves ethanolic* extract (with thymol is the main constituent) showed the highest antibacterial activity with MICs ranging from (1.56 to 25.0 mg/mL) against different isolates It also showed anti-biofilm formation, a reduction in acidogenesis, inhibition of glucosyltransferase activity, and a downregulation in the expression of multiple virulence-associated genes	(Idir et al. 2022)
*Avicennia marina*	Leaves aqueous extract/zinc oxide nanoparticles	Disc diffusion method	At 100 µg/mL, the nanoparticles showed antibacterial activity against *Streptococcus mutans*, *Staphylococcus aureus*, and *Klebsiella* sp. with zones of inhibition 7.5 ± 0.2, 9.5 ± 0.5, and 9.5 ± 1.2, respectively At 75 µg/mL, the inhibition zones were 7 ± 0.25, 9 ± 1, and 7.5 ± 0.5 mm, respectively	(Tamanna et al. 2023)
*Azadirachta indica* L. *Pongamia pinnata* L. *Psidium guajava* L. *Mangifera indica* L.	Leaves/ethanol	Synergistic antibacterial activity	The antibacterial activity of *M. indica* and *A. indica* was maximum at doses of 0.3 mg/mL and 6.25 mg/m, respectively *M. indica* outperformed the positive control quercetin in superoxide scavenging potential, while *A. indica* had the greatest free radical scavenging of DPPH, showing 50% inhibition at 28.72 µg/mL *M. indica* and *A. indica* exhibited acceptable efficacy against the nitric oxide free radical, with respective concentrations of 12.87 and 18.89 µg/ml The GTFB gene's expression was preferentially downregulated by *M. indica*, suggesting a mechanism involving glucose tranferases that target bacterial adhesion in particular	(Bodiba et al. 2018)
Bamboo	Leaf extract solution (BLES)	Antibacterial action was examined in oral isolates of *Porphyromonas gingivalis* W83, *Prevotella intermidai* TDC19B, *Fusobacterium nucleatum* ATCC25586, and *Prevotella nigrescence* ATCC33563	BLES inhibited bacterial proliferation at concentrations varying from 0.16% to 0.25%, with no viable bacterial colony observed at the original concentration. Strain growth was eliminated at lower concentrations	(Majbauddin et al. 2015)
Bamboo salt	Herbal toothpaste	Randomized double-blinded controlled clinical trial	The herbal toothpaste successfully reduced salivary *Streptococcus mutans* and *Lactobacillus* after 4 weeks and the results were comparable with that of non-herbal conventional toothpaste	(Biria et al. 2022)
Berries Tea plant	Polyphenols	Supragingival plaque from 16 children aged 7–11 years was suspended in TSB for testing In vitro growth and biofilm formation of plaque bacteria	Inhibition growth and biofilm formation of children’s plaque bacteria	(Wu et al. 2021)
Black tea	Theaflavins (TFs)	Oral microbiome analysis from saliva and supragingival plaque of 20 healthy adults by 16S rRNA gene sequencing	Alpha and beta diversity analysis showed that tooth brushing with toothpaste containing TFs increased microbial abundance in saliva samples and altered oral microbiota It reduced oral pathogens such as *Prevotella, Selenomonas*, and *Atopobium*, while increasing oral health-associated bacteria like *Streptococcus* and *Rothia* Additionally, toothpaste with TFs reduced functional pathways related to exopolysaccharide (EPS) synthesis, enriching functions in transporters, ABC transporters, two-component systems, and amino acid metabolism	(Kong et al. 2021)
Black tea	Leaves/chloroform and ethyl acetate	Serial dilution method	Against the microorganisms studied, black tea extract showed adequate antibacterial activity Black tea extract had the lowest MIC against *Staphylococcus aureus*	(Goswami et al. 2020)
Black tea	Leaves/aqueous	Microdilution assay Cultivation of oral epithelial cells and determination of cytotoxicity	Antibacterial activity was reflected against major periodontopathogens (*Porphyromonas gingivalis)* as well as attenuating the secretion of IL-8 and inducing hBD secretion in oral epithelial cells	(Lombardo Bedran et al. 2015)
*Brucea javanica* L. *Piper beetle* L.	Seeds Leaves/aqueous extract	In the former, the hydrophobic interaction of the candida cells was measured by adsorption to hexadecane In the latter, the experimental pellicles in the particular adhesion of oral candida to the surface of hard tissue were represented by glass beads coated with saliva	*Candida krusei, C. dubliniensis,* and *C. tropicalis* showed the highest adsorption to hexadecane, with the remaining species falling between 7 and 10% *B. javanica* showed a 60% decrease in CSH compared to *Piper betle*, while *C. parapsilosis* exhibited the highest specific-binding affinity to pellicle	(Harun and Razak 2013)
*Brucea javanica L.*	Seeds/aqueous extract	Antifungal activity The optical density was calculated	The extract from *B. javanica* seeds showed antifungal properties, with the highest growth rate for *Candida tropicalis*. The extract significantly reduced μ-values of *C. dubliniensis, C. krusei,* and *C. parapsilosis*, and prolonged g-values in most *candidal strains*. The average number of CFU/ml in the candidal population decreased	(Nordin et al. 2013)
Cacao Shrimp *Aloe vera* Miswak tree Propolis Theobromine Chitosan	Ethanolic extracts	Agar well diffusion	Toothpastes containing theobromine and chitosan, as well as traditional toothpaste, and demonstrated antimicrobial efficacy against all tested bacteria In contrast, toothpastes with *A. vera*, miswak, and propolis were effective only against *Streptococcus mutans*, while those containing probiotics and enzymes showed no antimicrobial effects Among the natural ingredient toothpastes, the one with theobromine exhibited the highest efficacy against *S. mutans*, whereas the toothpastes with *A. vera* and propolis showed the lowest efficacy (p < 0.05)	(Demir et al. 2021)
Calendula	Essential oil	Posaconazole, calendula oil, and chitosan essential oil-based nanoemulsions were prepared using a response-surface Box–Behnken design The doses of posaconazole (10, 15, and 20 mg), the percentages of calendula oil (6%, 12%, and 18%), and the percentages of chitosan (0.5%, 1.5%, and 2.5%) varied	The optimized formulation resulted in a 22-mm bacterial growth suppression zone, and 25-mm fungal growth inhibition zone. The formulation included 20 mg of posaconazole, 18% calendula oil, and 1.35% chitosan was created using the proper design. When compared to previous formulations, this improved formulation significantly reduced the ulcer index in rats. Consequently, this study demonstrated that posaconazole, calendula oil, and chitosan essential oil-based nanoemulsions could offer effective defense against gingivitis caused by microbes	(Alissa et al. 2024)
*Camellia japonica* L. *Thuja orientalis* L.	Leaf/methanol extracts	Microdilution assay Determination of GTase inhibition activity	Eight plant extracts from 37 herbs, including *Camellia japonica* and *Thuja orientalis*, showed significant inhibitory effects on etiologic bacteria growth and biofilm formation. These extracts reduced bacteria growth by over 76% and 83%, and inhibited biofilm formation by over 92.4% and 98.0%, respectively	(Choi et al. 2017)
*Camellia sinensis* L. *Zanthozylum limonella* (Dennst.) Alston *Acorus calamus* L.	Leaves Fruits Stems and leaves/95% ethanol	Agar well diffusion method (gentamicin was used as standard)	Although *Acorus calamus* does not have the highest content of phenolic acids and flavonoids, it showed superior activity against *streptococcus mutans* in terms of MIC (0.3125%) Oral ulcer gel and oral spray from these plants displayed antibacterial activity and patients’ satisfaction	(Chaiwaree et al. 2023)
Cannabinoids	-	A randomized controlled trial, comparison of growth media and assay methods using minimum inhibitory concentration	Alcohol- and fluoride-free CannIBite mouthwash products with a cannabis infusion provide a safer and more effective alternative Mouthwashes infused with cannabinoids (CBD/CBG) and other essential natural constituents exhibit encouraging bactericidal action in vitro against the total culturable aerobic bacterial content in dental plaque, matching or surpassing the efficacy of the industry standard (0.2% chlorhexidine)	(Vasudevan and Stahl 2020)
*Centella asiatica* L. *Polygonum cuspidatum* root Houtt *Scutellaria baicalensis* root Georgi *Camellia sinensis* leaf L. *Glycyrrhiza glabra* root L. *Chamomilla recutita* flower L. *Rosemary rosmarinus officinalis* leaf L. *Salvia officinalis* L.	Toothpaste contains this herbal mixture and fluoride	Time-dependent killing assay (chlorhexidine was used as standard)	The herbal toothpaste showed superior inhibitory and bactericidal effects against *Streptococcus mutans*, *Streptococcus sanguinis,* and *Porphyromonas gingivalis* than non-herbal toothpaste The herbal toothpaste also inhibited interleukin-1 β and interleukin-6 production in human gingival epithelial cell	(Qi et al. 2022)
*Chamaecyparis taiwanensis* Masam. & Suzuki	Hinokitiol in 0.2% DMSO	Agar and broth dilution methods	Hinokitiol has demonstrated antimicrobial properties, with MICs ranging from 40 to 110 μM and MMCs from 50 to 130 μM, effectively acting against MRSA, *Aggregatibacter actinomycetemcomitan, Streptococcus mutans,* and *Candida albicans* A positive PIP was detected in *A. actinomycetemcomitan, S. mutans,* and *MRSA*, which was linked to low autolysin activity Additionally, hinokitiol exhibited notable antimicrobial activity and cytotoxicity at 200 μM against oral pathogens and oral squamous cell carcinoma cell lines Importantly, it displayed reduced cytotoxicity toward normal human oral keratinocytes, indicating its potential as a safe agent for oral healthcare applications	(Shih et al. 2013)
*Chelidonium majus* L.	80% methanol, *n*-hexane, ethyl acetate, *n*-butanol, and water fractions	The paper disc agar plate and broth microdilution methods	The 80% methanol, ethyl acetate, and butanol fractions inhibited *S. mutans*, *Streptococcus gordonii*, *Streptococcus sobrinus*, and *Streptococcus sanguinis*, but the water extract was totally ineffective The ethyl acetate fraction showed the highest antimicrobial activity against *S. gordonii*, *S. sobrinus*, and *S. sanguinis* The butanol fraction was the most effective against *S. mutans,* and the hexane fraction was only effective against *S. gordonii* -The ethyl acetate MICs against *S. mutans*, *S. gordonii*, *S. sobrinus*, and *S. sanguinis *were 125, 250, 125, and 250 µg/mL, respectively	(Hong et al. 2024)
Cinnamic acid	Zinc oxide nanoparticles capped with cinnamic acid	Zone inhibition and microplate assays AutoDock was used to study cinnamic acid interactions with microbes’ receptors	The nanoparticles inhibited the growth of *Staphylococcus aureus*, *Streptococcus mutans*, *Enterococcus faecalis*, and *Candida albicans* at MIC of 25 μg/mL The 50 μg/mL results were more comparable to that of amoxicillin (50 µg/ mL) Cinnamic acid interfered with *S. aureus* surface protein G, *S. mutans* PTSIIA, *Enterococcal* surface protein, and *C. albicans N*-myristoyltransferase through interaction with a number of amino acids	(Ravikumar et al., 2024)
Cinnamon	Cinnamaldehyde	Anti-biofilm activity using isothermal microcalorimetry	The tested material exhibited antimicrobial properties against *Streptococcus epidermidis*, *Streptococcus mutans*, and *Streptococcus mitis* clinical isolates The strongest inhibition was observed against *S. epidermidis*, with a 70% reduction in growth rate and a 12-h extension of the lag phase For *S. mutans* and *S. mitis*, the growth rate decreased by 20% and 10%, respectively, with lag phase increases of 2 h and 6 h	(Worreth et al. 2022)
Cinnamon	Cinnamon oil	Rheological characterization, in vitro release studies, and latency reaction time	The optimized cinnamon oil-loaded nanoemulsion gel (CO–NEG) formulation showed pseudoplastic behavior and enhanced eugenol release compared to pure CO It also showed the highest ex vivo mucosal permeation The latency reaction time was significantly prolonged, outperforming other formulations This suggests that the CO–NEG formulation can improve oral health by enhancing cinnamon oil's actions against oral microbiota	(Hosny et al. 2021)
Cinnamon Clove Bergamot Orange	Essential oils	Saliva tests	The study demonstrated how the four essential oils work in concert or antagonistically to prevent the growth of *Streptococcus mutans* Saliva test demonstrated the synergistic effect of the active ingredients of essential oils evaluated from tertiary emulsions, which inhibit *S. mutans*' ability to grow in oral cavities	(Alexa et al. 2019)
*Cistus* × *incanus* L. and *Scutellaria lateriflora* L.	Hydroalcoholic extract of *C. incanus* (standardized to contain ≥ 18% of total polyphenols, and arabic gum as a carrier agent). Hydroalcoholic extract of *S. lateriflora* (standardized to contain ≥ 10% of baicalin, and maize maltodextrin as carrier agent) obtained from the aerial parts of the plants	Agar diffusion method Colony-forming unit (CFU) count	The polyphenols of the two extracts were stable after simulated digestion and showed mild and dose-dependent antibacterial activity against *Porphyromonas gingivalis* The activity was boosted by combining both extracts	(Ullah et al. 2023)
Citrox® formulations	Bioflavonoid extract from citrus fruits	Broth microdilution assay Modified microtitre biofilm assay	Both formulations of Citrox® (BC30 and MDC30) exhibited antimicrobial properties. Notably, BC30 demonstrated superior efficacy by significantly reducing the growth of all tested bacterial species and the majority of *Candida* species at a concentration of 1% (*v/v*)	(Hooper et al. 2011)
*Citrus aurantifolia* L. *Berberis vulgaris* L. *Citrus citratus* L. *Cinnamon zeylanicum* L.	Essential oils	Anti-biofilm activity	Essential oils exhibit significant antibacterial activity against cariogenic strains of *Streptococcus mutans*, effectively inhibiting biofilm formation at low concentrations. Their incorporation into products like Listerine® highlights their efficacy in combating *S. mutans* biofilms The antibacterial properties of these essential oils stem from their ability to penetrate bacterial cell membranes, altering fatty acid profiles and increasing permeability, which leads to the release of cellular contents Additionally, once inside the cell, these compounds target and disrupt organelles, further contributing to their antimicrobial effects	(Lugo-Flores et al. 2021)
Coffee and chlorogenic acid	Aqueous extract of coffee	Disc diffusion, turbid metric, and plate count assays	MICs were 16 and 4 mg/mL, for coffee and chlorogenic acid, respectively, using disc diffusion test In addition to its antibacterial properties, chlorogenic acid also inhibited *Porphyromonas gingivalis*'s protease activity Moreover, coffee extract prevents *P. gingivalis* from proliferating, a phenomenon that may be partially explained by the action of chlorogenic acid	(Tsou et al. 2019)
Commercial green tea, oolong tea, black tea, pu-erh tea and chrysanthemum tea	90% acetone extract	Bacterial attachment to human gingival fibroblast cells through attachment assays after incubation and quantification by serial dilution	Pu-erh tea and chrysanthemum tea extracts have shown a strong ability to prevent the attachment of harmful oral bacteria, such *as Streptococcus mutans*, to gum cells. This suggests that these extracts may have the potential to improve the health of oral soft tissues	(Wang et al. 2013)
*Corchorus olitorius* L. *Acmella caulirhiza* Rich.	Leaves Aerial parts/diethyl ether, methanol, and water extracts	Agar well diffusion (ciprofloxacin was used as standard)	The aqueous extract of *C. olitorius* and diethylether extract of *A. caulirhiza* showed the highest zone of inhibitions against *Streptococcus mutans* (16.10 mm and 12.03 mm, respectively) at 1000 mg/ml Although the diethyl ether extract of *A. caulirhiza* showed the least MIC of the same plant extracts, the MIC of diethyl ether extract of *C. olitorius* was lower than the MIC of the aqueous extract	(Namwase et al. 2021)
Cranberry	Cranberry juice and cranberry beverage, both are rich in phenolics	Serial dilutions pour plate method Electrical impedance change measurements	The beneficial *Lactobacillus paracasei subsp. paracasei* was the least vulnerable strain to cranberry beverage Cranberry juice inhibitory activity against *Actinomyces naeslundii* was higher than their activity against *Streptococcus mutans*, but cranberry beverage showed the opposite	(Nowaczyk et al. 2021)
Cranberry	Deacidified cranberry juice	Electrodialysis with bipolar membrane (EDBM) for the deacidification of cranberry juice	Cranberry juice reduced the bactericidal effects against *Aggregatibacter actinomycetemcomitans* and *Porphyromonas gingivalis*, but not *Fusobacterium nucleatum*	(Pellerin et al. 2021)
*Crataegi fructus* Gand	Berry70% ethanolic extract	Survival rate of human keratinocytes was addressed using water-soluble tetrazolium salt (WST-1) analysis	*Crataegi fructus showed* antimicrobial activity against *Streptococcus mutans* (MIC = 30 mg/mL) and *Candida albicans* (MIC = 10 mg/mL) in a concentration-dependent manner 30 mg/mL demonstrated optimal activity without affecting cell proliferation	(Nam 2024)
*Crocus sativus* L.	Ethanolic extract	Biofilm formation assay	*C. sativus* in 60 μg/ml, 30 μg/ml and 8 μg/ml showed strong, moderate, and weak anti-biofilm activity in *Streptococcus mutans* clinical isolates, respectively	(Jalili et al. 2021)
*Curcuma aeruginosa* Roxb. *C. mangga* Val. *C. xanthorrhiza* Roxb. *Kaempferia galanga* L.	Essential oil with ethyl cinnamate is the major constituent in *C. xanthorrhiza* and *β*-myrcene, the major one in *C. mangga*	Kirby–Bauer disc diffusion susceptibility test (Oradex, a chlorhexidine-based mouthwash, was used as standard)	*C. xanthorrhiza* oil was the most potent against *Streptococcus mitis* (19.50** ± **2.22 mm zone of inhibition) and *Streptococcus sanguinis* (15.04** ± **3.05 mm) *C. mangga* oil showed the highest activity against *Streptococcus mutans* (12.55** ± **0.45 mm) and mixed oral bacteria (15.03** ± **3.82 mm)	(Amil et al. 2024)0
*Curcuma longa* L.	Rhizomes/aqueous extract (CRU)	Antifungal susceptibility testing	Curcumin adsorption reduced *Candida albicans* adhesion. Moreover, curcumin prevented biofilm formation and promoted *Candida albicans* aggregation. Furthermore, Curcumin affected the temporal expression of *Candida albicans* adhesins	(Alalwan et al. 2017)
*Curcuma zanthorrhiza* Roxb	Essential oil	Antimicrobial against *Streptococcus mutans* biofilms	*Curcuma xanthorrhiza* oil (Xan) nanoemulsion demonstrated stable and strong antimicrobial effects, indicating their potential use in oral health treatment	(Cho et al. 2020)
Curcumin	Curcumin mediated zinc oxide nanoparticles	Zone inhibition and microplate assays AutoDock simulation	The nanoparticles inhibited the growth of *Staphylococcus aureus*, *Streptococcus mutans*, *Enterococcus faecalis*, and *Candida albicans* at MIC 40 µg/mL The 80 µg/mL results were more comparable to that of amoxicillin (50 µg/ mL) Curcumin interfered with *S. aureus* surface protein G, *S. mutans* Antigen I/II carboxy-terminus, *Enterococcal* surface protein, and *C. albicans* ALS3 through binding with different groups of amino acids	(Tayyeb et al. 2024)
*Cynodon dactylon* L.	3,7,11,15 tetramethylhexadec-2-4dien 1-o1, 3,7,11,15 tetramethylhexadec-2-en-1-o1, and stigmasterol compounds	A microplate system was used to produce the biofilms	3,7,11,15-tetramethylhexadec-2-en-1-ol (from phytol derivatives) (MIC = 12 µg/mL) displayed higher anti-biofilm activities on *S. mutans* than the other 2 compounds	(Habib et al. 2023)
*Cyperus articulatus* L. (priprioca)	Rhizomes and solid waste ethanolic extracts with oxygenated sesquiterpenes are the predominant class and mustakone the main compound	Broth microdilution method (chlorhexidine was used as standard)	The rhizomes’ ethanolic extract was more potent than solid waste extract. It inhibited *Streptococcus mutans* with MIC = 0.29 mg/mL and *Enterococcus faecalis* with MIC = 1.17 mg/mL	(Macambira et al. 2024)
Essential oils	-	Commercial mouthwashes containing essential oils (EO) or chlorhexidine (CHX) as the active ingredient were used to treat human gingival and periodontal ligament (PDL) fibroblasts The ability of each mouthwash to influence fibroblast migration and survival as well as long-term impacts on cell viability was examined at a variety of concentrations	Undiluted mouthwashes caused cell death 24 h after treatment, with CHX and EO mouthwashes causing 50% cell death at varying dilutions. EO did not decrease migration at 10% concentrations. EO treatment did not cause gingival fibroblast death	(Tsourounakis et al. 2013)
*Etlingera pavieana* (Pierre ex Gagnep.) R.M.Sm	Essential oil of the rhizome in comparison with the main constituents (methyl chavicol and *trans*-anethol)	Disk diffusion and broth microdilution methods (chlorhexidine was used as standard)	The MIC against *Streptococcus mutans and Streptococcus sobrinus* of the oil and *trans*-anethol was > 1.6% v/v, while the MIC of the methyl chavicol was 0.4% v/v The oil and *trans*-anethol showed anti-biofilm against *S. sobrinus* more than *S. mutans,* while methyl chavicol exhibited almost equal anti-biofilm activity against the two species	(Wongsariya et al. 2024)
*Eugenia uniflora* L.	3% Hydroalcoholic extract of the ripe fruit	Randomized clinical trial with 40 dental students (21–24 years), divided into two groups: Group 1 (*Eugenia uniflora* L. dentifrice), and Group 2 (Colgate Total 12®) Indicators measured: OHI-S, GBI, and salivary *S. mutans* count (cfu/mL) over 22 days	Both groups exhibited a statistically significant reduction in oral hygiene index-simplified (OHI-S), gingival bleeding index (GBI), and colony-forming units per milliliter (CFU/mL), with p values less than 0.01 Notably, the *Eugenia uniflora* L. dentifrice demonstrated comparable effectiveness to Colgate Total 12®, showing a significant distinction solely in the GBI results (p < 0.01)	(de Carvalho JOVITO et al., 2009)
Gambir (*Uncaria gambir* (W.Hunter) Roxb.)	Catechin from this plant	In silico study using AutoDock	The catechin showed comparable binding affinity and similar amino acid attachment as chlorhexidine with MurB enzyme of *streptococcus mutans* that contribute in peptidoglycan synthesis of this bacterium	(Dharsono et al. 2022)
*Garcinia kola* Heckel, Nigerian chewing stick	Methanol extract, antibacterial biflavonoid, GB1	Standard microbiological assays	GB1 has a bactericidal effect on *Streptococcus mutans* and other oral bacteria, with MICs ranging from 32 to 64 μg/ml. Moreover, at a concentration of 256 μg/ml, GB1 exhibited antibacterial properties. Prevents glucose absorption and production of insoluble glucan. This indicates that metabolic alteration may occur in the antibacterial mechanism	(Xu et al. 2013)
Green tea	Commercially available dentifrices Splat Green Tea Toothpaste Curasept containing probiotic and chlorhexidine (CHX)	It is a double‑blinded, parallel group, randomized controlled clinical trial	The mean *Streptococcus mutans* and Lactobacillus colony counts were significantly lower on the 30th day of follow-up, indicating that all groups had demonstrated antimicrobial action CHX dentifrice group, outperformed the other groups in all preventive modalities	(Prabakar et al. 2018)
Green Tea (*Camellia sinensis* L.) *Salvadora persica* L.	Leaves Twigs/branches	A 4-day plaque regrowth randomized crossover trial was conducted with 15 participants. Plaque quantity was evaluated at 24 h and after 4 days using digital plaque image analysis (chlorhexidine 0.2% was used as standard)	A commercial mouthwash (COM) significantly reduced plaque accumulation (31.933 ± 10.025) compared to Plc (54.629 ± 17.555) for a period of 4 days; a comparable effect was ascribed to CHX (34.903 ± 11.871) Moreover, it significantly reduced the amount of Streptococcus sanguinis, *Actinomyces viscosus*, and *Actinomyces naeslundii* (primary colonizers) in saliva Using CoM for 4 days twice daily could reduce plaque accumulation and might be considered as an alternative to synthetic mouthwashes	(Salah et al. 2020)
Gutiferone from red propolis	Photodynamic therapy	Broth microdilution (amphotericin B was used as standard) Quantifying biomass using crystal violet detachment to quantify biomass Total plate count for cell counting In vivo buccal candidiasis model in mice	Gutiferone showed efficacy *against Candida albicans, Candida tropicalis*, and *Candida glabrata* with MIC = 1000 μg/mL Photodynamic therapy using gutiferone particularly affected *C. tropicalis* and reduced biofilms by 3.68Log10 CFU/mL which was confirmed in vivo	(Lima et al. 2024)
Gymnemic acids	Extracted from *G. sylvestre* R. Br. plant leaf by 75% ethanol and vacuum dried	Minimum biofilm inhibition concentrations Mono-species and dual-species biofilm assay Measurement of biofilm Extracellular DNA (eDNA) Semiquantitative RT–PCR Determination of glyceraldehyde-3-phosphate dehydrogenase (GAPDH) activity	Certain biofilm-related genes from both microorganisms revealed altered expression in treated versus control biofilms when subjected to semiquantitative PCR Recombinant *Streptococcus gordonii* GAPDH was shown to be inhibited by GAs For *Streptococcus gordonii*, concentrations > 400 mg/mL and *Candida albicans*, concentrations > 200 mg/mL were observed to significantly prevent the production of biofilms Whereas *S. gordonii* showed the highest biofilm development inhibition (80–90%) between 500 and 600 mg/mL, *C. albicans* showed only a 50% biofilm growth inhibition at that GAs dose	(Veerapandian and Vediyappan 2019)
Hull blackberries (*Rubus eubatus cv. “Hull”*)	Ethanol and 0.01% HCL extract of fruit The extract was fractionated into an anthocyanin-enriched fraction	Colorimetric water-soluble tetrazolium-1 (WST-1) assay to measure metabolic activity	The extracts showed decreased metabolic activity of *Fusobacterium nucleatum, Porphyromonas gingivalis,* and *S. mutans* at concentrations ranging from 350 to 1,400 μg/mL On the other hand, the anthocyanin-rich fraction of BBE specifically inhibited *F. tuberculosis*	(González et al. 2013)
*Juniperus excelsa* M. Bieb.	Essential oil by hydrodistillation	CFU count, quantitative cell viability 2,3-bis(2-methoxy-4-nitro-5-sulfophenyl)-5-[(phenyl amino) carbonyl]-2H-tetrazolium hydroxide assay (XTT), and bacterial inhibition halo analysis were assessed against the two biofilm formers, *Streptococcus mutan*s and *Aggregatibacter actinomycetemcomitans* Human primary gingival fibroblasts (HGF) and mucosal keratinocytes (HK) were used to evaluate cytocompatibility	XTT analysis and CFU counts confirmed that tenfold-diluted essential oil determined a statistically significant (p < 0.05) reduction in bacteria count and viability toward both biofilm and planktonic forms in a manner similar to those obtained with CHX. An inhibition halo test showed that both bacteria were sensitive to the essential oil. Additionally, essential oil was more cytocompatible than CHX (p < 0.05). In summary, essential oil demonstrated a promising antiseptic substitute for CHX due to its superior cytocompatibility and comparable bactericidal activity	(Azzimonti et al. 2015)
Labrador tea (*Rhododendron groenlandicum* [Oeder] Kron & Judd), peppermint (*Mentha* x *piperita* L.), winter savory (*Satureja montana* L.),	Essential oils (EO)	Broth microdilution assay Anti-biofilm activity Luminescence assay VSC production measurement biocompatibility testing	Antibacterial activity: winter savory EO showed significant antibacterial activity against *Fusobacterium nucleatum*, with Labrador tea and peppermint EO exhibiting lesser effects Biofilm viability: treatment with EO significantly decreased the viability of pre-formed biofilms, as indicated by reduced ATP production Membrane permeabilization: the EO were found to permeabilize the bacterial cell membrane, indicating this as a target for the EO's action Reduction of VSC production: all three EO reduced VSC production by *F. nucleatum* in a dose-dependent manner Biocompatibility: no significant loss of cell viability was observed in oral keratinocytes treated with EO at effective concentrations against *F. nucleatum*	(Ben Lagha et al. 2020)
*Laminaria japonica* J.E. Areschoug *Rosmarinus officinalis* L.	Ethanolic extract	Broth dilution method	Using specific concentrations of chlorhexidine digluconate or protamine sulfate in combination with *L. japonica* or *R. officinalis* extracts, except for chlorhexidine digluconate and *L. japonica*, resulted in a synergistic antibacterial effect against *S. mutans*	(Yoo et al. 2020)
*Laminaria japonica* J.E. Areschoug	Ethanol extract	Microdilution method	The extracts had significant antimicrobial activity, with MIC values ​​ranging from 62.5 to 500 µg/mL for various oral streptococci and MBC from 125 to 1000 µg/ml, especially *Actinomyces naeslundii* having an MIC of 250 µg/ml. While *Actinomyces odontolyticus* had an MIC of 62.5 µg/mL, it was shown to be low. The corresponding MBC was 500 µg/mL for *A. neslundi* and 250 µg/ml for *A. odontolyticus*. Additionally, *Fusobacterium nucleatum* and *Porphyromonas gingivalis* had MIC values ​​of 250 µg/ml and 62.5 μg/ml, respectively. The extracts were used with *Streptococcus mutans*, *A. odontolyticus*, *P. gums,* and other pathogens It also changes the texture of the surface. The extract also altered the surface texture of pathogens like *Streptococcus mutans*, *A. odontolyticus*, and *P. gingivalis*, indicating its potential application in antimicrobial dental formulations	(Kim et al. 2013)
*Lentinus edodes* (shiitake mushroom) and *Cichorium intybus* (Italian red chicory)	Aqueous extract, LMM (< 5,000 D)	Evaluation of cell growth and viability (OD measurement, CFU counting) Scanning electron microscopy for morphological analysis	The study revealed a bacteriostatic effect, characterized by a complete halt in DNA synthesis, while RNA synthesis experienced only partial inhibition. Notably, despite these disruptions, protein synthesis persisted, and an observable elongation of cells occurred following the inhibition of septum formation	(Signoretto et al. 2011)
Lichen	Usnic acid (UA)	Disc diffusion, growth kinetics, and biofilm formation assays Gene expression analysis Acidogenicity, acidurity, eDNA synthesis, and response to oxidative stress Toxicity assessment	MIC and MBC: UA exhibited MIC at 5 µg mL⁻^1^ and MBC at 10 µg mL⁻^1^ Biofilm formation: biofilm formation was reduced in a concentration-dependent manner Gene expression: downregulation of GTFB, GTFC, GTFD, VICR, COMDE, and smu0630 was observed Functional effects: UA attenuated acidogenicity, acidurity, eDNA synthesis, and response to oxidative stress Resistance development: very low frequency of spontaneous resistance development in *Streptococcus mutans* was noted Toxicity: no morphological aberrations or toxic effects were observed in human buccal epithelial cells or oral commensals	(Priya et al. 2021a)
Licorice (*Glycyrrhiza glabra* L.)	Ethanol extract containing glycyrrhizol A	Microdilution broth assay	The extract demonstrated significant antimicrobial properties, exhibiting an MIC of less than 40 μg/mL against bacteria responsible for cavities. Additionally, pilot studies involving sugar-free lollipops revealed their effectiveness in diminishing the presence of cariogenic bacteria in human subjects	(Hu et al. 2011)
Lingonberry	Photodynamic treatment with visible light plus water-filtered infrared-A irradiation and natural single- or multi-component photosensitizers	Disc diffusion method	Lingonberry combined with novel techniques displayed antimicrobial activity against *Enterococcus faecalis, Streptococcus mutans, Streptococcus oralis, Streptococcus sobrinus, Veillonella parvula,* and *Fusobacterium nucleatum*	(Klein et al. 2023)
*Lippia alba* (Mill.) *Cymbopogon citratus* L.	Whole plant/essential oil extraction through steam distillation	An assay called MBEC-high-throughput (MBEC-HTP) to measure the amount of *S. mutans* ATCC 35668 strain biofilms that were eradicated CHO cells were subjected to cytotoxicity assessment using the MTT cell proliferation assay	Geraniol and citral were the main constituents of both oils; they were 18.9% and 15.9% in *L. alba* and 31.3% and 26.7% in *C. citratus*, respectively. No concentration of either essential oil was toxic to CHO cells over a 24-h period. The essential oils of *L. alba* and *C. citratus* demonstrated eradication activity against *Streptococcus mutans* biofilms of 95.8% and 95.4%, respectively, at 0.1 and 0.01 mg/dL concentrations and 93.1%, at 0.001 mg/dL concentration	(Tofiño-Rivera et al. 2016)
*Liquidambar styraciflua* L. *Pistacia lentiscus* L. Yarrow *Diospyros virginiana* L. Tamarind *Vicia faba* *Quercus alba*, the white oak *Carya alba* (L.) Nutt. ex Elliott *Juglans regia* *Sassafras albidum* *Azadirachta indica* Jackfruit *Morella cerifera* Olive Yellow dock *Resurrection fern* Orange *Zanthoxylum armatum* Black willow *Sideroxylon celastrinum* *Gum bumelia* Muscadine Common Grape Vine	Woody part Leaf Inflorescence Stem Leaf Flower, leaf, root, stem Bark Woody part Woody stem Leaf Leaf, woody stem Leaf Leaf, flower Leaf Aerial part, fruit, leaf, stem Whole plant Fruit rind Fruit, seed Leaf Stem Leaf Leaf, stem Stem	Agar diffusion assay Cytotoxicity assessment	Out of 109 extracts from 21 selected plant species, 21 extracts from 11 plants exhibited over 90% inhibition of *Porphyromonas gingivalis* at 64 μg/mL Further tests for minimum inhibitory concentration (MIC) revealed: Best MIC: *Pistacia lentiscus* fruits had the lowest MIC at 8 μg/mL Second best MIC: *Zanthoxylum armatum* fruits/seeds showed an MIC of 16 μg/mL *P. lentiscus* fruits also had the highest selectivity index (SI) of 256 Overall activity: most extracts demonstrated promising antibacterial activity with low cytotoxicity The study suggests that further testing for biofilm eradication and activity against other dental pathogens is warranted, and it lays the groundwork for future development of these extracts as ingredients in oral hygiene products	(Carrol et al. 2020)
Magnolia bark extract (MBE)	Gum containing MBE extract is prepared	A single-center, double-blind, single treatment, randomized, placebo-controlled trial	Chewing gum containing MBE (0.4%) and lauramide arginine ethyl ester (LAE) (0.5%), under the above-described regimen, significantly inhibited the development of plaque The absence of a plaque-inhibiting effect in MBE without surfactant (LAE) indicates that the surfactant increased the MBE components' bioavailability	(Komarov et al. 2017)
*Magnolia grandiflora* L.	Bark extract/hydro-ethanol	In a clinical trial study, the prevalence of *Streptococcus mutans* was measured in dental plaque at different stages, with plaque index and saliva sampling conducted during follow-up visits by a dentist. The study was placebo-controlled and included four phases	The study found a significant difference in *S. mutans* frequency in dental plaque when participants used *Magnolia* mouthwash compared to placebo or washed out Additionally, *Magnolia grandiflora* 0.3% mouthwash significantly decreased saliva bacterial colony counts after oral administration This suggests that *Magnolia grandiflora* mouthwash may be effective in reducing *S. mutans*	(Ghorbani et al. 2021)
Mangostanin (xanthone from *Garcinia mangostana* L.)	-	Antibacterial effect of mangostanin in solution containing different strains In vitro study of bacterial plaque formation Biocompatibility and antimicrobial test for periodontal gel	Mangostanin at concentrations ranged from (0.0002%:0.001) inhibited growth of *Porphyromonas gingivalis*, *Streptococcus mutans*, *Staphylococcus aureus*, and *Streptococcus pyogenes* In comparison with CHX 0.2%, MGTN 0.05% equally reduced plaque biofilm bacteria development Peridontal gel containing mangostanin 0.05% showed higher biocompatibility and comparable antimicrobial activity against *P. gingivalis* in comparison with CHX 0.2% or 0.2% enoxolone gels	(Munar-Bestard et al. 2024)
*Melaleuca alternifolia* (Maiden & Betche) Cheel.	Mouth rinse	Clinical, randomized, double-blind, parallel study	After 1 week, *Melaleuca alternifolia* decreased *Solobacterium moorei* in salivary samples (5.67 log10 copies/mL) which was higher than chlorhexidine group (5.1log10 copies/mL)	(Prabhu et al. 2022)
*Mentha spicata globulus* L. *Eucalyptus globulus* L.	Essential oils	Agar well diffusion and colorimetric microdilution methods	The essential oils of mentha and eucalyptus showed inhibition of 18.3 ± 0.47 mm and 27.0 ± 0.82 mm, and MICs of 1.8484 mg/mL and 1.9168 mg/mL, respectively, against *Streptococcus mutans*	(Landeo-Villanueva et al. 2023)
Miswak	Chewing miswak und fasting and non-fasting conditions	Crossover randomized clinical trial	Non-fasting group showed higher *Helicobacter pylori* quantities Chewing miswak sticks with toothbrushing successfully reduced the *H. pylori* counts in dental plaque of non-fasting group	(Baskaradoss et al. 2023)
Miswak (*Salvadora persica* L.)	Aqueous miswak was extracted from chewing sticks by deionized water	The study involved a cohort of patients receiving dental restorations	The addition of CHX and miswak to GIC resulted in better antibacterial qualities than traditional glass ionomer cement (GIC), but failure increased dramatically in terms of marginal defects at 9 months with CHX. This change did not significantly alter the restoration's clinical performance until the 6-month follow-up The underlying dentine of all groups exhibited a decrease in *Streptococcus mutans* counts; however, the in the group 1% drop was considerably greater (p < 0.001)	(Kabil et al. 2017)
*Murraya koenigii* (L.) Spreng	*O*-Methyl murrayamine, koenigine, koenigicine, and murrayone compounds	Molecular docking study against glycosyltransferase protein of *Streptococcus mutans*	Koenigicine, a carbazole metabolite, showed the lowest E-score, indicating a strong binding with the receptor It interacted with asparagine at position 277 in the protein	(Maheswari and Sankar 2024)
*Myrtus communis* *M. vulgar*	Aqueous and methanolic extracts	Agar diffusion and broth microdilution assays Clinical strains of *A. actinomycetemcomitans* and *E. corrodens*, along with reference strains	This method assessed the zones of inhibition produced by different concentrations of the extracts (ranging from 0.32 to 2.5 mg/disc for *M. communis* and 0.63 to 5 mg/disc for *M. vulgare*)	(Dib et al. 2021)
*Myrtus communis* L.	Essential oil	The study involved 47.6 ± 2.0 years old patients with advanced chronic periodontitis, using Gram staining, indole test, and fluorescent test to isolate *P. gingivalis* and determine essential oil's MIC	In this investigation, 30 *P. gingivalis* isolates were treated with 0.12–64 μL/mL *Myrtus communis* essence; the isolates' MIC50 and MIC90 concentrations against the essence were 1 and 8 μL/mL, respectively	(Hedayati et al. 2013)
*Nigella sativa* L.	Essential oil of the seeds	Agar well diffusion and resazurin assay methods (chlorhexidine 0.2% was used as standard)	The oil (100 µL/mL) showed comparable antibacterial activity with CHX 0.2% against *Escherichia coli*, *Staphylococcus aureus*, and *Pseudomonas aeruginosa* For the oil, *S. aureus* was the highest vulnerable bacterial strain, whereas the *E. coli* was the highest resistant one The oil effect against *C. albicans* was higher than CHX 0.2% The oil also inhibited *Lactobacillus acidophilus* at 100 µL/mL, *Aggregatibacter actinomycetemcomitans* (MIC = < 31.2 µg/ mL), *Porphyromonas gingivalis* (31.2 µg/mL), *Tannerella forsythia* (< 31.2 µg/ mL), and *Prevotella intermedia* (31.2 µg/mL)	(Bhavikatti et al. 2024
*Ocimum americanum* L.	Essential oil	Agar disk diffusion Biofilm model assay	The essential oil exhibited significant antimicrobial properties against various pathogens, including *Streptococcus mutans*, *L. casei*, and *Candida albicans*, with an MIC of 0.04% *v/v* for all tested organisms The MCC values were determined to be 0.08% v/v for *S. mutans* and *C. albicans*, and 0.3% *v/v* for *L. casei* In biofilm assays, a concentration of 3% *v/v* effectively eliminated 3 log_10_ microorganisms, while a 0.3% *v/v* concentration led to a reduction of *S. mutans* and *C. albicans* by 2 log_10_	(Thaweboon and Thaweboon 2009)
Oxyresveratrol	-	Anti-biofilm activity	Oxyresveratrol suppressed *Streptococcus mutans* growth while also reducing biofilm formation, acid generation, and water-insoluble glucan synthesis by this organism	(Wu et al. 2020)
*Phyllanthus emblica* L.	Fruit water extract that eluted from a column with glycerin	Microdilution assay and scanning electron microscopy Randomized short-term and double-blind randomized long-term trials to assess volatile sulfur compounds	*Phyllanthus emblica* concentration-dependently inhibited the growth of *Fusobacterium nucleatum* (IC_50_ = 0.079%, MIC = 0.3%), *Solobacterium moorei* (IC_50_ = 0.07%, MIC = 0.3%), and *Porphyromonas gingivalis* (IC_50_ = 0.65%, MIC = 3%) 5% extract reduced volatile sulfur compounds in both trials	(Lu et al. 2024)
*Phyllanthus emblica* L. (PE)	PE fruit extract from PE gum	Examiner-blinded, randomized, and gum-base-controlled crossover study	Chewing gum with PE fruit extract increased salivary flow and, in the short term, considerably lowered clinical test indices One safe way to enhance dental hygiene may be to chew PE gum	(Gao et al. 2018)
Pineapple hump	Ethanolic extract	Agar well diffusion method Biofilm assay method for biofilm density measurements	At concentration of 100%, pineapple hump extract can inhibit *Porphyromonas gingivalis* growth optimally, with an average zone of inhibition = 7.3 mm At concentration 50%, it can eradicate the biofilms in a 6 h incubation time	(Soulissa et al. 2021)
*Pinus pinaster* Aiton.	Standardized extract (Pycnogenol®)	Microdilution assay	The growth of all examined microorganisms, including Gram-positive and Gram-negative bacteria, as well as yeast and fungi, was effectively inhibited at minimum concentrations ranging from 20 to 250 µg/mL, corresponding to an effective concentration of 0.025% Additionally, clinical studies indicate its efficacy in preventing plaque formation and eradicating candidiasis	(Torras et al. 2005)
*Piper arboretum* Aubl.	Leaves, essential oil (EOPa)	Microdilution method	As the detected antibacterial activity of the EOPa did not reveal clinically relevant results, however when EOPa was combined with mouthwash, antibiotics (ampicillin, gentamicin, and penicillin G) and chlorhexidine to measure their predictable influence on bacterial resistance, the oil resulted in synergistic activity, decreasing the MIC of the products evaluated from 37% to 87.5%	(Matias et al. 2022)
*Piper betle* L.	Water 50% ethanol 95% ethanol	Agar disk diffusion assay	*P. betle* aqueous extract had the highest extraction yield and the 95% ethanol extract had the highest phenolic content This extract also showed the lowest minimum inhibitory concentration of all the solvent extracts against *Escherichia coli* Incorporating the extract into conventional toothpastes not only preserves its antibacterial activity, but also amplifies their total antimicrobial potential	(Ali et al. 2018)
*Piper betle* L. *Psidium guajava* L.	Aqueous extract	Serial dilution assay	The extract demonstrated antimicrobial properties, with MIC values ranging from 2.61 to 4.69 mg/mL. It decreased the hydrophobicity of cell surfaces and exhibited antiadherence properties Additionally, it displayed bacteriostatic effects	(Fathilah 2011)
*Piper betle* L.	Inflorescence extract containing safrole	Viability test using Trypan blue dye Bactericidal activity against oral pathogens Measurement of superoxide anion production using cytochrome c reduction	Safrole demonstrated no significant impact on the viability of peripheral blood neutrophils However, it effectively reduced the bactericidal activity of these immune cells against *Actinobacillus actinomycetemcomitans* and *Streptococcus mutans* in a dose-dependent manner Additionally, safrole was found to inhibit the generation of superoxide anions, which are crucial for the neutrophils' bactericidal function	(Hung et al. 2003)
*Piper mikanianum* (Kunth)	Essential oil	Microdilution method	All bacterial strains utilized in the assays had an MIC of less than 1024 μg/mL, caused by the essential oil of *Piper mikanianum* When the essential oil of *Piper mikanianum* modulating activity in combination with penicillin G, mouthwash, gentamicin, chlorhexidine, ampicillin, and ampicillin was assessed against bacterial resistance, the oil demonstrated a strong synergistic effect, lowering the MIC of the products tested collectively in a percentage ranging from 20.6% to 98.4% This study suggests expanding the range of the essential oil of *Piper mikanianum* concentration combinations and products tested, as well as toxicity assessment and in vivo testing, in an effort to find a potential low-cost mouthwash formulation that would be available to the most disadvantaged population	(Alencar Araujo Maia et al. 2024)
Piperine and cinnamaldehyde	**-**	Microbroth dilution assay	Piperine (alkaloid) and cinnamaldehyde inhibited *Candida albicans* at MICs = 32 μg/mL and 64 μg/mL, respectively The two compounds showed synergistic antimicrobial activity and six different synergistic anti-biofilm combinations were identified	(Priya and Pandian 2022)
*Pistacia lentiscus* L.	*E*ssential oil (PLL-EO)	Microdilution assay Anti-inflammatory activity through COX-1/2 and LOX inhibition Antioxidant capacity using electrochemical and MTT assays	PLL-EO inhibited oxidation by COX-1/2 and LOX enzymes, with MICs ranging from 3.13 to 12.5 µg/mL against periodontal bacteria and 6.25 to 12.5 µg/mL against *Candida* sp. The oil had negligible antioxidant activity and no cytotoxicity It exhibited broad-spectrum activity against periodontal bacteria and *Candida*, with dual inhibitory capacity against COX-2 and LOX inflammatory enzymes	(Milia et al. 2020)
*Platycarya strobilacea* Siebold & Zucc.	Leaves/ethanolic extract (PLE)	In vivo animal model; enzyme-linked immunosorbent assay kit, and quantitative real-time PCR	PLE promotes bone growth and suppresses TNF-α production and bone resorption caused by *Porphyromonas gingivalis* LPS PLE may help to maintain dental health by preventing the loss of alveolar bone caused by periodontitis	(Lee et al. 2018)
Polished rice (*Oryza sativa* L.)	Protein extracts	Inhibitory effects on gingipains, biofilm formation, bacterial growth, cell toxicity, and adhesion inhibition of epithelial cells	The extract prevented the breakdown of human proteins by *Porphyromonas gingivalis* proteinases, decreased bacterial proliferation, and inhibited biofilm development. It also minimized the attachment of *P. gingivalis* to epithelial cells	(Taiyoji et al. 2013)
Polyphenolics; eckol, dieckol, catechol, catechin, phloroglucinol, chlorogenic acid, epigallocatechin gallate (EGCG), epicatechin gallate (ECG), epigallocatechin (EGC), caffeic acid, and tannic acid	-	The deodorizing, antimicrobial, and enzyme activity assays	In comparison to other polyphenolics, catechol, dieckol, and eckol demonstrated strong antimicrobial activity against *Streptococcus mutans*, while catechol, caffeic acid, and cinnamic acid demonstrated strong antifungal activity against *C. albicans*. Dieckol, tannic acid, and ECG all significantly reduced the activity of *Streptococcus mutans*' glucosyltransferase	(Kim et al. 2017)
Pomegranate *Aloe vera* L.	Hydroalcoholic extracts of pulp from both of them	*Streptococcus mutans* was isolated from saliva and inoculated onto MSB agar	Pomegranate extract had a significantly higher inhibitory effect on *S. mutans* at all concentrations, with a significant difference observed at 50 and 100% concentrations This effect was significantly different from aloe vera and sorbitol extracts, indicating its significant antibacterial effect	(Nair et al. 2021)
Pomegranate mouthrinse	Pomegranate peel extract (PPE)	Two groups of 25 subjects each were randomly selected from among 50 healthy patients. Saliva samples were taken at three different intervals: before, after 10 min, and after 60 min. Group A received treatment with 0.2% chlorhexidine mouthrinse, while Group B received treatment with PPE mouthrinse A digital pH meter was used to measure the salivary pH, and the commercial Dentocult SM system was used to calculate the *Streptococcus mutans* count	PPE mouthrinse possesses remarkable antimicrobial activity against *S. mutans* present in the oral cavity as tested in vivo, and may be used as an adjunct to prevent dental caries and maintain good oral hygiene	(Umar et al. 2016)
Pomegranate peel methanolic extract and clove oil	Herbal toothpastes using different concentrations of the two constituents	Agar disc diffusion method using market products as standard controls	Toothpaste with 1.4% w/w pomegranate peel extract and 1% w/w clove oil showed the optimum results against *Streptococcus mutans*, *Staphylococcus aureus,* and *Candida albicans*	(Chandakavathe et al. 2023)
*Pongamia pinnata* L. *Psidium guajava* L. *Cymbopogon citratus* L. *Azadirachta indica* A.Juss.	Leaf/essential oils	Well diffusion and broth microdilution methods	*Cymbopogon citratus* essential oil showed the highest antimicrobial activity against *Streptococcus mutans* with MIC = 12 μg/mL *C. citratus* essential oil also showed almost the top results in biofilm inhibition assay, extracellular polymeric substance and acid production inhibition, *Streptococcus mutans* adherence, and time kill assay	(Pallavi et al. 2024)
Propolis	Ethanolic extract of Brazilian and one of European propolis (EEP)	Microdilution assay Scanning and transmission electron microscopy In vitro anti-biofilm	The Brazilian EEPs demonstrated low MICs against tested oral species (≤ 0.1 mg/mL to 3.13 mg/mL for *Candida albicans*), while the European EEP had slightly higher MICs The European EEP was most effective in inhibiting biofilm formation, whereas Brazilian EEPs were highly effective against preformed biofilms, reducing CFU counts by over 6 log_10_ at 100 mg/mL	(Stähli et al. 2021)
*Punica granatum* L. *Commiphora molmol* Engl. *Azadirachta indica* A.Juss.	Pericarp Nees Bark Ethanolic extracts	Disc diffusion method	Plant extracts inhibited periodontopathic bacteria growth, with *C. molmol* showing the strongest antibacterial effect against *Porphyromonas gingivalis* *P. granatum* showed no antibacterial activity against *Tannerella forsythia* When combined with antibiotics, *P. granatum* and amoxicillin showed the best synergy against *Aggregatibacter actinomycetemcomitans*	(Saquib et al. 2021)
Raspberry (*Rubus idaeus* L. var. tulameen), Red chicory (*Cichorium intybus var. silvestre*), and shiitake mushrooms (*Lentinus edodes* (Berk.) Pegler)	Low molecular mass extract	MTT assay to evaluate human gingival KB cell viability after extract treatment RT-PCR to assess the expression of genes related to cell proliferation and antimicrobial response	The LMM fractions influenced the expression of genes associated with cell proliferation, such as CK18 and β4 integrin, as well as those linked to antimicrobial responses, including HβD2 Notably, both the LMM fractions derived from chicory and mushrooms demonstrated effectiveness at low concentrations while preserving cell viability	(Canesi et al. 2011)
Red ginger (*Zingiber officinale var. rubrum*) Roscoe	Ethanol fraction of the ginger after essential oil extraction	Test the pH level on *Streptococcus gordonii* metabolism	The red ginger extracts (0.78% and 1.56%) inhibited pH decrease as it showed higher levels of pH (6.42 ± 0.078 and (6.28 ± 0.181, respectively) control group which contains the medium and 5% sucrose without the extract (4.81 ± 0.059)	(Sinaredi et al. 2024)
*Rhus coriaria* L.	Fruit/methanol extracts	*Streptococcus mutans* adherence assay using a colorimetric assay	Methyl gallate (MG) is the most bioactive component against *S. mutans* bacteria MG reduced *S. mutans* biofilm biomass on the polystyrene surface MG prevented a decrease in pH level by 97% These bioactivities of MG occurred in a dose-dependent manner and were significant vs. untreated bacteria	(Kacergius et al. 2017)
*Rhus verniciflua/ Toxicodendron vernicifluum* (Stokes) F. A. Barkley	Stokes 70% ethanolic extract	Disc diffusion method	As the concentration increases (from 1.25 mg/mL to 20 mg) the inhibition zones of *Candida albicans* increases (from 12 to 20 mm The extract at 1.25 mg/mL had a more than 99% antifungal effect against *C. albicans*, and the extract at 20 mg/mL had a 100% antifungal activity	(Kim et al. 2023)
Rice (*Oryza sativa* L.)	Rice protein fraction	A new domain model of the structure for gingipains and the hemagglutinin (HagA) proteins of *Porphyromonas gingivalis*	Seventeen proteins that interact with Arg-gingipain (Rgp) have been identified, with 4 of these proteins contributing to 90% of the observed inhibitory activity against Rgp Additionally, these proteins demonstrated an ability to impede the growth of *Porphyromonas gingivalis*	(Taiyoji et al. 2009)
*Rosa centifolia* L. *Curcuma longa* L. *Rosmarinus officinalis* L. *Punica granatum* L.	Glycolic extracts	Microdilution method	*R. officinalis* and *P. granatum* combination as well as *R. centifolia* and *C. longa* showed additive effects than each extract alone against *Candida albicans, Candida dubliniensis, Candida tropicalis, and Candida krusei*	(Meccatti et al. 2023)
*Rosa damascene* Mill. *Hypericum perforatum* L.	Aqueous, 40% ethanolic, 60% ethanolic, and enzymatic extracts	Disk diffusion and microdilution broth methods	All extracts showed antibacterial activities against different strains including *Streptococcus mutans, Streptococcus salivarius, and Porphyromonas gingivalis* In terms of MIC, the enzymatic extract of *H. perforatum* was the most potent, while the 60% ethanolic and enzymatic extracts of *Rosa damascene* showed profound results	(Antoniadou et al. 2024)
*Rosmarinus officinalis* L.	Ethanol extract	Agar diffusion method	All mouthwash solutions showed inhibitory activity, with the 10% hexane fraction showing higher sensitivity to *Candida albicans* and the 5% ethanol extract + 0.05% sodium fluoride showing higher sensitivity to *Streptococcus mutans*. The development of a growth inhibition halo in the results supported the use and correlation of *R. officinalis* extracts	(Paula et al. 2014)
*Salvadora persica* L.	Mouthwash (PersicaTM)	Double-blind, crossover trial with 28 healthy participants (aged 18–42) Participants used either PersicaTM or placebo for 3 weeks. Measurements included plaque accumulation, gingival bleeding, and salivary concentrations of *Streptococci mutans*	Both the Persica™ and placebo groups demonstrated a significant decrease in gingival bleeding (p < 0.01) However, neither group exhibited a notable reduction in plaque scores In contrast, Persica™ showed a significant decrease in the carriage of *Streptococci mutans* when compared to pre-treatment levels (p < 0.05), whereas the placebo group did not show any significant change in this regard	(Khalessi et al. 2004)
*Salvadora persica* L. (Miswak) *Cinnamomum zeylanicum* Blume (Ceylon cinnamon)	Ethanolic extracts	Synergistic antimicrobial assays	The growth and proliferation of all four strains of periodontal pathobionts were inhibited by both plants A synergistic antibacterial effect was shown when herbal extracts and several antibiotics were combined, the best was *Salvadora persica* with metronidazole against *Aggregatibacter actinomycete*mcomitans	(Saquib et al. 2019)
*Salvadora persica* L. (miswak)	Methanolic extract	An in vivo study to test the effects on dental plaque samples over 1 week Qualitative and quantitative analysis of dental plaque samples Agar disc diffusion, and microdilution methods	The Hoggar miswak extract demonstrated stronger inhibitory effects on the growth of Gram-negative bacteria than on Gram-positive bacteria The mouthwash remained stable when stored at 4°C and 25°C but exhibited alterations at 40°C. In an in vivo trial, the miswak mouthwash significantly reduced the bacterial population in the oral cavity compared to a placebo	(Chelli-Chentouf et al. 2012)
*Salvadora persica* L*.* (miswak)	Aqueous extract	Assessment of growth inhibition of *Streptococcus mutans* using agar wells Viability assessment of probiotic strains	Raw miswak demonstrated a pronounced inhibitory effect on the growth of *Streptococcus mutans*, rated at 6 + , while the miswak extract itself exhibited a moderate level of inhibition, scoring 4 + Additionally, the strains *Lactobacillus rhamnosus* 76 and *Streptococcus salivarius* also displayed strong inhibition, each receiving a rating of 4 + When combined, miswak and *L. rhamnosus* 76 significantly suppressed the presence of *S. mutans*, resulting in no viable *streptococci* detected after 48 h	(Mehanna and Reid 2010)
*Salvia officinalis* L.	Leaves of the plant were extracted in 200.0 mg/mL propylene glycol	Serial dilution assay MTT assay for cytotoxicity. Quantification of pro-inflammatory cytokines by enzyme-linked immunosorbent assay	*S. officinalis* at 50.0 mg/mL was effective against all bacteria In terms of cytotoxicity, the groups administered 50.0-, 25.0-, and 12.5-mg/mL concentrations of the extract displayed statistically comparable cell viability to the 100% viable control group A 50.0 mg/mL concentration of the extract reduced the production of TNF-α and IL-1β - The extract exhibited antibacterial efficacy against every isolate of *Streptococcus mutans*	(de Oliveira et al. 2019)
*Sambucus williamsii* var. coreana L.	Mouthwash	Randomized, double-blind, ﻿placebo-controlled study	The mouthwash showed significant results against halitosis-causing bacteria after 1 day and 5 days of treatments in comparison with a control group received only saline gargle	(Kim and Nam 2022b)
*Schizonepeta tenuifolia* Briq. *Mentha piperascens* L. *Acanthopanax sessiliflorus* Seem *Glycyrrhiza uralensis Fisch. ex DC.*	Herb Leaf Bark Root	Measuring the colony-forming units (CFUs)	The mixed herbal extracts inhibited the growth of *Streptococcus mutans*, *Enterococcus faecalis*, *Porphyromonas gingivalis*, and *Candida albicans* The colony-forming ability was decreased from 5.90 ± 0.01 to 1.48 ± 0.03 for *S. mutans*, from 5.95 ± 0.01 to 1.49 ± 0.05 for *E. faecalis*, from 5.74 ± 0.01 to 0.95 ± 0.03 for *P. gingivalis, and from 5.85* ± *0.02 to 0.90* ± *0.03* for *C. albicans*	(Yun et al. 2023)
*Scutellaria radix* L.	DMSO solution of > 95% pure Baicalin powder	Human oral keratinocytes pre-treated with varying concentrations of baicalin then treated with *Porphyromonas gingivalis* lipopolysaccharide (LPS)	Baicalin effectively reduced the expression levels of IL-6 and IL-8 induced by *Porphyromonas gingivalis* LPS, inhibits the activation of NF-κB, p38 MAPK, and JNK pathways, and also reduces the expression of genes involved in TLR signaling in response to *P. gingivalis* LPS	(Luo et al. 2012)
Sea buckthorn (*Hippophae rhamnoides* L.)	Sea buckthorn pulp oil extracted from the sea buckthorn fruits by cold-pressed extraction method, then a mouthwash was prepared	Anti-biofilm formation assay	Sea buckthorn mouthwash inhibited biofilm formation in oral microorganisms, both individually and collectively	(Smida et al. 2019)
Shiitake mushrooms (*Lentinus edodes* (Berk.) Pegler)	Low molecular weight fraction (MLMW) and subfractions 4 (SF4) and 5 (SF5) of shiitake extract	Modified constant depth film fermentor (CDFF) Dentin mineral loss quantified (TMR) Microbial shifts determined (qPCR) Acidogenicity assessed (CIA)	SF4 demonstrated a significant capacity to inhibit the demineralization of dentin, effectively contributing to oral health Additionally, it induced shifts in microbial populations, which are linked to improved oral health outcomes. Notably, there was an observed increase in the acid-producing potential of microbiota, indicating a potential disruption in glycolytic processes	(Zaura et al. 2011)
*Solidago virgaurea* L.	Ethanolic extract	Randomized, double-blind clinical study assessing the effectiveness of fluorinated toothpaste containing *Solidago virgaurea* extract (Bucovia™, Givaudan) on oral biomass	Intervention group findings: significant decrease in total bacterial load at ΔD0D28 (p = 0.005) and ΔD14D28 (p = 0.026) Reduction in *Streptococcus mutans* at ΔD0D14 (p = 0.024) Decrease in *C. albicans* at ΔD0D28 (p = 0.022) Control Group Findings: Total bacterial load showed a trend toward decrease from baseline to day 28 (ΔD0D28 p = 0.062 and ΔD14D28 p = 0.009) Clinical indices: both groups showed improvement in plaque index and gingival index	(Prêcheur et al. 2020)
Sour cherry extract	The ripe fruit extracted with a double volume of acidified ethanol (0.1% HCl), then a chewing gum with and without sour cherry extract was prepared	Measurement of the α-amylase activity of the saliva samples Microbiology measurements	Salivary α-amylase activity (sAA) and *Streptococcus mutans* levels decreased earlier in the presence of sour cherry extract than those of control cases Chewing gum with sour cherry extracts may be useful for the prevention of dental caries	(Homoki et al. 2018)
Stevia leaf extract Eucalyptus oil Peppermint oil Anise oil Tormentil root extract Myrrh extract Ratanhia root extract Clove oil	Essential oils (REPHA-OS)	In vitro anti-biofilm activity	REPHA-OS® showed statistically significant antimicrobial effects on all stages of biofilm development: an MIC of 5% could be detected for both planktonic bacteria and for biofilm formation Interestingly, only a slightly higher concentration of 10% was necessary to completely kill all bacteria in mature biofilms also In contrast, an influence on the biofilm matrix or the species distribution could not be observed The effect could be attributed to the herbal ingredients, not to the contained ethanol	(Kommerein et al. 2021)
Sunflower	Ozonated oil	Microtiter plate was used to develop microbes’ biofilms Scanning electron microscopy was used to visualize biofilm morphology In vivo model using mice to address the effect of the oil on oral candidiasis	Ozonated oil reduced the viability of all biofilms of *Candida* species, *Streptococcus mutans*, and biofilms results from both pathogens interaction	(Higa et al. 2022)
Tart cherry (*Prunus cerasus* L.)	Phenolic extract of the juice	Growth, adherence, and protease activity of *Porphyromonas gingivalis* in vitro Barrier integrity assessment: measured transepithelial electrical resistance and analyzed tight junction proteins (zonula occludens-1 and occludin)	The tart cherry extract attenuated *P. gingivalis* growth, reduced adherence to the experimental basement membrane matrix, decreased protease activities of *P. gingivalis, and* maintained the integrity of the oral epithelial barrier, preventing a decrease in transepithelial electrical resistance and protecting tight junction proteins	(Ben Lagha et al. 2021)
*Tecoma stans* (L.) *Cassia javanica* (L.)	The flowers were extracted by *n*-hexane	Serial dilution method	*Tecoma stans* and *Cassia javanica* showed significant action against *S.mutans* bacteria, which causes tooth decay In comparison to *Tecoma stans*, the flower extract from *Cassia javanica* exhibited greater activity against all tested microbes At comparatively low concentrations, the volatile oils from *Cassia javanica* flowers demonstrated possible anti-oral pathogen efficacy These two plants have the potential to replace chlorhexidine	(Mohammed et al. 2019)
*Terminalia Chebula* Retz *Terminalia Bellirica* (Gaertn.) Roxb *Embilica Officinalis* L. Triphala	Seeds/normal saline extracts	Collected salivary samples were labeled, blinded to microbiologists Mitis Salivarius Agar (MSA) with bacitracin was prepared for evaluating the *S. mutans* colony count	The four plants have antibacterial efficacy on salivary *Streptococcus mutans* count The mean colony-forming units (CFUs) of *S. mutans* with *Triphala* when compared to other three interventions were significantly reduced at 5 min and 60 min (p = 0.001) *E. officinalis* showed least reduction of mean CFUs when compared to the other three groups	(Saxena et al. 2017)
*Terminalia laxiflora* Engl.	Ethanolic and methanolic extracts	Serial dilution Glucosyltransferase (GTF) assays	The wood of *T. laxiflora* exhibited notable anticavity properties. These findings support traditional African medicine's use of this plant for oral treatment Terchebulin and flavogallonic acid dilactone may be taken into consideration for additional pharmacological investigations, evaluating the toxicity and development of a natural anticariogenic drug for dental caries, according to the encouraging results of antibacterial and GTF inhibitory action demonstrated here	(Mohieldin et al. 2017)
Thai spice *Cymbopocon citratus* (DC.) Stapf	Essential oils	Inhibition of the growth of cariogenic microorganisms (*Streptococcus mutans, Streptococcus sanguinis, and Porphyromonas gingivalis*)	*Cymbopocon (DC.) citratus* Stapf. oil was the most effective, with MIC and MBC ranging from 0.015 to 0.062 percent *v/v*. Curiously, at a concentration of 0.062 percent v/v with 96.34% inhibition, *C. citratus* oil also demonstrated strong anti-biofilm formation properties. According to a time kill study*, C. citratus* oil killed *S. mutans* and *S. sanguinis* within 12 h at concentrations that were two and four times the MIC, but it took 4 h to completely eradicate *P. gingivalis*. Furthermore, TEM showed that *C. citratus* oil directly affected *P. gingivalis's* cell wall, as evidenced by the membrane's loss of integrity. By using GC/MS and bioautography, the active ingredient in *C. citratus* was identified	(Wongsariya et al. 2014)
Thymol	Essential oil	Growth inhibition Time-kill assay - Biofilm formation analysis (sub-inhibitory concentrations) Molecular analysis/qPCR (quantitative polymerase chain reaction) In vivo studies (*Galleria mellonella* model) Toxicity assessment	Thymol at a concentration of 300 μg/mL effectively halted the growth and proliferation of both *Candida albicans* and *Streptococcus mutans* In a time kill assay, it demonstrated rapid pathogen killing within 2 min Additionally, at sub-inhibitory concentrations, thymol reduced biofilm formation and virulence factors, including yeast-to-hyphal transition and acidogenicity, in both single and dual species states qPCR analysis supported these findings Using the *G. mellonella* model, thymol showed no significant toxicity and effectively reduced infections in both single and dual species conditions in vivo	(Priya et al. 2021b)
*Thymus vulgaris* L. *Hyptis spicigera* Lam.	Essential oils	Agar diffusion assays	Both essential oils had inhibitory effects on the cariogenic species and reduced the bacterial adherence to dental enamel. Essential oils were able to disrupt preformed microcosm biofilms against *Streptococcus mutans*, *Streptococcus gordonii*, *Streptococcus sanguinis*, *Streptococcus mitis*, *Lactobacillus acidophilus*, and *Actinomyces naeslundii*	(De Oliveira et al. 2021)
Tulsi Neem Triphala Turmeric	Formulated herbal mouthwash containing the four herbal items prepared polyethylene glycol and distilled water	Double‑blinded, random controlled research Salivary microorganisms were assessed using the dilution and spread method Automated microbial colony counter for the microbial colony count	Long-term use of the herbal mouthwash formulation proved successful, and it might be used as a substitute mouthwash to get around the drawbacks of chlorhexidine	(Vinod et al. 2018)
*Usnea barbata* (L.) F.H. Wigg	Mucoadhesive oral patches loaded with ethanolic extract	Resazurin-based 96-well plate microdilution method	The patches showed moderate inhibition against *Staphylococcus aureus*, and *Pseudomonas aeruginosa,* while showed high activity against Candida albicans, and *Candida parapsilosis* with superior activity against *C. albicans*	(Popovici, V. et al., 2022)
Vitaflavan Provinols Caffeic acid *p*-Coumaric acid	Enological phenolic extracts	Serial dilution study Cytotoxicity assay Antiadherence assays	The results showed that caffeic and *p*-coumaric acids, as well as grape seed and red wine enological extracts, have antiadhesive properties When *Streptococcus dentisan*i was added, both caffeic and *p*-coumaric acids enhanced their ability to block *Streptococcus mutans* adherence	(Esteban-Fernández et al. 2018)
*Vitis amurensis* Rupr.	Leaf and stem extracts	Serial dilution method	Three compounds demonstrated inhibitory effects on the growth of *Streptococcus mutans* and *Streptococcus sanguis* within a concentration range of 12.5–50 μg/mL Among these, trans-ε-viniferin exhibited the most potent antimicrobial activity, with an MIC of 25 μg/mL against *S. mutans* and 12.5 μg/mL against *S. sanguis*	(Yim et al. 2010)
*Wrightia tinctoria* (Roxb.) R.Br *Bauhinia variegata* (L.) Benth	Leaf ethanolic extract	Agar well diffusion methods (streptomycin was used as standard)	The herbal composite of both plants showed 16 ± 0.57 mm and 15 ± 0.75 mm inhibitory zones against *Prevotella intermedia* and *Porphyromonas gingivalis*, respectively, at MIC = 20 µg/mL It also displayed anti-biofilm and anti-lactic acid production activities The herbal mouthwash of the composite showed 17 ± 1.05 mm and 17 ± 0.75 mm inhibitory zones against the two strains at MIC = 20 µg/mL Quercetin showed promising results in docking studies against biofilm producing proteins in both strains (the minor fimbrium subunit *Mfa1* and Chaperone protein *clpB*)	(Peeran et al. 2024)
Xylitol and blackberry powder	Gums contain xylitol and freeze-dried blackberry powder	Clinical randomized-controlled crossover trial	Blackberry and xylitol gums were more effective and reduced the abundance of six of nine bacteria studied in this trial (vs. 4 bacteria were reduced in xylitol group) *Streptococcus mutans* represented < 1% of the total bacteria in both groups	(Miller et al. 2022)
Zufa	0.02% Zufa prepared mouthwash	A double-blind, randomized clinical trial The Beck oral assessment scales (BOAS)	There was a noteworthy correlation discovered between the patients' oral health in the three groups (Zufa, chlorhexidine gluconate, and normal saline group) and their BOAS score following mouthwashes (p > 0.05) Zufa mouthwashes were just as efficient as chlorhexidine gluconate for improving the oral health of intubated ICU patients	(Ebrahimian et al. 2019)
α-Mangostin and lawsone methyl ether	Herbal tooth gel containing both compounds plus fluoride	Broth microdilution method	Combining the two compounds at their half concentrations increased the antimicrobial activity against *Streptococcus mutans*, *Porphyromonas gingivalis*, and *Candida albicans* -Both compounds synergistically enhanced the tooth gel’s antibioflm formation activity against all strains by up to 30%	(Nittayananta et al. 2023)

Herbal remedies are widely recognized for their traditional applications due to their soothing properties, which have significantly contributed to advancements in dental practice. Numerous plant-based products have been identified for their substantial anti-inflammatory effects. Research in this area has varied, with some studies focusing on single-ingredient formulations, while others have explored the efficacy of more complex combinations. This diversity in approach underscores the potential of herbal remedies to enhance dental treatments and improve patient outcomes (Moghadam et al. 2020). A study evaluated the efficacy of *Nigella sativa* essential oil (NSEO) in enhancing oral health by targeting periodontal pathogens, inflammation, and oxidative stress. NSEO demonstrated significant antioxidant properties, effectively reducing DPPH radicals and exhibiting high free radical-scavenging activity. The oil also showed anti-inflammatory effects by inhibiting protein denaturation and stabilizing red blood cell membranes. Notably, NSEO displayed antimicrobial activity against key periodontal pathogens, including *Porphyromonas gingivalis* and *Aggregatibacter actinomycetemcomitans*, as well as antifungal effects against *Candida albicans*. Cytocompatibility tests confirmed its safety for oral care formulations, maintaining over 82% cell viability at concentrations up to 100 µg/mL (Bhavikatti et al. 2024). Another study which was the first scientific evaluation of *Multidentia crassa* for its potential dental health benefits, reinforcing its traditional use in developing plant-based treatments for dental pain and inflammation. The research highlights the analgesic and anti-inflammatory properties of *M. crassa* extracts, which may provide effective solutions for oral health issues, especially in low-resource settings. Using gas chromatography–mass spectrometry (GC–MS) and Fourier transform infrared (FT-IR) analysis, 58 active compounds were identified, including phenols and carboxylic acids. Molecular docking studies revealed that stigmastan-3,5-diene shows strong binding potential to oral health-related proteins, indicating its promise as a natural analgesic and anti-inflammatory agent, despite some toxicity concerns for certain compounds (Chikowe et al. 2024).

Additionally, a study was conducted to investigate the effectiveness of *Phyllanthus emblica* (PE) fruit extract in managing halitosis and reducing inflammation caused by oral bacteria. PE demonstrated dose-dependent inhibition of halitosis-related bacteria, including *Fusobacterium nucleatum* and *Porphyromonas gingivalis*, while significantly lowering VSCs in clinical trials with a 5% mouthwash. Additionally, PE exhibited anti-inflammatory effects by reducing markers like IL-6 and IL-8 in oral epithelial cells. Rich in polyphenols, PE has the potential to modulate inflammatory pathways and bacterial growth, suggesting its promise as a natural ingredient for oral health and advocating for further clinical trials (Lu et al. 2024).

This study by Pan et al. investigated the effects of green tea extracts on oral inflammation and microbiome balance in mice with acetic acid-induced oral inflammation. The researchers aimed to assess whether green tea extract could serve as a natural remedy to reduce inflammation and restore microbial diversity in the oral cavity. Results showed that green tea extract significantly lowered inflammatory markers such as IL-1β and TNF-α, indicating its anti-inflammatory potential. Additionally, it promoted balanced oral microbiota by reducing harmful bacteria and aiding mucosal tissue repair. The bioactive polyphenols in green tea, particularly catechins, contributed to these protective effects. The findings suggest that green tea extract may be a promising natural therapeutic agent for managing oral inflammation and dysbiosis, warranting further clinical exploration (Pan et al. 2023).

Moreover, the study by Zhou et al. investigated the effects of *Liubao* tea extract on oral tissue regeneration, inflammation reduction, and microbiome balance. The research highlighted *Liubao* tea's potential as a natural treatment for improving oral health, particularly in repairing tissue damage and managing inflammation from oral infections. Results showed that the tea extract significantly promoted healing, reduced inflammatory markers like IL-6 and TNF-α, and helped restore a healthy oral microbiome by decreasing harmful bacteria. These findings suggest that *Liubao* tea extract may be an effective natural agent for enhancing oral health outcomes (Zhou et al. 2023).

Another research investigated the use of traditional medicinal plants for managing oral diseases, particularly focusing on their anti-inflammatory properties. Traditional medicine, relied upon by 80% of the global population, is increasingly applied in dentistry for conditions like tooth pain and gum inflammation. The study examined four plants: *Azadirachta indica* (Neem), *Terminalia chebula* (Haritaki), *Camellia sinensis* (Green Tea), and *Piper nigrum* (Black Pepper). The extracts showed significant protease inhibition, indicating their effectiveness as anti-inflammatory agents, while molecular docking studies revealed their active compounds bind well to the trypsin enzyme, suggesting potential mechanisms for their therapeutic effects in oral health (Assiry et al. 2022).

Furthermore, research examined the potential of a natural product mixture (NPM-8) in fluoride toothpaste for its antibacterial and anti-inflammatory effects, addressing the rise in oral diseases. NPM-8, derived from eight herbal extracts, demonstrated superior antibacterial performance against pathogens like *Streptococcus mutans* and *Porphyromonas gingivalis*, outperforming conventional fluoride toothpaste and chlorhexidine. It effectively disrupted biofilms and reduced pro-inflammatory cytokines, indicating its safety and efficacy for daily use. The findings suggest that NPM-8 could enhance oral health by combining antibacterial, anti-biofilm, and anti-inflammatory properties in dental care products (Qi et al. 2022).

In addition, another study investigated the antimicrobial and anti-inflammatory properties of *Pistacia lentiscus* L. essential oil (PLL-EO), derived from wild plants in Northern Sardinia, as a potential treatment for infections and inflammation, particularly in periodontal disease. PLL-EO demonstrated significant antimicrobial efficacy against key pathogens and high biocompatibility, with minimal cytotoxicity observed in oral cell lines. The oil effectively inhibited COX-2 and LOX enzymes, suggesting its potential to reduce inflammatory responses. The study concludes that PLL-EO could be a promising natural treatment option for periodontal diseases, warranting further research on its mechanisms and interactions with biofilm development (Milia et al. 2020).

Also, a study evaluated the effects of two mouthwash tinctures on oral hygiene and inflammation in orthodontic patients with dental deformities over 10 days, comparing "Octenidol" with a tincture from Georgian legume crop extract (GLCE). Results showed significant improvements in oral hygiene and a greater reduction in gingival inflammation for the GLCE group, with a 41.3% decrease in the PMA index compared to 27.8% in the control group. Additionally, GLCE increased IL-10 levels in saliva, indicating enhanced anti-inflammatory effects. The findings suggest that GLCE may be a more effective alternative to "Octenidol" for managing oral health during orthodontic treatment (Chkhikvishvili et al. 2020).

In this context, another study investigated the effects of aqueous extract (AE) from *Larrea divaricata* on oxidative stress in the submandibular glands of female rats induced by streptozotocin (STZ). The administration of STZ resulted in significant oxidative stress, marked by increased serum glucose, nitric oxide, and malondialdehyde levels. Treatment with AE restored antioxidant balance, primarily due to its major compound, nordihydroguaiaretic acid (NDGA). The AE significantly reduced oxidative damage markers and improved gland health without cytotoxic effects, suggesting its potential therapeutic applications for managing oxidative stress-related conditions, particularly in diabetes (Peralta et al. 2019).

The inhibitory effects of *Lactobacillus salivarius* WB21 was investigated on *Streptococcus mutans* and *Porphyromonas gingivalis*. Results showed that *L. salivarius* WB21 completely inhibited the growth of two *S. mutans* strains after 40 h, with significant reductions in insoluble glucan production. Additionally, it inhibited *P. gingivalis* growth within 6 h, likely due to the acidic environment it creates. The study also explored the synergistic effects of *L. salivarius* WB21 and EGCG on *P. gingivalis*, finding that EGCG enhances the antimicrobial action of *L. salivarius*. The findings suggest that a combined product could improve oral health by reducing pathogens and inflammation, warranting further clinical trials for managing oral malodor and enhancing overall oral hygiene (Higuchi et al. 2019).

The immunomodulatory effects of a propolis-containing mouthwash (P/CHX) was studied on human monocytes. Propolis was found to be non-cytotoxic, while chlorhexidine (CHX) exhibited cytotoxicity at higher concentrations. Treatment with P/CHX increased Toll-like receptor 4 (TLR-4) expression and enhanced interleukin-10 (IL-10) production, which may inhibit co-stimulatory molecules like CD80. The combination also slightly activated the NF-κB signaling pathway and improved monocytes' bactericidal activity against *Streptococcus mutans*, potentially through reactive oxygen species generation. Additionally, P/CHX reduced TNF-α production from CHX, indicating anti-inflammatory potential. Overall, the findings suggest that P/CHX could be beneficial for managing periodontal diseases due to its enhanced microbicidal and anti-inflammatory properties (Santiago et al. 2016).

The antioxidant and cytoprotective activities of betel quid and its components were investigated, where the study highlighted their potential health benefits in the oral cavity. Using an aqueous extraction method, gambir was found to have superior antioxidant activity compared to areca nut, with effects similar to ascorbic acid. Betel quid containing calcium hydroxide showed reduced antioxidant capacity due to higher alkalinity. The total phenolic content in gambir correlated positively with antioxidant activity and cytoprotection against oxidative stress in human gingival fibroblasts. Key compounds like quinic acid and quercetin contributed to these effects, while the stability of phenolics was influenced by pH levels. The findings suggest that betel quid may improve oral health, warranting further clinical investigation into its bioactive compounds for periodontal disease prevention (Nur Sazwi et al. 2013).

The endocannabinoid system (ECS) plays a crucial role in cognitive functions, inflammation, and immune response, particularly during periodontal disease, where inflammatory mediators can elevate pro-inflammatory cytokines. Recent studies on plant cannabinoids (pCBs) revealed that while they showed limited activity at the CB1 receptor, compounds like CBG and CBVN acted as agonists at the CB2 receptor, promoting anti-inflammatory effects. pCBs enhanced the viability of human gingival fibroblasts and modulated cytokine production, suggesting their potential as therapeutic agents for managing periodontal inflammation and pain while reducing harmful bacteria (Abidi et al. 2022).

As is known, cigarette smoke contains over 5,300 chemicals, including nicotine and carcinogens, which generate free radicals that cause oxidative damage to lipids, proteins, and DNA. This oxidative stress triggers an inflammatory response through the activation of IκB kinase (IKK) and the degradation of NF-κB inhibitors, leading to increased expression of pro-inflammatory genes. Smoking significantly raises the risk of oral diseases like gingivitis and periodontitis. A study investigated candy lozenges containing extracts from *Moringa oleifera* and *Cyanthillium cinereum*, hypothesizing that their antioxidant and anti-inflammatory properties may alleviate oral inflammation in smokers (Luetragoon et al. 2021).

Another epidemiological research has linked polyphenols in black tea, particularly theaflavins, to various health benefits, including potential protective effects against oral diseases. This study evaluated the antibacterial efficacy of black tea extract and its theaflavin derivatives against key periodontopathogens like *Porphyromonas gingivalis* and *Prevotella intermedia*. The findings showed that black tea extract and theaflavins effectively inhibited these pathogens and enhanced the antibacterial effects of conventional antibiotics. Additionally, black tea extract reduced IL-8 production, potentially lowering inflammation in periodontal diseases. The study suggested that incorporating black tea bioactive compounds into oral hygiene products could be a cost-effective strategy for improving periodontal health (Lombardo Bedran et al. 2015).

A comprehensive list of several herbal remedies utilized to enhance oral health is presented in Table 2. These remedies have been recognized for their therapeutic properties and potential benefits in dental care, contributing to the prevention and management of various oral conditions.

**Table 2:** Herbal remedies used for enhancing oral health

Plant name/plant compounds	Part used/type of the extract/dosage form	Method	Results	Ref
*Aloe Vera* L	Juice	Clinical trial, to compare the antiplaque effects of mouthwash containing aloe vera versus mouthwash containing 0.2% chlorhexidine gluconate on the development of new plaque The 90 healthy participants in this randomized, single-blind, parallel, controlled clinical trial had an average age of 27.19 ± 12.08 years. Following comprehensive oral prophylaxis, participants were told to stop using mechanical plaque management. Participants were split into three groups at random; the test group received mouthwash made entirely of aloe vera; the control group received mouthwash containing 0.2% chlorhexidine gluconate; and the placebo group rinsed their mouths twice a day with flavored distilled water By contrasting pre-rinsing, the impact on 4-day de novo plaque development was evaluated. Hein–Quigley and Student's t test and analysis of variance were used to statistically examine the modified plaque scores	The test group's plaque score was similar to that of the post-rinsing control group. The test group and the control group both significantly differed from the placebo group. Aloe vera-based herbal mouthwash is a viable substitute for the gold standard 0.2% chlorhexidine gluconate, since it has less adverse effects and is just as effective at preventing plaque	(Chhina et al. 2016)
*Aloe vera* L.	-	Clinical trial Three groups were randomly selected from among 75 patients with burning mouth syndrome (BMS): Group I received tongue protection three times a day, Group II received tongue protector and 0.5 ml of AV at 70% three times a day, and Group III received tongue protector and 0.5 ml of placebo three times a day The Hospital Anxiety–Depression scale was used to analyze the psychological profiles of the patients, the Oral Health Impact Profile 49 (OHIP-49) was used to evaluate their quality of life, and the visual analog scale (VAS) was used to evaluate the symptoms. The course of treatment lasted for 3 months	All three study groups saw improvements in visual analog scale pain scores; however, there were no statistically significant group differences (p = 0.210). Except for the handicap score on the OHIP-49, there were no discernible changes in the quality of life across the groups. Group II experienced a larger overall clinical improvement, with a difference that nearly reached significance. Patients with burning mouth syndrome respond well to concurrent prescriptions of *Aloe vera* and a tongue guard	(López‐Jornet et al., 2013)
*Anacyclus pyrenthrum* L. *Punica granutum* L. *Capparis spinosa granutum* L. *Quercus infectoria* Oliv	Entire plant Pericarp Galls Root bark/aqueous extract	For 30 days, 110 schoolchildren were divided into two groups at random: the test group, which received herbal mouthwash containing extracts from *Anacyclus pyrenthrum* DC, *Quercus infectoria* Oliv (galls), *Punica granutum* (pericarp), and *Capparis spinosa* (root bark), and the control group, which received mouthwash containing 0.2% chlorhexidine. To ascertain the intervention's sustainable effect, the response was evaluated using the following metrics: DMFT, gingival index (GI), oral hygiene index-simplified (OHI-S), plaque index (PI), salivary pH, plaque index simplified (PI-S), and bleeding on probing (BOP) on baseline and 30th day. GI and PI were also evaluated on 60th, 90th, and 120th days	Following the treatment, both groups' mean GI, PI, PI-S, OHI-S, and BOP considerably decreased on day 30 from baseline (p < 0.001). On the 120th day from baseline, both groups had significantly lower PI and GI scores after the intervention was stopped (p < 0.001). According to this study, mouthwash that has been made is both safe and highly effective in preserving dental health	(Fahim 2024)
*Barleria prionitis* L.	Bark/ethanol extract	Clinical trial, to assess the oral health benefits of *Barleria prionitis* extract mouthwash in contrast to the industry standard, chlorhexidine (CHX) mouthwash Thirty subjects in all were split into two groups at random: the CHX gluconate mouthwash group and the *B. prionitis* group Three days and the baseline were when the data were gathered The Quigley and Hein plaque index, which was modified by Turesky–Gilmore–Glickman, was used to record their scores after the plaque was shown using an erythrosine revealing agent The impact of the two medication regimens was compared using statistical analysis	According to our findings, the effectiveness of *B. prionitis* and chlorhexidine against dental plaque was statistically equivalent. Even so, chlorhexidine’s action was more noticeable This study supports the use of *B. prionitis* in folklore as a preventive measure against oral microbial infections by confirming the plant's antibacterial properties	(Gupta et al. 2016)
Black currant	-	Clinical trial, to examine the short-term, time-dependent effects of smoking and eating black currants on healthy smokers' salivary flow rate (SFR) and salivary IgA secretion rate (sIgA SR) In healthy smokers (n = 8), SFR, sIgA levels in saliva, and sIgA SRs were measured eight times during three consecutive interventions: baseline, 5, 30, and 60 min after smoking, 5, 30, and 60 min after consuming 100 g of black currants and then smoking, and 5 min after consuming black currants. Black currant eating before smoking reduced the significant delayed effect of smoking on SFR evaluated 60 min after smoking (P = .03). sIgA flow rates and salivary IgA concentrations were not significantly affected by smoking	Consuming black currants before smoking caused sIgA concentrations to significantly drop 5 min after the intervention relative to the baseline (p = 046), and then show a statistically significant upward trend 60 min after the intervention (p = 025). Even though quitting smoking is the most effective way to prevent chronic illnesses, the results indicate that eating black currants may help lower the risk of smoking-related oral health issues by reducing the detrimental effects of tobacco smoke on salivary flow and salivary immunological status	(Konić-Ristić et al. 2015)
*Blighia sapida* K.D.Koenig	Fruit in Jamaica (ackee, *Blighia sapida*)	Hypothesis-driven research article The study explores the theoretical potential of saponins as a safer alternative to sodium lauryl sulfate in toothpaste. It discusses the extraction of saponins from ackee and their possible application in toothpaste	It proposed that saponins from ackee, *B. sapida* fruit could be a viable alternative to sodium lauryl sulfate in toothpaste	(Paul et al. 2020)
Brown algae, *Ascophyllum nodosum* (L.) Le Jolis	Edible treats containing brown algae	In vivo (dogs), to assess the impact of giving dogs edible treats containing the brown algae *Ascophyllum nodosum* for 90 days on the buildup of dental calculus and plaque on their teeth as well as on other indicators of canine oral health, such as the concentration of volatile sulfur compounds (VSC), plaque index (PI), calculus index (CI), oral health index (OHI), gingival bleeding index (GBI), and calculus index (CI) After having their teeth professionally cleaned, 60 client-owned dogs—including Japanese chin, Pomeranian, Chihuahua, miniature Schnauzer, and West Highland White Terrier (WHWT) breeds—were split into two groups at random and given daily edible treats that contained the brown algae *A. nodosum* or a placebo that was based on their bodyweight	In the dogs under investigation, the ingestion of edible treats containing *A. nodosum* effectively reduced the buildup of calculus and plaque. In comparison to the placebo-control group, dogs treated with *A. nodosum* also showed noticeably lower VSC concentrations and improved oral health status (e.g., OHI and GBI)	(Gawor et al. 2018)
*Cannabis sativa* L.	Oil	Pilot study, clinical trial The cannabis plant extract, made from standardized plant material (cannabis flos) at specialty pharmacies using Romano–Hazekamp extraction and diluted in oil (1 g of cannabis in 10 g of olive oil) was administered to the subjects for 4 weeks	Over time, the subjects' clinical remission of the oral symptoms improved statistically significantly. Anxiety and despair levels also showed a positive improvement, changing statistically. The *C. sativa* oil used in this pilot study was beneficial and well tolerated by patients with primary burning mouth syndrome (BMS)	(Gambino et al. 2021)
*Centella asiatica* (L.) Urban	Leaves/aqueous extract	A single group randomized controlled interventional study (pre- and post-intervention) was carried out with participants who were hyperglycemic and had poor oral health status	According to the results of the current investigation, using *C. asiatica* aqueous extract twice a day for 3 months as a mouthwash has an immunomodulatory effect	(ShanmugaPriya et al. 2023)
Chamomile *Chamaemelum nobile* L.	2% gel consistency	Clinical trial, to assess the effectiveness of topical 2% chamomile gel treatments for burning mouth syndrome (BMS) in contrast to a placebo The research was planned as a prospective, double-blind, randomized, placebo-controlled, monocentric study. Two groups of 62 individuals with idiopathic BMS were created: Group A was given 2% chamomile gel applications, while Group B (placebo) was given a placebo. Both groups got these treatments twice a day for a month. At baseline, 15, and 30 days, three factors were assessed: oral quality of life (measured with the Oral Health Impact Profile-14), xerostomia severity (measured with the Xerostomia Inventory), and pain (measured with a visual analog scale [VAS])	The trial was finished by 57 patients in total. At 15 and 30 days, both groups experienced statistically significant improvements in pain, xerostomia, and quality of life (P < 0.001). However, when comparing the two groups, there were no significant differences in oral quality of life (P = 0.076), xerostomia severity (P = 0.536), or VAS pain (P = 0.847). The product made from chamomile gel was favorably received. The effectiveness of 2% chamomile gel in treating burning mouth syndrome (BMS) seems dubious because the results of chamomile treatment and the placebo were comparable. To verify this, though, more research with bigger patient samples is required	(Valenzuela et al. 2016)
Cranberries	Juice	Clinical trial, 41 diabetic patients (aged 35–67) with periodontal disease were enrolled in this randomized clinical trial and were randomized to one of four groups: control (C; n = 12), receiving omega-3 (I1; n = 10, 1 g/ twice daily), cranberry juice (I2; n = 9, 200 ml, twice daily), or cranberry juice enriched with omega-3 (I3; n = 10, 200 ml, containing 1 g omega-3) twice daily for 8 weeks	In individuals with diabetes who also have periodontal disease, cranberry juice enhanced with omega-3 can be used as an adjuvant therapy in conjunction with nonsurgical periodontal therapy to improve periodontal state, increase HDL-C, and lower glycated hemoglobin	(Zare Javid et al. 2018)
Erythritol (sugar alcohol)	5% w/w erythritol water	Three groups of mice were identified: 6-week-old (YC) and 18-month-old (AC) control groups; and a group that was given 5% w/w erythritol water for 6 months (AE). Following raising, the gingiva was used to extract RNA, and PCR was used to determine the concentrations of chemicals linked to aging. The aging markers p21, γH2AX, NF-κB p65, p16, p21, γH2AX, IL-1β, and TNFα m were all immunostainable The gingiva of the AC group exhibited higher levels of RNA expression compared to the YC group. However, the AE gingiva showed a considerable suppression of this elevated expression	In both a mouse model and a cell culture system, erythritol prevented gingival tissue and fibroblast senescence and attenuated age-related histological alterations in gingival tissue. Erythritol modifies the glycolytic system to control cellular senescence It is anticipated that new applications of erythritol in the oral cavities will result in the creation of oral care techniques that aim to prevent the aging of oral tissue	(Yokoi et al. 2023)
Thymol Menthol Eucalyptol	Essential oils	Hypothesis-driven research article This supplement offers convincing proof that using essential oil mouthwashes can be a healthy and secure addition to regular oral hygiene practices	According to some experts, tooth erosion may result from mouthwashes with a pH lower than 5.5. Research has indicated that even though an essential oil mouthwash has a pH below 5.5, salivary pH stays above 5.5 for 15 min after rinsing	(Claffey 2003)
*Glycyrrhiza* *glabra* L.	Root powder/aqueous extract	Five groups consisting of 155 dental students were selected A placebo and a chewing gum with *Glycyrrhiza* *glabra*, honey, and vitamin E were made independently Every student completed two surveys, one before and one after the intervention Salivary bacteria count, bleeding on probing, decay missing filling teeth (DMFT) index, gingival index, and plaque index were measured prior to and during the intervention Stata 14 was the program used for all statistical studies	Following the intervention, all groups showed a decrease in bleeding on probing, the gingival index, and plaque index In the case group, gingival inflammation showed the greatest reduction. Following the intervention, salivary bacterial counts decreased in each group. The case group experienced the largest decline	(Ahmadi-Motamayel et al. 2024)
Grape seed and grapefruit	-	Clinical trial For 60 days following the dental extraction procedure, male participants in this randomized pilot study continued to take either grapefruit extract (GFE) or grape seed extract (GSE) for 2 weeks before the procedure	Overall, grape seed extract resulted in a decrease in collagen density and an increase in osteoclasts, while grapefruit extract demonstrated a downregulation of inflammation. This pilot study emphasizes the need for more research on how these supplements affect dental health and bone repair	(Souza Jr et al., 2020)
Green Brazilian propolis	Ethanol	This study is concerned with the extract possible ability to modulate inflammation when exposed to a periodontal or *Candida* biofilm that contained monocytic (MONO-MAC-6) cells	It appears that the toothpaste formulations that contain the ethanolic extract of green Brazilian propolis appear to hold promise for preventing periodontitis, gingivitis, and dental cavities	(Coluccia et al. 2022)
Green tea	Leaves	Clinical trial In the shape of green tea dip bags, fresh green tea (Lipton green tea bags, packing date < 1 month) was purchased at the neighborhood market. Two grams of green tea dip bag were dipped in 100 ml of warm water for 5 min (10 ml per participant) to create 2% green tea	According to the study, green tea and probiotic mouthwash have positive impacts on creating an alkaline environment that is good for kids' oral health	(Manikandan et al. 2020)
Green tea	Green tea polyphenol ( −)-epigallocatechin gallate (EGCG)	Clinical trial, to study the interaction between ( −)-EGCG and saliva, particularly in relation to EGCG–protein binding, because tea interacts with saliva when it enters the mouth Funakoshi (Tokyo, Japan) supplied the green tea polyphenol ( −)-EGCG (purity > 98%), which was then diluted in water to create a stock solution with a pH of 2.7 and a concentration of 5 mg ml − 1 Five participants who provided their informed consent to participate in the study had their human unstimulated saliva collected: samples A, B, C, D, and E were from a 47-year-old male, a 21-year-old woman, a 36-year-old woman, a 54-year-old woman, and a 62-year-old woman Clinical evaluations revealed that every volunteer was healthy and free of dental problems. They did not use any chemical mouthwashes for 3 h before sampling, and they had not taken any medicine for 3 months before the trial	Through non-competitive inhibition, EGCG reduced alpha-amylase activity, suggesting that EGCG is useful in preventing the production of fermentable carbohydrates that contribute to the development of cavities. Interestingly, EGCG's antibacterial action against the periodontal bacterium *Aggregatibacter actinomycetemcomitans* was diminished by alpha-amylase. As a result, we thought that interactions between EGCG and salivary proteins would affect dental health in both positive and negative ways	(Hara et al. 2012)
Green tea-added tablets	-	Clinical trial, the effectiveness of sugar-free tablets containing green tea extract on oral volatile sulfur-containing compounds (VSC) was evaluated in a controlled, clinical, double-blind, crossover research for 30 min in comparison to placebo tablets Participants had to have at least 24 teeth, no history of systemic or oral illnesses, and no detachable dentures to be eligible for the study. A 2-week period of abstinence from professional oral hygiene and medications, menstruation, brushing of the teeth and tongue, smoking, alcohol, coffee, tea, onions, garlic, and licorice was required of all eligible participants. Additionally, their VSC level during the basal measurement had to be > 75 ppb. After at least 48 h of wash-out, subjects were placed into their groups. 0.05% green tea extract (or 1 mg polyphenols for three tablets) was present in the test tablet (0.7 g); the control tablet was the same, but lacked the active ingredient	The experiment had 54 participants (31 women and 23 men). No issues related to green tea were reported. At the conclusion of tablet sucking, the control group's mean VSC level reduction from baseline was 34% (p < 0.001), while the test group's was 55% (p < 0.001). Thirty minutes later, the control group's VSC level reduction was 7% (p = NS), while the test group's was 26% (p < 0.005). Following baseline adjustment, the two groups' comparisons revealed a statistically significant difference in decreases at the conclusion of the sucking session (p < 0.01) and 30 min later (p < 0.01). Green tea extract tablets can statistically lower oral VSC levels both right away and half an hour later. Additionally, compared to the control tablets, the test tablets considerably decreased oral VSC	(Porciani and Grandini 2016)
Himalaya Complete Care contains Neem Pomegranate (*Punica granatum* L.) *Triphala*	Herbal toothpastes	Clinical trial, the study included individuals with elevated blood sugar levels to assess the efficacy of the herbal formulations in managing diabetes	There is a tendency to assume that herbal toothpastes have strong salivary glucose inhibitory action and prefer a brief elevation of salivary pH	(Khairnar et al. 2017)
*Houttuynia cordata* Thunb	Aqueous extract	For 18 h, primary gingival epithelial cells (GECs) were exposed to different extract concentrations Using qRT-PCR, the gene expression of hBD2, SLPI, cytokines, and chemokines was assessed ELISA or the Luminex assay was used to identify the secreted proteins in the culture supernatants The CellTiter-Blue Assay was used to evaluate the extract's cytotoxicity	Without causing cytotoxicity, *H. cordata* dramatically increased the expression of hBD2, SLPI, IL-8, and CCL20 in a dose-dependent manner. The extract dramatically increased the levels of IL-2, IL-6, IL-8, and IFN-γ while also modulating the secreted hBD2 and SLPI proteins. According to the study, *H. cordata* has the ability to alter oral innate immune mediators The development of novel topical medicines from *H. cordata* for the prevention and treatment of immune-mediated oral disorders may result from these findings	(Satthakarn et al. 2015)
Indian propolis	30% ethanol	In vitro, to assess the impact of a 30% ethanolic extract of Indian propolis on dentinal tubule occlusion in comparison to GC tooth mousse, a desensitizing product that contains Recaldent™ Thirty recently extracted, healthy human third molars were used to create the specimens, which were then kept at room temperature in 10% formalin (pH 7.0) A sectioned sample measuring 5 mm in length, 5 mm in width, and 3.5 mm in depth was taken from each specimen, encompassing the cervical region. Using diamond pastes and aluminum oxide abrasive paper with grits of 1000 and 1200, samples were smoothed and wet-polished to enhance the clinical aspect of hypersensitive dentin cervical surfaces. Three groups of ten specimens each were randomly assigned based on the dentin surface treatments. Test Group: 30% ethanolic extract of Indian propolis (n = 10); Positive Group: GC Tooth Mousse (n = 10); Negative control: Untreated specimens (n = 4) and prepared with 6% citric acid (n = 6). Every specimen was ready for SEM examination	By forming crystal-like deposits in the tubule lumen, GC tooth mousse facilitated tubule blockage. On the surface of the dentin, propolis formed a thin, smooth coating	(Hongal et al. 2014)
Lemongrass tea tree	Essential oils	In vivo study, saliva pH levels before and after using lemongrass oil and tea tree oil mouthwash will be compared in this study to assess its relationship to dental health Ninety volunteers between the ages of 26 and 38 made up the study's purposive sample. A comparative analysis was conducted utilizing the GC PH strips to measure the PH before and after using mouthwashes containing chlorhexidine, lemongrass oil, and tea tree oil	Using the Wilcoxon signed-ranks test for comparison, it was discovered that the tea tree oil increases salivary pH more than other methods, with a statistically significant p = 0.001** (p < 0.05). The results of the study demonstrate the advantages of tea tree oil and lemongrass oil mouthwash	(Manikandan et al. 2021)
Linseed	Linseed extract Salinum®	Clinical trial, to investigate how mouthrinses containing salivary replacement agents (linseed extract) affect dental health in individuals with primary Sjögren's syndrome Salinum®, a linseed extract, either by itself (Sal) or in combination with chlorhexidine (Sal/Chx), was used for mouth rinsing between 3-week intervals between rinses and a 3-week "wash-out" phase. Microbiological analyses, mirror friction tests, and the percentage of areas with tooth plaque and bleeding on probing. Survey on the symptoms of decreased salivation in the mouth	Following linseed extract and after linseed extract/chlorhexidine, there was less dental plaque and bleeding on probing. Following both treatments, there was less friction. The total number of anaerobically cultivated microorganisms and mutans streptococci decreased after linseed extract/chlorhexidine (p < 0.05 and p < 0.001); however, there were no discernible changes in the counts of the microbial groups under study after linseed extract. After taking linseed extract and linseed extract/chlorhexidine, the symptoms of dry mouth improved (p < 0.05 and p < 0.001, respectively). Sal helped with speaking issues and burning mouth symptoms (p < 0.05)	(Johansson et al. 2001)
Magnolia bark	-	Clinical trial, the purpose of this study is to compare the effects of chewing gum containing sorbitol, both with and without Magnolia bark extract, on the hydrophobicity of the tooth surface, the composition of the salivary film, and the self-perceived mouthfeel In a crossover research trial, participants chewed gum containing sorbitol three times a day for 4 weeks, either with or without the addition of magnolia bark extract. As a mastication control, some volunteers also chewed parafilm Questionnaires were used to quantify intra-oral water-contact angles prior to, immediately following, and 60 min following eating, as well as oral moistness and tooth smoothness. The coating of 1.5 g pellet-shaped chewing gums including gum base, sorbitol, flavoring agents, sweeteners, and coolants was supplemented with 3 mg of magnolia tree extract (Honsea Sunshine Biotech, Guangzhou, China)	The hydrophobicity of the tooth surface and the makeup of the salivary film were unaffected by chewing parafilm. Therefore, better self-perceived mouthfeel is caused by sorbitol adsorption rather than the presence of magnolia bark extract or enhanced salivation	(Wessel et al. 2017)
*Nigella sativa* L.	*Nigella sativa* oil (NSO)	Local dental formula of *Nigella sativa* oil (NSO) was investigated for periodontal diseases treatment	The produced *Nigella sativa* oil nanoemulgel revealed potential for the healing of periodontal diseases and to create nanoemulgel formulations for preclinical and clinical testing, *Nigella sativa* oil can be mixed with other natural or synthetic antimicrobial agents	(Sultan et al. 2022)
*Ocimum gratissimum* L.	Stem barks/essential oil	In vivo, this study's objective was to assess *Ocimum gratissimum's* (Og) antiplaque effect Initially, 1 mL of essential oil was diluted in 9 mL of distilled water (1:9), preparing a 10% mixture (*v/v*) (Og solution) A mouth rinse containing only distilled water (DWsolution) and other containing 0.12% chlorhexidine digluconate (CLX solution) were formulated too In all groups, a very small amount of menthol (flavoring), color and conserving agent were added. A crossover, double-blind clinical trial utilizing a 3-day partial-mouth plaque buildup paradigm involved 15 healthy participants. The participants were randomly randomized to use just the following mouthrinses at first: 10% Og (Og solution), 0.12% chlorhexidine digluconate (CLX solution), or distilled water (DW solution). They also stopped using any mechanical oral care techniques. At the conclusion of the trial, the plaque index (PLI) was measured in each mandibular tooth, and the difference between the groups was estimated using the Kruskal–Wallis (α = 0.05) and Mann–Whitney (α = 0.05) tests	The chlorhexidine digluconate and *Ocimum gratissimum* solutions were preferred in clinical outcomes, which revealed a statistically significant difference between the groups (p < 0.05). However, the first solution was more successful (p < 0.05). Plaque regrowth was inhibited by mouthrinses containing 0.12% chlorhexidine digluconate and 10% Og; however, *Ocimum gratissimum*'s effects were less pronounced than chlorhexidine digluconate's	(Pimenta et al. 2016)
Peppermint	Essential oil	Clinical trial, to assess how often halitosis is and how peppermint mouthwash affects it There were two stages to this investigation. In a cross-sectional study, 504 adolescents between the ages of 14 and 18 years were screened to identify those who have halitosis. The 84 students who were chosen for the study were then randomly assigned to one of two groups. In total, 41 students in one group received a placebo, whereas 43 students in group 1 received a peppermint mouthwash. Over the course of a week, the students in two groups rinsed their mouths three times with 15–20 ml of the provided solutions (after breakfast, lunch, or after they got home, before bed), and they abstained from eating for 30 min afterward. The students were re-examined after a week	In total, 24.4% of people had halitosis. After a week, 11 students in the placebo group had halitosis, while 23 students in the mouthwash group did not. This difference was found to be significant using a Chi-square test. According to the study's findings, halitosis can be lessened by using peppermint mouthwash	(Haghgoo and Abbasi 2013)
Pomegranate Propolis	Pomegranate and propolis extracts	In vivo, the purpose of this study was to create baked cookies with propolis and pomegranate extracts and assess how they affected dogs' oral health in comparison to sodium hexametaphosphate Creation of pomegranate and propolis-infused baked cookies for dogs' dental health	Pomegranate extract biscuits had a similar impact to those containing sodium hexametaphosphate in lowering the area covered by dental calculus in dogs	(Santos et al. 2021)
*Psidium guajava* L.	Leaves/hydroethanol	Five groups of 170 Sprague–Dawley rats were randomly assigned. As a mouthwash, guajava leaf extract and phenytoin were utilized On days 7 and 10, 12 rats from each of the five groups were put to death, and on day 14, ten rats from each group were slaughtered Serum levels of interleukin-6 and total antioxidant capacity were measured Assessments of pathology and stereology were conducted on the tissues To ascertain the antioxidant efficacy of *Psidium guajava* L. hydroalcoholic extract, phytochemical studies were carried out	The total phenolic content and the DPPH study showed that *P. guajava* L. has a strong potential for antioxidant capacity. IL-6 was shown to decrease, whereas TAC increased in the phenytoin and guajava hydroalcoholic extract groups. *P. guajava* L leaf hydroalcoholic extract may have therapeutic benefits for oral mucositis	(Ghaderi et al. 2022)
Sunflower	0.1% vitamin E acetate and 0.5% sunflower oil (with vitamin F)	Clinical trial, to evaluate a new oral health toothpaste's antiplaque/antigingivitis efficacy to that of a clinically proven control toothpaste by measuring the delivery of its active components, which include zinc citrate trihydrate/triclosan, α-tocopherol acetate (vitamin E), and sunflower oil (vitamin F) 0.1% vitamin E acetate (α-tocopherol acetate) and 0.5% sunflower oil, which is a source of vitamin F (linoleic acid), were present in the new toothpaste Additionally, it contained a proven gum-health active system (0.3% triclosan and 0.75% zinc citrate trihydrate) and an anticaries agent (0.32% sodium fluoride). Three investigations were conducted. Twelve hours after using the test toothpaste, the bioavailability of triclosan and zinc in plaque was assessed in study 1 (n = 45). Study 2 (n = 93) used a randomized, parallel, double-blind, controlled design to examine the test toothpaste's impact on gingival and plaque health over the course of 3 weeks. Fluoride toothpaste with 0.3% triclosan was used as the control. After being recruited, healthy adult volunteers with GI > 1.0 received a full mouth scale and polish. At baseline and 3 weeks later, gingival health (measured by the gingival index) and plaque levels (measured by the Modified Quigley and Hein Index) were evaluated. The duration of Study 3 (n = 93) was 9 weeks. The study was the same as study 2, with the exception that subjects did not receive a full mouth scale and polish at baseline	The mean concentration of triclosan in baseline plaque samples in study 1 was 5.78μg/g (std = 4.74), while the mean concentration of zinc increased from 15.2μg/g zinc ion in baseline plaque samples to 84.3μg/g zinc ion (p > 0.0001) in samples taken 12 h after brushing. After 3 weeks, there was no discernible difference between the test and control groups in study 2, although mean plaque and gingival indices were much lower in both groups than at baseline. There was no significant difference between the test and control groups after three and 9 weeks, but mean plaque and gingival indices were significantly lower in both groups after 3 and 9 weeks compared to baseline in study 3. The addition of α-tocopherol acetate (vitamin E) and sunflower oil (vitamin F) had no effect on the delivery of zinc and triclosan from a new oral health toothpaste that contained zinc citrate trihydrate and triclosan. The new toothpaste was just as successful as a clinically validated positive control at lowering plaque levels and enhancing gingival health. Thus, using this toothpaste for regular dental hygiene helps to keep gums strong and healthy	(Schäfer et al. 2007)
*Syzygium aromaticum* (L.) Merr. & L.M.Perry *Juglans regia* L. *Ammi visnaga* L.	Floral bud Bark Leaf flower Stem	A standardized questionnaire (survey) was used to perform a cross-sectional survey Other treatments including alum, salt, and vinegar have also been studied in addition to medicinal plants The two groups underwent statistical calculations based on use value, relative frequency of citation, family use value, informant consensus factor, and fidelity level	Several types of medicinal plants have been found to be beneficial for dental health, including *Ammi visnaga*, *Juglans regia*, and *Syzygium aromaticum* The majority of participants stated that they used these plants to cure and prevent toothaches, gingivitis, and dental cavities It was discovered that people who practice herbal medicine know more about plants and their applications than the average person. Nonetheless, a sizable fraction of participants including herbalists—selected contemporary medicine or dental care and extraction as their preferred forms of treatment, whether in conjunction with or apart from conventional therapies Fortunately, combining these plants with contemporary dentistry can maximize preventative measures for oral health	(Benabderrahmane et al. 2024)
Tea Tree Propolis	Essential oil Ethanolic extract	Clinical trial, this study sought to determine how toothpaste containing ethanolic extract of propolis (EEP) and natural tea tree essential oil (TTO) affected oral health indicators and microbiota in patients wearing removable acrylic partial dentures Two groups of 50 patients with different levels of hygiene were created. The toothpaste containing tea tree essential oil and ethanolic extract of propolis was given to the study group, while the toothpaste containing neither was given to the control group	The quantitative decrease of oral microbiota was greatly impacted by the use of toothpaste containing used natural antimicrobial compounds, such as tea tree essential oil, and ethanolic extract of propolis. This finding supports the antimicrobial and antifungal properties of the used natural antimicrobial substances	(Wiatrak et al. 2021)
Tulsi Sesame seeds Fennel seeds Coconut	Natural chewable products	Clinical trial, to assess how Recaldent chewing gum and natural chewables (tulsi, sesame seeds, fennel seeds, and coconut) affect the pH, calcium, and phosphate concentration of plaque	With the exception of the coconut group at 30 min and the fennel group at 5 min, the mean pH in each study group rose after 5 and 30 min relative to the baseline The fennel group had the largest rise in plaque calcium concentration, followed by the Recaldent and sesame groups, in that order On the other hand, the Recaldent group had the largest rise in plaque phosphate, followed by the sesame and fennel groups, respectively. Plant-based products can be accessible, affordable, and efficient ways to keep your teeth healthy. It is advised to conduct more research to verify long-term impacts	(Sultan et al. 2016)
Olive	Lycopene-enriched virgin olive oil	Clinical trial, to compare the therapeutic efficacy of lycopene-enriched virgin olive oil with a placebo to assess its clinical performance on burning mouth syndrome (BMS) 60 BMS patients were split into two groups at random: Group I (n = 30) received lycopene-enriched virgin olive oil (300 ppm) (1·5 mL three times a day), and Group II (n = 30) received a placebo (1·5 mL three times a day). Assessments were conducted both before and after the product or placebo was used for 12 weeks. VAS was used to evaluate symptoms, the HAD scale was used to examine psychological profiles, and the Medical Outcome Short Form Health Survey questionnaire (SF36) and the Oral Health Impact Profile-14 (OHIP-14) were used to evaluate patient quality of life. The 12-week course of treatment was finished by 50 patients (26 in Group I and 24 in Group II). Although there were no statistically significant changes between the groups, both groups' visual analog scale pain values improved (P = 0·57). Additionally, oral quality of life increased. Six patients in Group II (placebo) and four in Group I (treatment) departed the study. During any of the evaluation periods, no patients had any negative side effects from their treatment. Because of noncompliance, patients were removed from the sample. It was discovered that the application of lycopene-enriched olive oil (Group I) had no effect on the lipid profile during the course of the three-month trial period, and the placebo group (Group II) also experienced no change	For the treatment of BMS patients, topical lycopene-enriched virgin olive oil is just as safe and effective as a placebo. To develop a treatment for people with chronic and painful illnesses, more research is necessary	(Cano‐Carrillo et al., 2014)

## Toothpastes containing various components

Zinc-containing drugs have gained popularity in dental treatments due to their potent antibacterial characteristics and capacity to stimulate mucosal healing (Uwitonze et al. 2020). Zinc citrate, zinc oxide, zinc chloride, and zinc gluconate are common ingredients in dental care regimens. Zinc citrate is commonly found in toothpaste and mouthwashes, where it inhibits bacterial development and so helps to minimize the development of plaque and gingivitis. Zinc oxide, which is extensively used in dental cements and fillings, has both antibacterial and remineralizing properties (Moradpoor et al. 2021). Zinc chloride, found in mouthwashes and topical gels, helps to manage oral infections and speeds up wound healing after dental treatments. Zinc gluconate is also helpful at reducing unpleasant breath and inhibiting bacterial proliferation (Pushpalatha et al. 2022).

Recent research has demonstrated the efficacy of zinc-containing medications in dental treatments, particularly in reducing bacterial development and boosting mucosal healing. For example, a study on the development of an injectable zinc-containing hydrogel with a double dynamic bond proved its efficacy in treating periodontitis. The hydrogel demonstrated outstanding osteogenic capabilities, self-healing ability, and broad-spectrum antibacterial activity, making it a viable local drug delivery system (Yang et al. 2024). Another study examined the development of zinc-containing chitosan/gelatin coatings with immunomodulatory properties for soft tissue sealing around dental implants. These coatings were reported to successfully induce pro-regenerative macrophage polarization while also increasing gingival fibroblast adhesion, proliferation, and collagen secretion (Han et al. 2024).

Furthermore, studies on the integration of zinc oxide nanoparticles displayed their ability to hinder acid generation by *Streptococcus mutans* and *Lactobacillus* in dental plaque, indicating their potential for avoiding dental caries and treating oral infections (El Shahawi 2023). Another study examined the use of zinc oxide nanoparticles in a variety of dental applications, including endodontics, restoratives, periodontics, implantology, orthodontics, and prosthodontics. These nanoparticles exhibited increased cytotoxicity, high selectivity, biocompatibility, and ease of manufacturing, making them useful in dental materials (Zeidan et al. 2022).

Meanwhile, a pilot randomized controlled trial assessed the efficacy of CAREDYNE Shield, an advanced zinc-containing desensitizer, in treating dentin hypersensitivity. The study discovered that CAREDYNE Shield effectively reduced the intensity of pain in response to air stimuli, which was comparable to other desensitizers (Matsuura et al. 2022). Further research explored the efficacy of dentifrice containing arginine and zinc in reducing dental plaque both in vitro and in vivo. The combination of these active chemicals was shown to be beneficial in eradicating dental plaque (Gloag et al. 2023).

In addition, salicylate-containing medications, such as aspirin (acetylsalicylic acid) and topical NSAIDs as diclofenac sodium and methyl salicylate, are often utilized in dental treatments owing to their analgesic and anti-inflammatory effects. These drugs can help treat oral pain, inflammation, and temporomandibular diseases (Jeske 2024).

Research findings have been conflicting, with some suggesting a decrease in the severity of pain and others pointing to a limited level of efficacy when compared to placebo treatments. When employing salicylate-containing drugs in dental care, it is essential to consider the response of each patient as well as possible adverse effects, like gastrointestinal discomfort (Panchanadikar et al. 2021). According to a systematic review, for example, although several topical therapies demonstrated promise, the limited number of studies and possible biases indicate that the total evidence is still equivocal. To confirm these results and provide precise recommendations for the use of drugs containing salicylate in dental procedures, more investigation is required (Wiśniewska et al. 2023). It is important to note that a previous study demonstrated the employment of choline salicylate and lidocaine hydrochloride in a new dental gel as a promising anti-inflammatory approach in various dental treatments (Maslii et al. 2021).

Furthermore, fluoride-containing medications play an important role in dental therapies since they can prevent dental caries and promote tooth remineralization. These drugs comprise many forms, including toothpastes, mouth rinses, gels, varnishes, and silver diamine fluoride (Sun et al. 2020). For example, fluoride toothpaste is commonly advised for daily usage to strengthen tooth enamel and lower the incidence of cavities. Professionally administered fluoride treatments, including varnishes and gels, contain a higher concentration of fluoride and are especially effective for individuals who are at high risk of dental caries (Saad et al. 2022). Additionally, community water fluoridation is a public health practice that raises the fluoride content of the water to appropriate levels to prevent tooth decay. These fluoride treatments have been recognized for their safety and efficacy in maintaining oral health (Jurasic et al. 2022). Several studies have demonstrated the use of topical fluoride solutions, namely silver diamine fluoride, in dental treatment as an anti-hypersensitivity agent and putative caries-preventing formulation (Gao et al. 2021; Horst 2018; Mei et al. 2016; Zheng et al. 2022).

Besides, a previous study investigated the efficacy of a localized sustained fluoride release from personalized 3D printed mouthguards at the device enamel interface which proved to enhance the integration of fluoride inside the tooth matrix and avoid lesion progression (Berger et al. 2022). Further, the dual action of fluoride and nitric oxide-releasing hydrogels was reported with enhancement in combating dental caries (Estes Bright et al. 2022). Also, topical application of casein phosphopeptide–amorphous calcium phosphate comprising fluoride proved to be an effective treatment approach for children with caries combating dysbiotic oral microbiome which contributes to dental caries pathogenesis in children (Widyarman et al. 2021).

Dental medications containing salicylate have been reported to offer anti-inflammatory and analgesic effects, especially in cases of periodontal diseases (Kotowska-Rodziewicz et al. 2023). However, long usage can present complications such as local inflammation as well as various hypersensitivity reactions in some patients (Nagi et al. 2015). Various toothpaste containing different types of fluoride act effectively in preventing dental caries through aspects of enamel depositional rebinding and bacterial suppression (Mahmoud et al. 2020). Several research studies have also shown that fluoride in alloys with zinc or bioactive glass nanoparticles widened the positive impact of bacterium eradication and decreased the number of oral bacteria and plaques compared with fluoride alone (Cui et al. 2023; Satpathy et al. 2023). Nevertheless, excessive use of fluorides in the form of oral products results in dental fluorosis particularly in children and therefore needs close monitoring (Toumba et al. 2019).

## Applications of herbal products in dentistry

Herbal extracts are concentrated substances derived from plants, containing active compounds that can offer therapeutic benefits (Ashfaq et al. 2023). However, many herbal extracts face challenges with solubility and bioavailability, which limit their effectiveness in treatment of several orodental disorders. To enhance these properties, several strategies were employed (Hani et al. 2023), among which were nanotechnology-based formulations. Nanotechnology-based delivery systems, such as lipid nanoparticles, polymeric nanoparticles, and hybrid nanoparticles can significantly enhance the solubility, stability, and extend the release of herbal extracts, promoting effective absorption across biological membranes (Wassif et al. 2024). Additionally, using permeability enhancers, enteric coatings, bioenhancers, and solubilizing agents such as cyclodextrins can further boost the solubility and bioavailability of these herbal extracts (Sodeifian and Usefi 2023).

Consequently, several reports revealed the formulation of phytochemicals into nano-based systems to foster their therapeutic efficiency for treatment of orodental disorders (Nsairat et al. 2023). This will be listed in the following.

Periodontitis and dental caries are the most prevalent oral infections. It was discovered that invasion by pathogenic bacteria is the primary cause of these conditions. These bacteria sequester inside the extracellular matrix, preventing the penetration of antibacterial agents and forming biofilms. Essential oils possess the functionality to be quite beneficial for the management of dental infections (Di Stefano et al. 2022). Though, a challenge occurs as bacterial cells are surrounded by a hydrophilic extracellular matrix which is impervious to essential oils. To overcome the development of resistance, the approach of nanotechnology has been effectively applied, with a remarkable increase in antibacterial action (Mubeen et al. 2021).

In a previous study, Poly (D, L-lactide-co-glycolide) (PLG) nanoparticles laden with *Harungana madagascariensis* extract were synthesized and investigated for their antimicrobial effects against Gram-negative and Gram-positive strains that inhabit the oral cavity. The nanoformulations demonstrated a significantly lower minimum bactericidal concentration (1.875 × 102 mg/L) relative to extracts in ethyl acetate (5–7 × 102 mg/L). The bioadhesive characteristic of the PLG polymer enabled nanoparticles to adhere to bacterial cells, resulting in the extract's sustained release while preserving the concentration (Kumar et al. 2022).

Similarly, biofilms are hydrophilic in nature, therefore lipophilic essential oils can be transformed into nanoemulsions of size smaller than 300 nm, allowing substances to penetrate the biofilm matrix. A study displayed that cinnamon oil and grapefruit seed extract encapsulated in nanoemulsions reduced the biofilm of *Streptococcus mutans* by 86% relative to 60% for the oil solution in ethanol (Choi et al. 2023).

In parallel, studies have proposed that nano/microemulsions provide superior outcomes in terms of resistance to bacteria (Subbiah et al. 2019). The combination of the nanosize feature and surfactants in the formulation supplied the delivery system with a high wetting ability and surface tension. This permitted fusion with the microbes' cell membranes, ultimately leading to their mortality.

Meanwhile, *Aloa vera*-loaded nanomaterials showed enduring antibacterial action in endodontic infections, a condition in which standard irrigation solutions and intracanal treatments used in root canal therapy are unable to completely eradicate the germs from the root canal (Beigoli et al. 2021). Additionally, the fabrication of silver, gold and bimetallic (gold and silver) nanoparticles allowed for substantial use of neem leaf extract (Borah et al. 2018). In the process of creating neem leaf extract loaded silver nanoparticles, the extract reduced and capped the adherence and growth of oral infections while also preventing secondary caries.

Similarly, in oral drug delivery systems, green tea catechin derivative nanoparticles demonstrated promising results. The maximum growth inhibition of oral *Fusobacterium*, *Actinomyces*, *Prevotella*, *Lactobacillus*, *Streptococcus*, *Propionibacterium* species was demonstrated by gallic acid loaded-PLG nanoparticles, indicating their efficacy in treating various bacterial infections that cause orodental diseases (Dalir Abdolahinia et al. 2023).

In another trial, the Chinese herbal remedy icariin—which was exploited to prevent osteopenia—was shown to prevent peri-implantitis and inflammation as well. The process of sequestering it into nanoparticles significantly sped up the incorporation of dental implants, increasing their efficiency (Liang et al. 2021). Additionally, *Mangifera indica*-loaded nanoparticles had shown encouraging antibacterial activity and had been propitiously applied in several dental applications, enhancing the hardness and mechanical bonding of the glass ionomer dental cement (Soesanto et al. 2023).

Meanwhile, *Moringa oleifera*-loaded casein phosphopeptide nanoparticles were also utilized to treat white spots, erosion, and dentin hypersensitivity. This new mixture displayed antimicrobial activity and dentin remineralization (Shafiq and Mahdee 2023). Furthermore, in another study, the manufacture of nano-hydroxyapatite with Moringa oleifera leaf extract showed remarkable nonlinear properties, including reducing and stabilizing action (Anwar et al. 2024).

Curcumin exhibits strong anti-inflammatory properties; yet, due to its hydrophobic nature, it experiences minimal oral absorption when utilized as a powder or in other traditional carriers. As a result, nanomicelles were developed encapsulating curcumin in a hydrophobic core. The delivery approach improved curcumin's solubility, rendering it effective in lowering inflammation in mild periodontitis and gingivitis (Bapat et al. 2023).

Nanotechnology has been shown to improve the stability of plant extracts against hydrolysis, oxidation, and photo or thermal degradation, as well as minimize volatility (Antunes Filho et al. 2023). Curcumin was reported to be photoreactive, reducing its activity by 70%. Therefore, Onoue et al. (Onoue et al. 2013) created curcumin loaded solid dispersions with enhanced physical stability, in which only 17% degradation was detected, hence increasing its effectiveness in gingivitis and mucositis treatment.

Moreover, Tonglairoum et al. (Tonglairoum et al. 2016) also demonstrated that complexing betel oil and clove oil with cyclodextrins increased their solubility. They were then integrated into nanofibers that developed rapid oil release and boosted antifungal efficacy against oral *Candida* species, demonstrating their potential for treating denture stomatitis.

In another study, researchers created microspheres loaded with zedoary oil derived from turmeric. It was shown that the small size of the carrier permitted greater in-vivo absorption, increasing bioavailability by 135.6% (Pandey et al.2022). Furthermore, the prolonged release of the employed oil minimized the undesirable side effects and lowered dose frequency. Also, baicalein was synthesized as a nanocrystal in another attempt, and the outcomes showed 1.67-fold increase in solubility and bioavailability (Dhaval et al. 2020). Both delivery systems showed enhanced therapeutic efficacy against periodontitis.

A variety of herbal extracts have been incorporated into nanosystems to treat periodontitis. Kaempferol nanoparticles loaded with calcium sulphate composite beads were recently developed and shown to exert antibacterial action against *Escherichia coli* and *Staphylococcus aureus*, indicating their usefulness in periodontal treatment to lower the level of bacteria at the area of infection (Narang and Narang 2015).

Previously, additional research carried out on dogs with periodontal abnormalities and treated with luteolin-loaded nanoparticles demonstrated high absorption across the junctional epithelium (Zhu et al. 2024). Additionally, a unique strategy involved sequestering apigenin-loaded polycaprolactone nanofibers for periodontal tissue regeneration at the gum interface. The researchers proved that encapsulating apigenin-loaded nanofibers with submicron diameters into the nanofibers' network reduced the release of apigenin from the nanoparticles, resulting in its extended release inside the periodontal pocket throughout the duration of the therapy. Initial investigations on human mesenchymal stem cells revealed cell survival up to 5 days after culture, hence suggesting the potential of these nanofibers in treating periodontal diseases (Mohammadinejad et al. 2020).

One of the most major implications of nanotechnology-based treatments is cancer therapy, which might otherwise cause numerous side effects and be prohibitively expensive. Recently, various novel revolutionary drug delivery systems have been discovered imparting nanoparticles laden with resveratrol, which could be an important advancement in avoiding periodontal disease development (Sharifi-Rad et al. 2021). Peppermint oil has been shown to provide considerable anticancer capabilities against oral cancer; yet its low solubility hinders its use. Lopes et al. (Lopes et al. 2022) established peppermint oil-loaded nanoemulsions with droplet size around 100 nm and then incorporated into the internal hydrophobic core with an external aqueous compartment, rendering them soluble in aqueous media.

In addition, herbal extracts from *Trypterygium wilfordii* showed promising results as oral chemotherapy treatments, although being water-insoluble and having limited intestinal absorption. A study reported that lipid-based nanocarriers as lipid nanospheres (Wagh et al. 2021) and lipid nanoparticles (Guo et al. 2021), laden with *Trypterygium wilfordii* were developed to improve its solubility and diffusivity into tumor cells. Moreover, phytosomes loaded with protamine and integrated into chitosan sponges were developed. This carrier system exhibited mucoadhesive attributes along with improved penetration across the buccal mucosa, resulting in a 90% increment in bioavailability for the management of numerous forms of oral malignancies (Lagoa et al. 2020).

Furthermore, researchers introduced the herbal shotgun strategy, in which synergistic mixtures of virgin coconut oil and peppermint oil loaded into nanoemulsions demonstrated tremendous cytotoxic functions towards oral squamous cancer cell lines (Kumar et al. 2022).

In parallel, silica nanoparticles laden with curcumin showed greater cellular uptake and cytotoxicity in oral squamous cell carcinoma cells relative to free curcumin dispersion. Curcumin-loaded nanoparticles proved to be more cytotoxic compared to free curcumin forms, promoting apoptosis in cancer cells (Pecorini et al. 2023).

Furthermore, naringenin-loaded nanoparticles possessing a cationic copolymer increased the solubility of naringenin (Budi and Farhood 2023). In a dimethoxybenzaldehyde-induced oral squamous cell carcinoma hamster model, they showed significant reduction in proliferation of tumor while increasing antioxidant levels. A further study displayed that the employment of chitosan-coated nanoemulsions encapsulating genistein that were incorporated into buccal tablet forms. Cytotoxicity studies on these cell lines demonstrated that the employed nanoemulsions constituted remarkable cytotoxic effectiveness than genistein conventional formulations (Umapathy et al. 2023).

Furthermore, the dried root of *Saliva miltiorrhiza* was previously employed in conventional Chinese medicine for the treatment of a variety of ailments, particularly cancer, and its loading into transfersomes demonstrated effectiveness to inhibit the proliferation of oral precancerous as well as oral squamous cell carcinoma cell lines (Qin and Wu 2023). Moreover, another study reported that *Bacillus subtilis*, *Escherichia coli*, *Candida albicans*, *Staphylococcus aureus*, and *Bacillus cerevisiae* from oral squamous cell cancer cell lines showed absence of resistance to flower extract of *Carthamus tinctorius-*loaded silver nanoparticles (Vyas et al. 2023).

Meanwhile, *Dendropanax morbifera* Le'veille, an ancient medicinal plant prevalent in South Korea, was employed for treatment of several types of cancers including oral ones. Fortunately, silver nanoparticles loading this plant extract was employed as carriers for its delivery to manage oral cancer, showing potent superior results as well as non-cytotoxicity to normal cells (Talukdar et al. 2023).

A further study reported that various plant extracts such as *Tamarix gallica*, *Terminalia chebula*, *Dendropanax mobifera* Le´veille, and ginseng aqueous leaf extract with reported antitumor action were additionally loaded into the environmentally friendly crystalline gold and silver nanoparticles with proven functionality against several types of oral cancers (Manikkath et al. 2023). Additionally, a number of green nanoparticles have been shown to exhibit potent antimetastatic, cytotoxicity and anticancer activities against a variety of oral cancer cell lines (Karnjana et al. 2023). These include gold nanoparticles encompassing the edible mushroom *Pleurotus florida*, *Erythrina suberosa*, *Couroupita zizanioides* ethanolic extract, *Dysosma pleiantha* rhizome aqueous extract, *Polygala senega* ethanolic extract nanoencapsulated form, and gold nanoparticles laden with Siberian ginseng.

Simultaneously, *Pluchea indica* extract-loaded nanoparticles increased both the migration of oral squamous carcinoma cells and cytotoxicity at low concentrations, and their oral nanoformulations improved the extract's colloidal stability (Mali 2023). Furthermore, green synthesized metal nanoparticles have shown tremendous effectiveness in the management of oral cancer and oral microbial infections. Copper oxide nanoparticles loaded with an aqueous bean extract were reported to promote mitochondrial apoptosis, decrease HeLa cell growth, and demonstrate in vitro chemotherapeutic action against oral squamous cell carcinoma (Sharma et al. 2024).

Besides, coriander sativum-loaded silver nanoparticles were found to possess increased antibacterial action towards common oral pathogenic organisms (Peng et al. 2023). Furthermore, artichoke extract loaded nanoparticles displayed significant reduction in oral premalignant lesions (Imbesi Bellantoni et al. 2023). Meanwhile, nanoparticles generated from guava leaves demonstrated effectuality to suppress the proliferation of oral cancer cells, eliminating up to 80% of them (Nsairat et al. 2023).

In addition, hydroxyapatite has been proven to demonstrate bone-forming features and is thus employed in several dental implants as a bone substitute. In one study, it was observed that hydroxyapatite-sequestered nanocrystals induced bone remineralization, yet only in the enamel's outer layer (Angellotti et al. 2023). Moreover, Imran et al. (Imran et al. 2023) discovered that combining both Gallachinensis extracts and nanohydroxyapatite improved bone deposition and remineralization on teeth relative to single procedures.

Further, nanocomposites have found widespread utilization in dentistry. They could create a more robust and natural contact between the hard mineralized tissues of the tooth and the small filler particles employed by improving consistency between the structure of the tooth and these proficient regenerative materials. These nanofillers showed their potential to provide flexural strength, superior hardness, translucency, elasticity, exemplary handling characteristics as well as a 50% reduction in filling shrinkage (Aktas et al. 2024).

Fortunately, nanotechnology was also projected to contribute to significant advancements in digital dental imaging procedures in addition to its therapeutic properties. High-quality images could be obtained with a reduced radiation dosage in digital radiographies that employ nanophosphor scintillators (Haidar 2023).

Several approved oral medications used in dentistry are listed as follows:

**Table:** 

Listerine mouthwash	Essential oils based (thymol, eucalyptus oil, menthol and methyl salicylate)	Mouthwash for oral hygiene and plaque control	Marinković et al. (2023)
Parodontax toothpaste	Herbal extracts (sage, chamomile and thyme)	Toothpaste to reduce plaque and gum diseases	Hotwani et al. (2014)
Biotene	Glycerin, xylitol, sorbitol, propylene glycol	Saliva substitute for dry mouth	Barbe et al. (2019)
Hemp toothpaste fluoride free	Hemp oil	Anti-inflammatory toothpaste	Alkan et al. (2023)
Neem toothpaste	Neem extract	Antimicrobial toothpaste	Srichan et al. (2021)
Colgate Herbal toothpaste	Herbal extracts (sage, *Aloe vera* and chamomile)	Antibacterial toothpaste	Nova et al. (2021)
Aloe vera Gel	*Aloe vera*	Controlling bacteria that cause cavities	Fani and Kohanteb (2012)
Dabur Herbal Toothpaste	Clove	Prevents toothache and plaque	Chaudhary et al. (2020)
Aloedent	*Aloe vera*	Toothpaste and mouthwash	Abullais et al. (2022)

## Conclusion and future perspectives

Incorporating herbal dentifrice into daily oral hygiene can greatly benefit individuals with oral problems by effectively reducing plaque, mouth ulcers, oral carcinoma, and inflammation. Their anti-inflammatory properties help to reduce the gum inflammation, which is essential for managing periodontal diseases. Many herbs possess antibacterial effects that can inhibit the growth of periodontal pathogens, including antibiotic-resistant strains, thus preventing plaque formation and gum infections. Additionally, the antioxidant activity of various plants combats oxidative stress in oral tissues, promoting overall gum health. Herbal remedies offer notable advantages, including accessibility, cost-effectiveness, prolonged effects, and lower toxicity compared to conventional treatments.

Although many plants demonstrate low toxicity and promising pharmacological effects, there is a lack of comprehensive studies on their dental applications. Comprehensive preclinical and clinical studies are essential to ensure the safety and biocompatibility of these treatments. The demand for innovative substances to inhibit bacterial growth and biofilm formation in dental treatments underscores the necessity for additional research to validate these natural products' efficacy and safety in clinical settings.

## Data Availability

Not applicable.
